# Formation of Pyramidal Palladium Enhanced by B(C_6_F_5_)_3_ Additive Enabling Selective Alkene Monoisomerization

**DOI:** 10.1021/acsomega.5c09607

**Published:** 2025-12-09

**Authors:** Paul D. Miller, Trandon A. Bender

**Affiliations:** Department of Chemistry and Biochemistry, 6042 Old Dominion University, 4501 Elkhorn Ave, Norfolk, Virginia 23529, United States

## Abstract

The addition of a strong Lewis acid, tris­(pentafluorophenyl)­borane, facilitates the reduction of Pd­(cod)­Cl_2_ with Et_3_SiH to form palladium nanoparticles in organic solvents. It was found that these palladium nanoparticles are capable of selective monoisomerization of linear alkenes. This heterogeneous palladium catalyst is tolerant of a wide variety of functional groups, confirmed by substrate and robustness screenings. It is also possible to utilize poly­(methylhydro)­siloxane, an industrial waste byproduct, as the reductant to enable similar isomerization chemistry.

## Graphical abstract

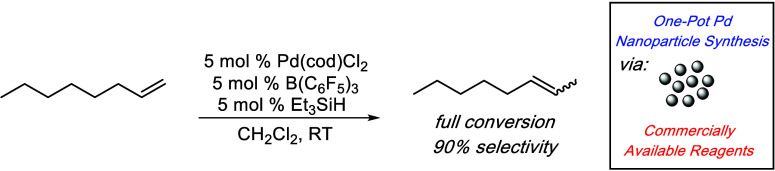

## Introduction

Controlling the homogeneous catalytic isomerization of terminal olefins has been a decades-long goal for chemists. While substrates exist with either sterically hindering substituents or a thermodynamic sink to favor a specific product,^1–4^ this does not account for linear, terminal olefins that have multiple, equally thermodynamically favorable products upon isomerization, which makes selectivity a challenge (Figure 1A).^5^ However, there are examples in the literature where homogeneous catalysts have achieved varying degrees of isomerization selectivity.^6–14^

**Figure 1:**
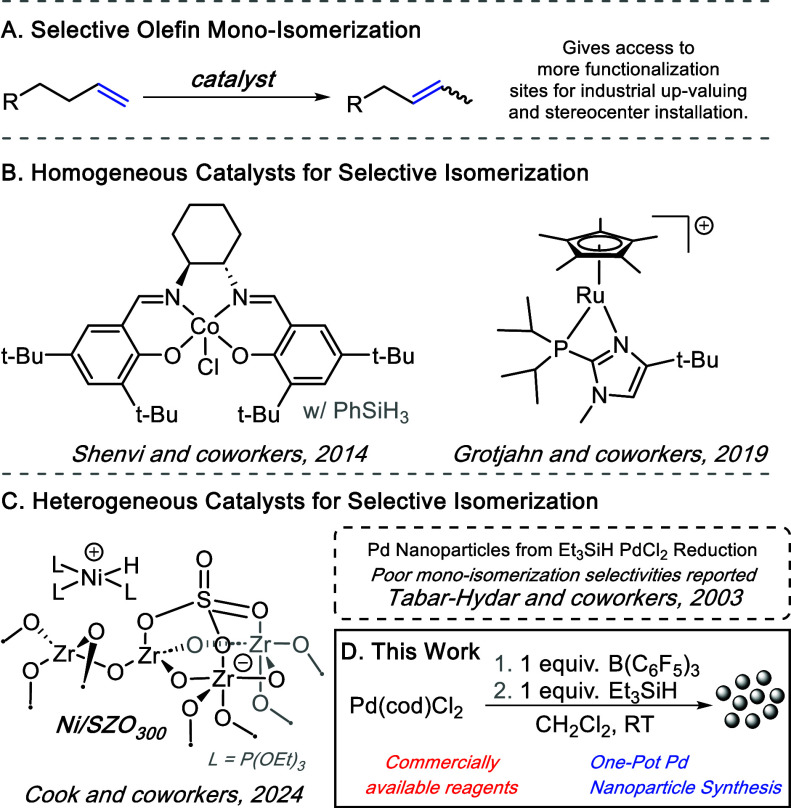
(A) Monoisomerization of terminal olefins through catalysis; (B) examples of homogeneous catalysts that gave high monoisomerization selectivity (m. i. s.); (C) past examples of heterogeneous systems that isomerized olefins, with Ni/SZO_300_ showing exceptional m.i.s., and past work on palladium nanoparticle generation through silane reduction giving poor results; (D) depiction of the work conducted herein, where reactive for olefin isomerization Pd nanoparticles can be generated from commercially available reagents in one pot.

Work by Dobereiner et al. took advantage of a Mo catalyst to perform monoisomerization of terminal olefins selectively through a 1,3-allyl shift mechanism.^15^ Alternatively, Shenvi et al. found a Co catalyst that enabled chemoselective radical isomerization of 1-decene to give 83% 2-decene (Figure 1B).^16^ Additional examples include the introduction of sterics on the catalyst to bias monoisomerization of terminal olefins, which has been a successful strategy.^13,17–20^ Notably, Grotjahn et al. found a homogeneous Ru catalyst that enables exceedingly selective monoisomerization (>92–98% selective for all but one example) with a large scope of terminal olefins with high *E* selectivity (Figure 1B).^18^ Finally, there has been promising work in the field of *contra*-thermodynamic control for the isomerization of alkenes, enabling movement of alkenes into traditionally disfavored locations.^21,22^

Previous work in our lab has shown that it is possible to abstract a chloride from Grubbs I with tris­(pentafluorophenyl)­borane (BCF) and Et_3_SiH, which performs selective alkene monoisomerization with a cocatalytic H-BCF^–^ counteranion.^23,24^ However, our work found that the substrate scope was a shortcoming in this example. While searching for catalysts that could tolerate more functionality in substrates, we discovered that BCF promotes the formation of a reactive Pd catalyst from Pd­(cod)­Cl_2_ in combination with Et_3_SiH (Figure 1D). With this combination, the isomerization of 1-decene to 2-decene is observed with 90% selectivity (*E*/*Z* = 1.9). While heterogeneous systems have been used to facilitate olefin isomerization,^25–28^ many suffer from nonselective isomerization and/or competing hydrogenation reactivity. Two notable heterogeneous alkene isomerization reactions include the polymer-bound ruthenium catalyst by Erdogan and Grotjahn^28^ and the more recent heterogeneous Ni–H catalyst reported by Cook and co-workers (Figure 1C).^25^ The work reported herein provides an operationally simple process to form reactive Pd nanoparticles using commercially available reagents in organic solvents. Moreover, the reactivity offers good substrate compatibility and a robustness screening that shows promising results for a wide variety of compounds, even those containing functional groups like thiols, which typically poison heterogeneous catalysts.

## Results and Disscusions

### Isomerization Condition Optimization and Substrate Screening

The process to prepare the palladium nanoparticles is straightforward: to a 1:1 solution of Pd­(cod)­Cl_2_ and BCF in CH_2_Cl_2_ is added 1 equiv of Et_3_SiH. The vial is then immediately sealed with a septa cap and mixed. Olefin substrate is then added via a syringe through the septum cap. After the reaction is complete, the mixture is filtered through silica gel to remove metal products. The reaction conditions were optimized with 1-decene (**1**) as the substrate, which is typically challenging to control. Under optimal conditions, 5 mol % of Pd­(cod)­Cl_2_, BCF, and Et_3_SiH gives 96% conversion with 90% monoisomerization selectivity (m.i.s.) for *E*- and *Z*-2-decene (**2** and **3**, respectively) and 10% decenes (**4**) in 2 h at room temperature (Table 1, entry 1).

**Table 1:**
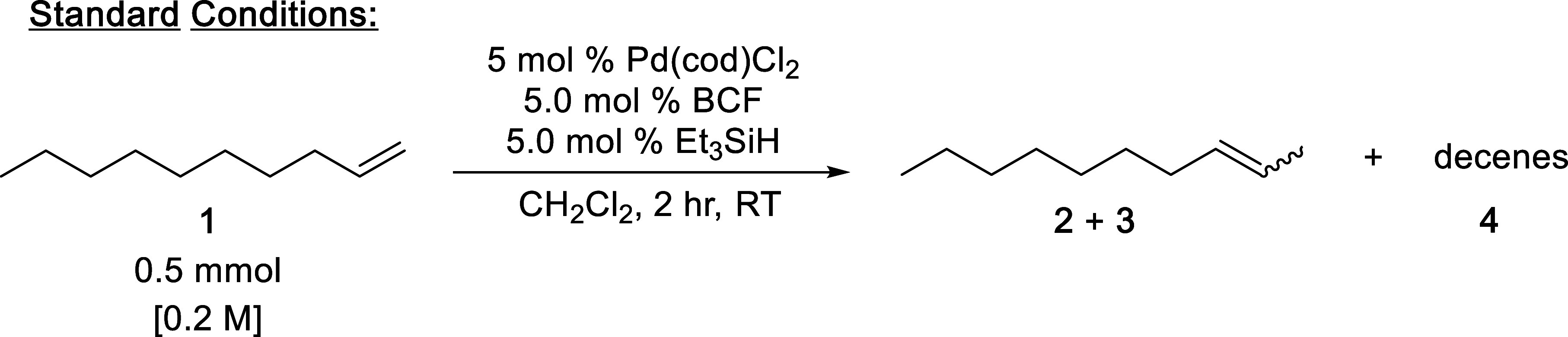
Reaction Optimization Conditions for the Pd-NP Alkene Isomerization

entry	deviation from standard conditions	% conversion	% m.i.s^a^	*E*/*Z*
1	none	96	90	1.9
2	no Et_3_SiH	10	40	3.1
3^b^	no BCF			
4^b^	no BCF or Et_3_SiH			
5	2.5 mol % Pd(cod)Cl_2_	92	90	1.7
6	2.5 mol % Pd(cod)Cl_2_/BCF/Et_3_SiH	87	92	1.5
7	20 mol % Et_3_SiH	32	89	1.4
8	dichlorethane as solvent	98	88	1.8
9	PMHS instead of Et_3_SiH	99	89	1.9
10	24 h instead of 2 h	99	79	3.6

a % Conversion, % monoisomerization selectivity (m.i.s), and *E*/*Z* ratios were determined by GC-MS analysis.

b Conditions lead to no reactivity.

First, it was determined that the presence of both Et_3_SiH and BCF was required for the reaction to take place efficiently (Table 1, entries 2 and 3). It is known that BCF can isomerize olefins,^29^ albeit at high temperatures. However, under catalytic BCF conditions without Pd or silane, no isomerization is observed at extended reaction times at room temperature, as found in our previous work. Moreover, we found that Pd­(cod)­Cl_2_ is also not competent as an isomerization catalyst precursor without the presence of BCF and silane (Table 1, entry 4).

We also found that the amount of Pd­(cod)­Cl_2_ could be reduced to 2.5 mol %, while maintaining 5 mol % BCF and Et_3_SiH, with minimal effect on reactivity and selectivity (entry 5, Table 1). Fully reducing the loading of all components to 2.5 mol % led to diminished conversion but maintained selectivity for monoisomerization (entry 6, Table 1). It was also found that the equivalents of Et_3_SiH relative to Pd­(cod)­Cl_2_ are important for reactivity. Increasing the amount of Et_3_SiH relative to Pd and BCF loading reduced reactivity (entry 7, Table 1). We then screened a series of solvents in place of CH_2_Cl_2_. However, dichloroethane was the only viable alternative (Table 1; entry 8; see the SI pg. S5 for other screened solvents). Interestingly, we were also able to substitute poly­(methylhydro)­siloxane (PMHS), an industrial waste byproduct, for Et_3_SiH as the reductant and still maintain good reactivity and selectivity (Table 1, entry 9). We also wanted to see if selectivity was dependent on the reaction time to understand if selectivity diminished after prolonged exposure to the catalysts, and found that after 24 h, only a minor decrease in selectivity was observed (Table 1, entry 10).

With the optimal conditions in hand, we sought to expand this method to additional substrates (Figure 2). We found that a variety of substrates could be efficiently isomerized under our conditions. The reaction continued to work at room temperature, and only a few of the substrates tested required extended reaction times when certain heteroatoms were present (Figure 2, **11**, **12**, **17**, and **18**). A few notable examples from the substrate screening are substrates containing halogen functionality that remain active and do not decompose under the reaction conditions (Figure 2, **7** and **8**). We were also able to demonstrate that even in the presence of a thermodynamically favored isomer, like conjugation with an aromatic ring, reactivity remains high for monoisomerization (Figure 2, **15** and **16**). Interestingly, allyl benzene (**14**) gave divergent *E*/*Z* selectivity that was verified over multiple runs, but the reason for this observation is unclear and will be explored further in future work. The catalyst is amenable to free alcohols (**9**) and the presence of a thiophene (**17**) in the substrate, although the chemistry occurs at a slower rate. Lastly, we tested monoisomerization of a functionalized ibuprofen and found that the reaction remains selective and efficient (Figure 2, **18**).

**Figure 2:**
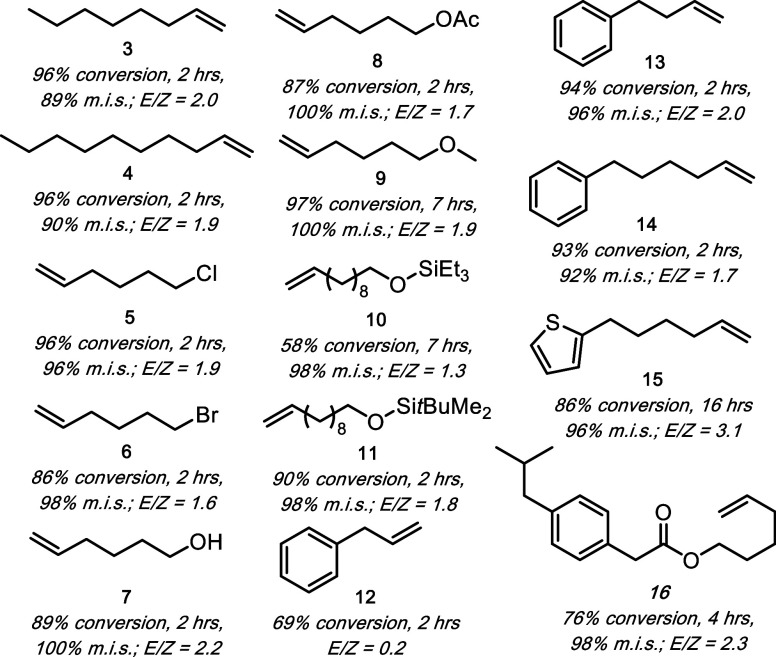
Substrates and the % conversion, % m.i.s., *E*/*Z* ratio of the monoisomerized product, and the reaction times. For each substrate standard conditions were used. % Conversion, % monoisomerization selectivity (m.i.s.), and *E*/*Z* ratios were determined by GC-MS analysis.

Instead of screening additional substrates beyond those in Figure 2, we opted to utilize the Glorius robustness screening protocol to understand the full scope of functional group compatibility.^33^ As shown in Figure 3, the catalyst remains active in the presence of many functional groups. However, there are a few that were not well tolerated. Specifically, nitrogen-containing functional groups like amides, amines, and pyridines reduce or shut down chemistry (Figure 3, entries 2, 5, and 11), but nitriles and indoles do not diminish reactivity (Figure 3, entries 3 and 14). Additionally, alkynes are incompatible, which we propose is a result of the heterogeneous Pd being strongly coordinating to the pi system (Figure 3, entries 15 and 16). This is supported by the observation that upon addition of alkyne substrate, the heterogeneous particles return to solution. Overall, the robustness screening demonstrated that the catalyst remains reactive toward a wide variety of different functional groups.

**Figure 3:**
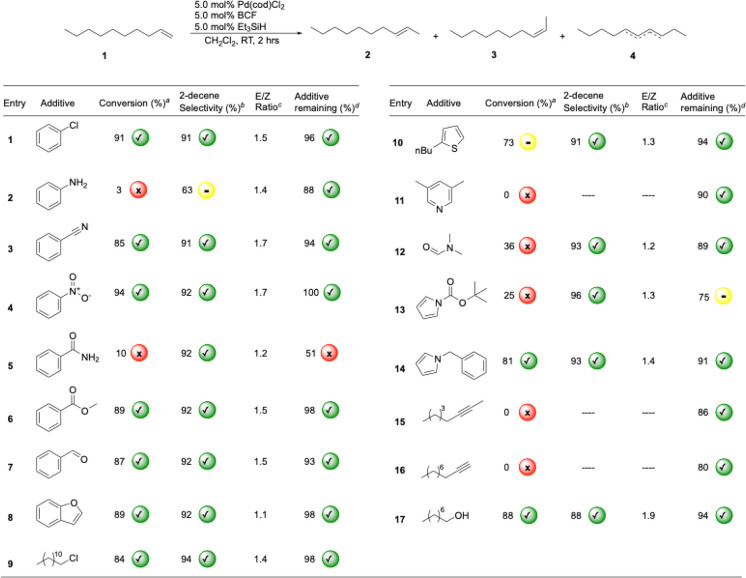
Decenes are defined as 3-decene, 4-decene, and 5-decene (4). Conversion, 2-decene selectivity, and *E*/*Z* ratio are determined by relative areas from GC-MS traces. ^a^Calculated from the percent total area from 2, 3, and 4. ^b^Calculated from the percent total area of 2 and 3 relative to the total isomerized products. ^c^Calculated by dividing the area of 2 by the area of 3. ^d^Calculated by comparing the ratio of the mesitylene internal standard (I.S.) to the additive in the reaction mixture to the ratio obtained from a standard equimolar solution of the mesitylene I.S. and respective additive.

### Identifying Source of Catalysis

Next, we sought to understand the nature of the catalyst. Specifically, if the reaction was proceeding under hetero- or homogeneous catalysis. Visually, when Pd­(cod)­Cl_2_, BCF, and Et_3_SiH are combined in CH_2_Cl_2_, black solids fall out of solution after mixing. Moreover, there are no diagnostic peaks by NMR left in the supernatant, besides excess silane and cyclooctadiene. These observations indicated that heterogeneous catalysis was operative in these reactions. To test this, we formed the heterogeneous palladium particles via our BCF/Et_3_SiH method and then filtered the reaction through a syringe filter. The filtered supernatant gave a reduced conversion of 34% 1-decene to 2-decene:decenes, with 88% m.i.s. This diminished reactivity indicated the removal of some of the active catalyst by filtration, suggesting a heterogeneous catalyst. Given that a micrometer filter was used, smaller heterogeneous particles could have passed through to continue catalysis at a diminished rate.

Next, we isolated the formed heterogeneous palladium particles that had settled to the bottom of the reaction vessel, washed them with CH_2_Cl_2_ to remove any potential homogeneous catalysts, and then used this isolated material to isomerize 1-decene. This reaction gave 46% conversion with a 95% m.i.s., which is diminished reactivity relative to the optimal conditions but demonstrates that the heterogeneous particles are catalytically competent. Lastly, we performed a Hg^0^ drop test and found that only 10% of 1-decene was isomerized.^30,31^ It should be noted that Hg^0^ drop tests alone are not enough to verify the heterogeneity. The Hg^0^ drop test, as originally proposed, tests whether Hg^0^ can form an amalgam with a metal atom unprotected by ligands. Recently, however, it has been shown that homogeneous organometallic complexes are reactive with Hg^0^, making this test, as the sole arbiter of heterogeneity, unreliable in some instances.^32^

The observation of reactivity postfiltration and with the addition of Hg^0^ required additional verification that the catalyst was heterogeneous. Given the remaining reactivity after filtration, this suggested that small amounts of active particles may remain suspended in solution. To test this, we prepared and filtered the catalyst. The supernatant was then placed in an air-free centrifuge tube and centrifuged. After spinning down, a solid was observed at the bottom of the tube, demonstrating that not all the particles had been removed upon filtration. With this, we ran another Hg^0^ drop test where the catalyst was prepared in a centrifuge tube, centrifuged, and then Hg^0^ was added to ensure all solids were at the bottom of the tube. Substrate was then added, and no reactivity was observed. Lastly, dynamic light scattering (DLS) measurements were attempted, but no data are reported here because the formation of particles at the bottom of the cuvette over time means that the sample is changing during data acquisition, and therefore, the data are not reliable.

With these observations and the confirmation that full removal of solids gave no catalytic reactivity, we set out to characterize the nature of the heterogeneous particles. To do so, we turned to scanning electron microscopy (SEM). From these experiments it was found that the resultant particles were large nano- to micrometer in size and formed as pyramidal structures (Figure 4, SEM image, see the SI pg. S79). In addition to these pyramidal structures, some amorphous particles were also observed. It was also found that the reaction conditions influenced particle formation. A previous study that generated Pd nanoparticles with only silanes reported longer isomerization reaction times for linear olefins and limited selectivity for the monoisomerization products observed here.^26,27^ There were no prior SEM data visualizing these particles. Therefore, we prepared particles following the previously published conditions and observed particles that were more amorphous in nature than those generated in the presence of BCF and Et_3_SiH (see the SI pg. S80).

**Figure 4:**
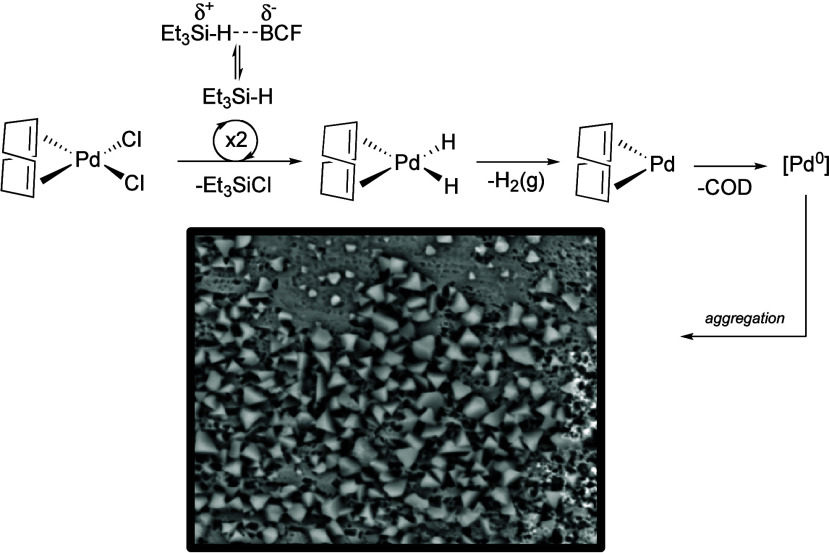
Mechanism of palladium nanoparticle formation via reduction with Et_3_SiH in the presence of BCF, which decreases the rate of Pd­(cod)­Cl_2_ reduction through forming a contact pair with Et_3_SiH. Overall, this controls the release of Pd^0^ into solution, allowing for controlled Pd^0^ aggregation to occur that is not observed without the presence of BCF generating reactive palladium nanoparticles.

With an understanding of the heterogeneous structure, we next sought to propose a mechanism for the heterogeneous catalyst formation and olefin isomerization mechanism. We had observed that when the equivalents of Et_3_SiH exceed the equivalents of BCF, reactivity is reduced (entry 7, Table 1). Also, upon close inspection of the products by GC-MS, we observed Et_3_SiCl, cyclooctadiene, and a small portion of the hydrogenated olefin substrate (less than 1%). Since the formation of ordered nanoparticles requires controlled reduction of metal,^34^ we propose that BCF modulates the rate of Pd­(II) to Pd(0) reduction by forming a contact pair with Et_3_SiH in solution (Figure 4).^35^ It is proposed that this interaction influences the rate of two successive σ-bond metathesis reactions of Et_3_SiH and Pd­(cod)­Cl_2_ to form Et_3_SiCl and L_*n*_Pd–H_2_.^36–39^ Reductive elimination of H_2_ (g) from (cod)­PdH_2_ yields (cod)­Pd, which can release an atom of Pd(0) upon dissociation of cod (Figure 4). These Pd(0) atoms then aggregate to form nanoparticles. We propose that the primary role of BCF is to modulate the rate of the initial silane activation steps that ultimately lead to the formation of H_2_ (g) and Pd nanoparticles. This mechanism may account for the divergent reactivity observed here compared to previous reports that did not use BCF during palladium reduction with silanes.^26,27^

With a proposed mechanism of nanoparticle formation, we then sought to understand the mechanism of olefin isomerization. We had previously observed that conducting the reaction in a sealed container was essential for isomerization to occur. Upon closer inspection of the GC-MS spectrum of an isomerization reaction of 1-decene, we detected a small amount of decane (<1% of material). Given the ability of heterogeneous palladium to catalyze alkene hydrogenation, this was unsurprising. However, the formation of decane without exogenous hydrogen further supports the hypothesis that H_2_ is generated during catalyst formation, which was further supported by observing H_2_ by ^1^HNMR spectroscopy under standard reaction conditions.

To test whether H_2_ (g) plays a role in the observed reactivity, we conducted a standard isomerization of 1-decene under an atmosphere of hydrogen. As expected, decane formation increased, and the monoisomerization selectivity dropped to 62%, compared to 90% under standard conditions. From this, we propose the following isomerization mechanism: the olefin substrate first binds to the nanoparticle surface, which has adsorbed H_2_ (g) formed during catalyst activation. A surface hydride undergoes insertion into the bound olefin, forming a palladium-alkyl intermediate. This is followed by β-hydride elimination, regenerating the surface hydride, and migrating the double bond one position down the chain. The product then dissociates, allowing another terminal olefin to bind (Figure 5). The high selectivity for monoisomerization suggests that terminal olefins are sterically favored to bind and react, while internal olefins are less likely to reassociate with the surface. This proposed mechanism is consistent with that proposed by Shon et al.^40^

**Figure 5:**
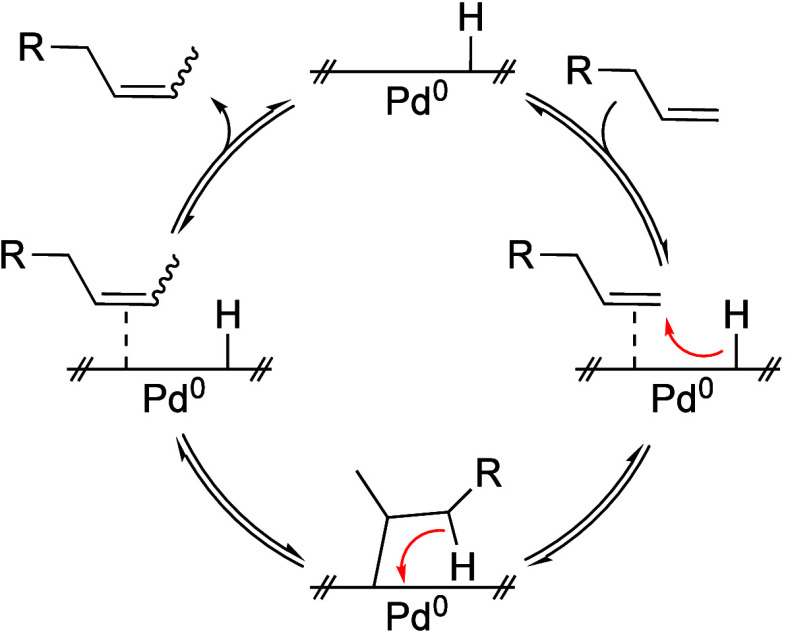
Proposed mechanism for the isomerization of terminal olefins at the surface of palladium nanoparticles with absorbed hydrogen.

## Conclusions

In conclusion, we report a heterogeneous Pd catalyst for the selective isomerization of linear aliphatic terminal alkenes. The synthesis of the heterogeneous Pd particles is procedurally simple, and the tolerance of a wide range of functional groups highlights the potential applicability of this method. The use of Lewis acid BCF introduces an alternative pathway for generating heterogeneous particles via silane-mediated reduction. Furthermore, the ability to form the heterogeneous catalyst using dichloroethane and PMHS, an industrial waste byproduct, offers a cost-effective alternative and eliminates the need for dichloromethane.

## Supplementary Material



## Notes

Experimental procedures, GC/MS, NMR, and SEM data (PDF)

## Abbreviations

cod: cyclooctadiene
BCF: tris­(pentafluorophenyl)­borane
Et3SiHd: triethylsilane

## References

[1] Larsen C. R., Grotjahn D. B. (2012). Stereoselective Alkene Isomerization over One Position. J. Am. Chem. Soc..

[2] Massad I., Marek I. (2020). Alkene Isomerization
through Allylmetals as a Strategic Tool in Stereoselective
Synthesis. ACS Catal..

[3] Liu X., Zhang W., Wang Y., Zhang Z. X., Jiao L., Liu Q. (2018). Cobalt-Catalyzed Regioselective
Olefin Isomerization under Kinetic
Control. J. Am. Chem. Soc..

[4] Hwan J. L., Smith C. R., RajanBabu T. V. (2009). Facile
Pd­(II)- and Ni­(II)-Catalyzed
Isomerization of Terminal Alkenes into 2-Alkenes. J. Org. Chem..

[5] Sharma A., Nair R., Gustafson J. L., Larsen C. R., Grotjahn D. B. (2007). Extensive
Isomerization of Alkenes Using a Bifunctional Catalyst: An Alkene
Zipper. J. Am. Chem. Soc..

[6] Larsen C. R., Erdogan G., Grotjahn D. B. (2014). General
Catalyst Control of the Monoisomerization
of 1-Alkenes to Trans −2-Alkenes. J.
Am. Chem. Soc..

[7] Zhong J., Wang X., Luo M., Zeng X. (2024). Chromium-Catalyzed
Alkene Isomerization with Switchable Selectivity. Org. Lett..

[8] Garhwal S., Kaushansky A., Fridman N., de Ruiter G. (2021). Part per Million
Levels of an Anionic Iron Hydride Complex Catalyzes Selective Alkene
Isomerization via Two-State Reactivity. Chem.
Catal..

[9] Wang Y., Qin C., Jia X., Leng X., Huang Z. (2017). An Agostic Iridium
Pincer Complex as a Highly Efficient and Selective Catalyst for Monoisomerization
of 1-Alkenes to Trans-2-Alkenes. Angew. Chem.,
Int. Ed..

[10] Camp A. M., Kita M. R., Blackburn P. T., Dodge H. M., Chen C. H., Miller A. J. M. (2021). Selecting Double Bond Positions with a Single Cation-Responsive
Iridium Olefin Isomerization Catalyst. J. Am.
Chem. Soc..

[11] Meng Q. Y., Schirmer T. E., Katou K., König B. (2019). Controllable
Isomerization of Alkenes by Dual Visible-Light-Cobalt Catalysis. Angew. Chem., Int. Ed..

[12] Becica J., Glaze O. D., Wozniak D. I., Dobereiner G. E. (2018). Selective
Isomerization of Terminal Alkenes to (*Z*)-2-Alkenes
Catalyzed by an Air-Stable Molybdenum(0) Complex. Organometallics.

[13] Kocen A. L., Klimovica K., Brookhart M., Daugulis O. (2017). Alkene Isomerization
by “Sandwich” Diimine-Palladium Catalysts. Organometallics.

[14] Tricoire M., Wang D., Rajeshkumar T., Maron L., Danoun G., Nocton G. (2022). Electron Shuttle in
N-Heteroaromatic Ni Catalysts for
Alkene Isomerization. JACS Au.

[15] Jenny S. E., Serviano J. M., Nova A., Dobereiner G. E. (2023). A Hydride
Migration Mechanism for the Mo-Catalyzed *Z*-2-Selective
Isomerization of Terminal Alkenes. ChemCatChem.

[16] Crossley S. W. M., Barabé F., Shenvi R. A. (2014). Simple, Chemoselective,
Catalytic Olefin Isomerization. J. Am. Chem.
Soc..

[17] Larsen C. R., Erdogan G., Grotjahn D. B. (2014). General Catalyst Control of the Monoisomerization
of 1-Alkenes to Trans-2-Alkenes. J. Am. Chem.
Soc..

[18] Paulson E. R., Moore C. E., Rheingold A. L., Pullman D. P., Sindewald R. W., Cooksy A. L., Grotjahn D. B. (2019). Dynamic π-Bonding of Imidazolyl
Substituent in a Formally 16-Electron Cp*Ru­(Κ2-P, N)+ Catalyst
Allows Dramatic Rate Increases in (E)-Selective Monoisomerization
of Alkenes. ACS Catal..

[19] Gelman D., Belkova N. V., Shubina E. S., Filippov D. Sc. O. A., De-Botton S. (2020). Regioselective Isomerization of Terminal
Alkenes Catalyzed
by a PC­(Sp^3^)­Pincer Complex with a Hemilabile Pendant Arm. ChemCatChem..

[20] Chen C., Dugan T. R., Brennessel W. W., Weix D. J., Holland P. L. (2014). Z. -Selective
Alkene Isomerization by High-Spin Cobalt­(II) Complexes. J. Am. Chem. Soc..

[21] Occhialini G., Palani V., Wendlandt A. E. (2022). Catalytic,
Contra-Thermodynamic Positional
Alkene Isomerization. J. Am. Chem. Soc..

[22] Palani V., Wendlandt A. E. (2023). Strain-Inducing Positional Alkene Isomerization. J. Am. Chem. Soc..

[23] Medley A. W., Patel D., Utne C., Bender T. A. (2024). B­(C6F5)­3 Co-Catalyst
Promotes Unconventional Halide Abstraction from Grubbs I to Enhance
Reactivity and Limit Decomposition. Organometallics.

[24] Miller P. D., Calkins J. B., Bayse C. A., Bender T. A. (2025). Metathesis or Isomerization:
Counteranion Directed Reactivity of Grubbs I. Organometallics.

[25] Chang A. S., Kascoutas M. A., Valentine Q. P., How K. I., Thomas R. M., Cook A. K. (2024). Alkene
Isomerization Using a Heterogeneous Nickel-Hydride
Catalyst. J. Am. Chem. Soc..

[26] Mirza-Aghayan M., Boukherroub R., Bolourtchian M. (2006). A Mild and Efficient Palladium-Triethylsilane
System for Reduction of Olefins and Carbon-Carbon Double Bond Isomerization. Appl. Organomet. Chem..

[27] Mirza-Aghayan M., Boukherroub R., Bolourtchian M., Hoseini M., Tabar-Hydar K. (2003). A Novel and
Efficient Method for Double Bond Isomerization. J. Organomet. Chem..

[28] Erdogan G., Grotjahn D. B. (2014). Supported Imidazolylphosphine
Catalysts for Highly
(E)-Selective Alkene Isomerization. Org. Lett..

[29] Morrill L.
C. B, Grayson M. N., Melen R. L., Linford-Wood T. G., Elsherbeni S. A., Kustiana B. A. (2022). (C6F5)­3-Catalyzed E-Selective Isomerization
of Alkenes. Chem. - Eur. J..

[30] Zhong H., Gong Y., Zhang F., Li L., Wang R. (2014). Click-Based
Porous Organic Framework Containing Chelating Terdentate Units and
Its Application in Hydrogenation of Olefins. J. Mater. Chem. A Mater..

[31] Rosu-Finsen A. (2023). A Mercurial Test.
Nat. Rev. Chem..

[32] Gorunova O. N., Novitskiy I. M., Grishin Y. K., Gloriozov I. P., Roznyatovsky V. A., Khrustalev V. N., Kochetkov K. A., Dunina V. V. (2018). When Applying the Mercury Poisoning Test to Palladacycle-Catalyzed
Reactions, One Should Not Consider the Common Misconception of Mercury(0)
Selectivity. Organometallics.

[33] Collins K. D., Glorius F. (2013). A Robustness Screen
for the Rapid Assessment of Chemical
Reactions. Nat. Chem..

[34] Xiong Y., Xia Y. (2007). Shape-Controlled Synthesis
of Metal Nanostructures: The Case of Palladium. Ad. Mater..

[35] Lowe J. M., Bowers B. E., Seo Y., Gagné M. R. (2020). Modulating
Electrostatic Interactions in Ion Pair Intermediates To Alter Site
Selectivity in the C–O Deoxygenation of Sugars. Angew. Chem., Int. Ed..

[36] Kirai N., Takaya J., Iwasawa N. (2013). Two Reversible σ-Bond Metathesis
Pathways for Boron-Palladium Bond Formation: Selective Synthesis of
Isomeric Five-Coordinate Borylpalladium Complexes. J. Am. Chem. Soc..

[37] Perutz R. N., Sabo-Etienne S., Weller A. S. (2022). Metathesis by Partner Interchange
in σ-Bond Ligands: Expanding Applications of the σ-CAM
Mechanism. Angew. Chem. - Int. Ed..

[38] Tilley T. D. (1993). The Coordination
Polymerization of Silanes to Polysilanes by a “σ-Bond
Metathesis”. Mechanism. Implications
for Linear Chain Growth. Acc. Chem. Res..

[39] Higashi T., Kusumoto S., Nozaki K. (2019). Cleavage of
Si–H, B-H, and
C–H Bonds by Metal-Ligand Cooperation. Chem. Rev..

[40] Zhu J. S., Shon Y. S. (2015). Mechanistic Interpretation of Selective
Catalytic Hydrogenation
and Isomerization of Alkenes and Dienes by Ligand Deactivated Pd Nanoparticles. Nanoscale.

