# Supplementary material for: Formation of Pyramidal Palladium Enhanced by B(C6F5)3 Additive Enabling Selective Alkene Monoisomerization
Source: ACS Omega. 2025 Dec 9;10(50):62176–81. doi: 10.1021/acsomega.5c09607 (PMC12750212; doi:10.1021/acsomega.5c09607)
Supplement: Supplementary file 1 [file ao5c09607_si_001.pdf]

Supporting Information for: Formation of Pyramidal Palladium Enhanced by $B(C_6F_5)_3$ Additive Enabling Selective Alkene Mono-isomerization

Paul D. Miller and Trandon A. Bender*

Table of Contents

General Experimental S1

Synthesis and Characterization S2

GC-MS Traces for Condition Optimizations, Substrate Scope… S5

SEM Imagine of Pd Particles S78

References S80

Spectra of Compounds S81

General Experimental

General Methods: All reactions, unless otherwise stated, were carried out in oven (130°C) dried glassware under inert atmosphere using standard Schlenk techniques. Unless otherwise specified, all reactions were conducted at ambient temperature (25°C, rt).

Chemicals and Materials: All reagents were used as received from commercial sources without further purification. All solvents, unless otherwise stated, were sparged with nitrogen for 30 minutes prior to use and stored over 4 Å molecular sieves, after being passed through activated alumina columns in an Inert PureSolv MD 7 solvent purification system. For filtrations, Basix™ Syringe Filters, PTFE, Non-Sterile from Fisher Scientific were used.

NMR: All ^{1}H, ^{2}H, ^{13}C, ^{19}F, and ^{31}P spectra were recorded on either a 400 MHz Bruker Avance spectrometer or 600 MHz Bruker Avance spectrometer. All deuterated solvents were used as received from Cambridge Isotope Laboratories, Inc. Chemical shifts are reported in parts per million (ppm) with the residual solvent protons used as internal calibration standards.[1] The following abbreviations are used in reporting NMR data: s, singlet; br. s., broad singlet; d, doublet; t, triplet; q, quartet; quint, quintet; sept, septet; dd, doublet of doublets; dt, doublet of triplets; dq, doublet of quartets; td, triplet of doublets; tt, triplet of triplets; quint d, quintet of doublets; ddd, doublet of doublet of doublets; and m, multiplet. TopSpin software were used in the processing of the spectra herein.

GC-MS: All GC-MS traces reported were collected using a Shimadzu GC-2010 Plus with a 30 m x 0.25 mm internal diameter, 0.25 μm film thickness DB-5 ms column, a Shimadzu GCMS-QP2010 SE detector and a Shimadzu AOC-20i auto injector. Analysis was performed using Shimadzu Lab Solutions software. The column temperature was set to 60° for 3 minutes before ramping by 15°/min to 300° and then holding this temperature for 2 minutes.

Synthesis and Characterization

The methodologies for the synthesized compounds and palladium nanoparticle formation, and catalytic isomerization are shown below. $B(C_6F_5)_3$ and $Pd(cod)Cl_2$ were purchased from Strem.

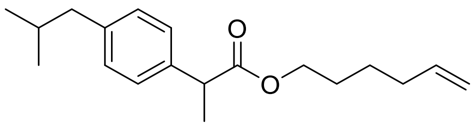

*Synthesis of hex-5-en-1-yl 2-(4-isobutylphenyl)propanoate (**1**).*

Ibuprofen (1.57 g, 7.63 mmol) was dissolved in $SOCl_2$ [3 mL] then a drop of DMF was added to the reaction. After the evolution of gas had diminished, the reaction vessel was immediately sealed with a septum held in place with electrical tape and the reaction was then stirred overnight. The reaction was then concentrated to remove excess $SOCl_2$, and the crude acid chloride was immediately dissolved in CH_2Cl_2 [5 mL] and added dropwise via syringe to a solution of hex-5-en-1-ol (0.83 mL, 6.93 mmol; ρ = 0.834 g/mL) and triethylamine (0.97 mL, 6.93 mmol; ρ = 0.726 g/mL) in CH_2Cl_2 [10 mL] that was cooled to 0 °C with an ice bath. The reaction was allowed to warm to room temperature, stirring overnight. The reaction was then washed with a saturated $NaHCO_3$ solution, water, and brine. The organic phase was then isolated and dried over $MgSO_4$, filtered, and concentrated via rotary evaporation. The crude material was then purified via column chromatography [silica; EtOAc/Hexanes (1:4)] to yield **1** as a clear oil (1.20 g, 60%). ^{1}H NMR ($CDCl_3$, 400 MHz): δ = 7.23-7.09 (4H, dd, J_1 = 46 Hz, J_2 = 8 Hz), 5.80-5.70 (1H, m), 5.01-4.94 (2H, m), 4.10-4.07 (2H, t, J = 6.5 Hz), 3.73-3.67 (1H, q, J = 7.1 Hz), 2.47-2.46 (3H, d, J = 7.2 Hz), 2.06-2.00 (2H, q, J = 7.6 Hz), 1.92-1.82 (1H, hep, J = 6.8 Hz Hz), 1.64-1.57 (2H, m), 1.52-1.50 (3H, d, J = 7.2 Hz), 1.41-1.33 (2H, p, 7.2 Hz), 0.92-0.91 (6H, d, 6.6 Hz). ^{13}C NMR ($CDCl_3$, 125 MHz): δ =174.7, 140.4, 138.3, 137.9, 129.2, 127.1, 114.7, 64.5, 45.2, 45.0, 33.1, 30.1, 27.9, 25.0, 22.3, 18.4. HRMS (ESI+) m/z: $[M+Na]^+$ calcd for $[C19H28O2Na]^+$ 311.19871, found 311.19687.

General Procedure for the Substrate Scope for Selective Isomerization of Terminal Olefins

$Pd(cod)Cl_2$ (3.2 mg, 0.0125 mmol) and $B(C_6F_5)_3$ (6.4 mg, 0.0125 mmol) were dissolved in CH_2Cl_2 [2.5 mL] within a 1-dram vial equipped with a septa cap. Et_3SiH (2 µL, 0.0125 mmol; ρ = 0.728 g/mL) was added via µL-syringe in one portion to the solution then mixed immediately, upon which the solution turns dark. Substrates (0.25 mmol) were then added via syringe to the sealed vial and the mixture was allowed to sit until isomerization of the terminal olefin was completed as determined by GC-MS analysis. The reaction was then filtered through a silica plug eluting with CH_2Cl_2 then concentrated via rotary evaporation. NMR analysis of the crude mixture was then performed to verify GC-MS results.

Procedure for Hg^0 Drop Test for Heterogeneity

$Pd(cod)Cl_2$ (3.2 mg, 0.0125 mmol) and $B(C_6F_5)_3$ (6.4 mg, 0.0125 mmol) were dissolved in CH_2Cl_2 [2.5 mL] within a 1-dram vial equipped with a septa cap. Et_3SiH (2 μL, 0.0125 mmol; ρ = 0.728 g/mL) was added via μL-syringe in one portion to the solution then mixed immediately, upon which the solution turns dark. A drop of Hg^0 was added to the reaction by syringe followed by 1-decene (47 μL, 0.25 mmol; ρ = 0.741 g/mL). The reaction was allowed to sit for 2 hours, then reaction was sampled for GC-MS analysis showing 10% isomerization of 1-decene with 100% mono-isomerization selectivity, and for 2-decenes a E/Z ratio = 1.0.

Procedure for Filtration Test for Heterogeneity

$Pd(cod)Cl_2$ (3.2 mg, 0.0125 mmol) and $B(C_6F_5)_3$ (6.4 mg, 0.0125 mmol) were dissolved in CH_2Cl_2 [2.5 mL] within a 1-dram vial equipped with a septa cap. Et_3SiH (2 μL, 0.0125mmol; ρ = 0.728 g/mL) was added via μL-syringe in one portion to the solution then mixed immediately, upon which the solution turns dark. The reaction was immediately filtered through a 0.2 μm PTFE syringe filter into a new 1 dram vial, then 1-decene (47 μL, 0.25 mmol; ρ = 0.741 g/mL) was added via syringe. The reaction was allowed to sit for 2 hours, then reaction was sampled for GC-MS analysis showing 34% isomerization of 1-decene with 88% mono-isomerization selectivity, and for 2-decenes a E/Z ratio = 1.0.

Procedure for [PdNp] Isolation Test for Heterogeneity

$Pd(cod)Cl_2$ (3.2 mg, 0.0125 mmol) and $B(C_6F_5)_3$ (6.4 mg, 0.0125 mmol) were dissolved in CH_2Cl_2 [2.5 mL] within a 1-dram vial equipped with a septa cap. Et_3SiH (2 μL, 0.0125 mmol; ρ = 0.728 g/mL) was added via μL-syringe in one portion to the solution then mixed immediately, upon which the solution turns dark. The solution was allowed to sit for 2 hours, upon which the Pd metal was precipitated from solution. The solution was decanted and the Pd metal was washed with CH_2Cl_2 [1 mL] followed by decanting three times. CH_2Cl_2 [2.5 mL] was added followed by 1-decene (47 μL, 0.25 mmol). The reaction was allowed to sit for 2 hours, then reaction was sampled for GC-MS analysis showing 46% isomerization of 1-decene with 95% mono-isomerization selectivity, and for 2-decenes a E/Z ratio = 1.3.

Procedure for the Large-Scale Selective Isomerization of 1-Decene

$Pd(cod)Cl_2$ (32 mg, 0.125 mmol) and $B(C_6F_5)_3$ (64 mg, 0.125 mmol) were dissolved in 1,2-dichloromethane [6.25 mL] within a 20 mL scintillation vial. Et_3SiH (10 μL, 0.125 mmol; ρ = 0.728 g/mL) was added via μL-syringe in one portion to the solution then mixed immediately, upon which the solution turns dark. 1-decene (0.24 mL, 1.25 mmol; 0.740 g/mL) was added via syringe and the solution was allowed to sit for 2 hours. The reaction was then filtered through a silica pad eluting hexanes then concentrated via rotary evaporation then vacuum distilled to yield

2-decene:decenes (91:9) as a clear liquid (0.22 mL, 91% recovery of material; 96% conversion of 1-decene to decenes; 91% 2-decene selectivity; E/Z = 1.8).

General Procedure for the Robustness Screening

$Pd(cod)Cl_2$ (3.2 mg, 0.0125 mmol) and $B(C_6F_5)_3$ (6.4 mg, 0.0125 mmol) were dissolved in CH_2Cl_2 [2.5 mL] within a 1-dram vial equipped with a septa cap. Et_3SiH (2 µL, 0.0125 mmol; ρ = 0.728 g/mL) was added via µL-syringe in one portion to the solution then mixed immediately, upon which the solution turns dark. An additive (0.25 mmol) followed by 1-decene (47 µL, 0.25 mmol) was then added to the reaction via syringe then allowed to sit for 2 hours. Mesitylene (35 µL, 0.25 mmol) was added via syringe, then the crude reaction was sampled for GC-MS analysis. Percent recovery of the additives was determined by comparing to a known standard solution of 0.25 mmol mesitylene and 0.25 mmol of the respective additive according to **Equation S1**. This robustness screening was done analogously to past work.[1]

$$\textbf{Equation S1: } \%\ \boldsymbol{Recovery} = \left(\frac{\boldsymbol{Area\ of\ Additive\ in\ Reaction} / \boldsymbol{Area\ of\ Mesitylene\ in\ Reaction}}{\boldsymbol{Area\ of\ Additive\ in\ Standard} / \boldsymbol{Area\ of\ Mesitylene\ in\ Standard}} \right) \boldsymbol{X\ 100}$$

GC-MS Traces for Condition Optimizations, Substrate Scope, and Robustness Screening

GC-MS Traces for Condition Screenings and Heterogeneity Tests

***Table S1**: Condition Screening and Optimizations*

Standard Conditions:

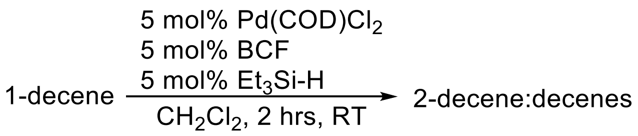

Substrate Concentration 0.2 M
Atmosphere N_2
Scale 0.25 mmol 1-decene

Entry	*Deviation from Standard Conditions*	*% Conversion*	*% m.i..*	*E/Z Ratio*
1	---	96	90	1.9
2	No Et_3Si-H	10	40	3.0
3	No BCF	---	---	---
4	2.5 mol% Pd(cod)Cl_2	92	90	1.7
5	HBpin instead of Et_3Si-H	39	92	1.3
6	DCE instead of CH_2Cl_2	98	88	1.8
7	ACN instead of CH_2Cl_2	---	---	---
8	THF instead of CH_2Cl_2	---	---	---
9	Toluene instead of CH_2Cl_2	4	61	1.4
10	Hexanes instead of CH_2Cl_2	24	75	1.6
11	Hg^0 Drop Test	10	100	1.0
12	Filtered post-reduction of Pd Test	34	88	1.0
13	Isolated Pd metal Test	46	95	1.3
14[a]	H_2 atmosphere instead of N_2	See pgs. S18-20		
15	Large Scale (1.25 mmol 1-decene)	96	91	1.8
16	0.5 mol% Pd(cod)Cl_2/BCF/Et_3Si-H	35	82	1.3
17	1.0 mol% Pd(cod)Cl_2/BCF/Et_3Si-H	54	89	1.2
18	2.5 mol% Pd(cod)Cl_2/BCF/Et_3Si-H	87	92	1.5
19	10 mol% Pd(cod)Cl_2/BCF/Et_3Si-H	93	92	1.6
20	20 mol% Pd(cod)Cl_2/BCF/Et_3Si-H	98	90	2.0
21	24 hrs	99	79	3.6
22	0.25 mmol additional 1-decene added at 1 hr, then reacted for 3 more hrs	See pgs. S29-S30		
23	10 mol% Et_3Si-H	87	89	1.9
24	20 mol% Et_3Si-H	32	89	1.4
25	No Et_3Si-H or BCF	---	---	---

% Conversion was determined with GC-MS by seeing how much 1-decene was converted to internal olefins. % Mono-isomerization selectivity (m.i.s.) was determined with GC-MS by seeing what percentage of the isomerized product was *E*-2-decene and *Z*-2-decene [a]Conditions led to the formation of decane and analysis of this data can be found on pages S18-S20

*Data for Table S1: Entry **1***

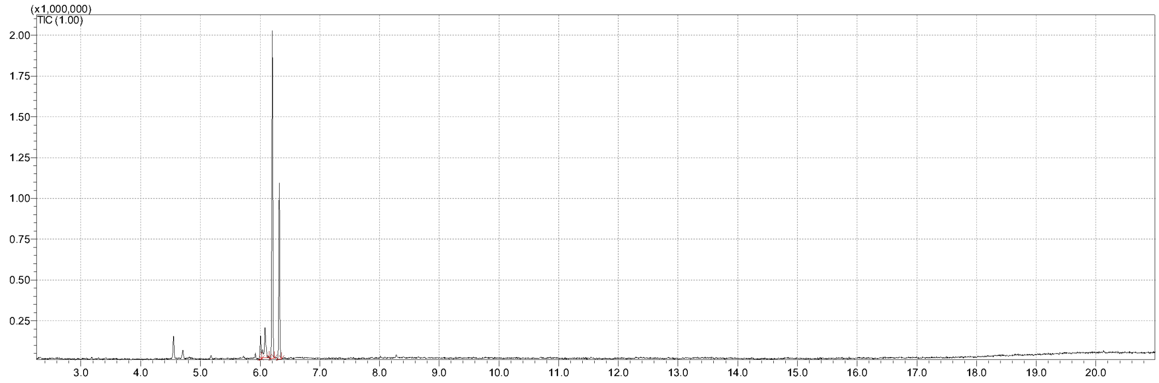

Figure S1. GC-MS Trace of Table S1: Entry 1; Standard conditions.

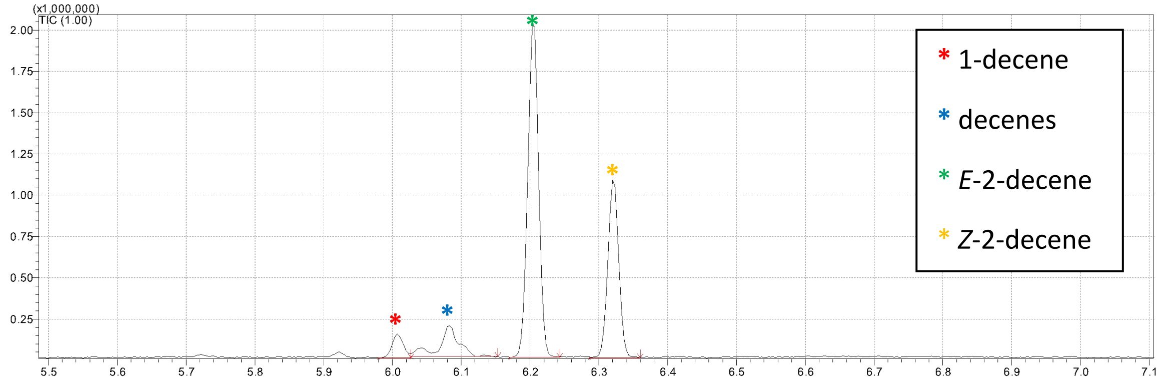

Figure S2. GC-MS Trace of Table S1: Entry 1; Standard conditions (Expanded View). Table below denoting peak identity, retention times, and areas of the peaks.

Table S2. Relative peak integrations for Table S1: entry 1

Compound	*RT*	*Start Time*	*End Time*	*Area*	*% Area*
1-decene	6.007	5.98	6.027	168951	4
decenes	6.082	6.027	6.153	363107	9
E-*2-decene*	6.205	6.17	6.243	2232179	56
Z-2-decene	6.321	6.287	6.36	1193802	31

*Data for Table S1: Entry **2***

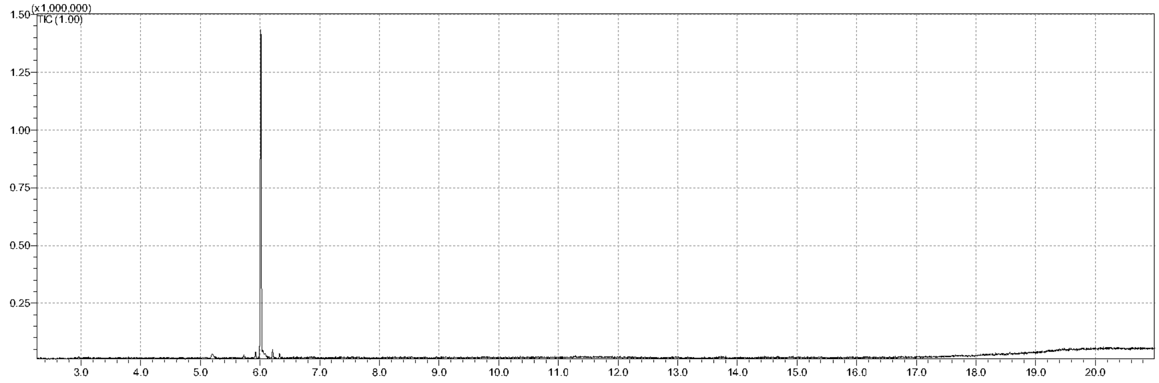

Figure S3. GC-MS Trace of Table S1: Entry 2; No Et_3SiH.

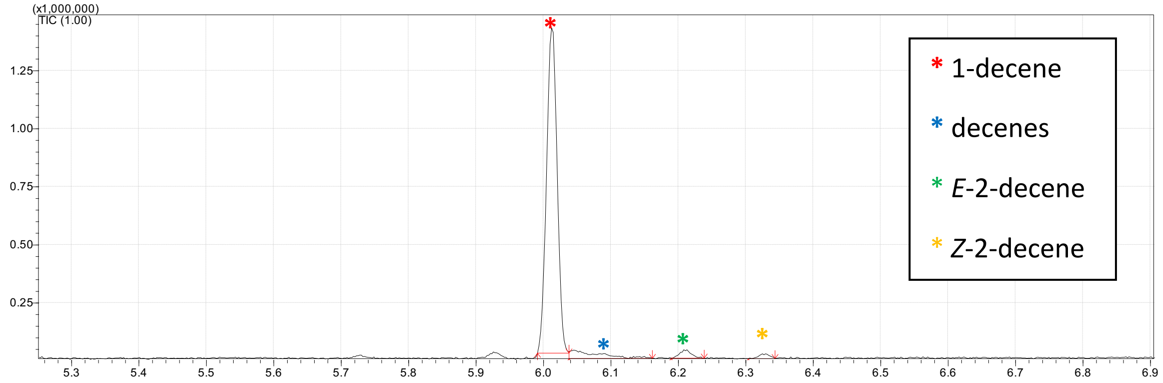

Figure S4. GC-MS Trace of Table S1: Entry 2; No Et_3SiH (Expanded View). Table below denoting peak identity, retention times, and areas of the peaks.

Table S3. Relative peak integrations for Table S1: entry 2

Compound	*RT*	*Start Time*	*End Time*	*Area*	*% Area*
1-decene	6.013	5.991	6.038	1534699	90
decenes	6.045	6.038	6.161	103849	6
E-*2-decene*	6.212	6.189	6.238	43285	3
Z-*2-decene*	6.33	6.304	6.343	24216	1

*Data for Table S1: Entry **3***

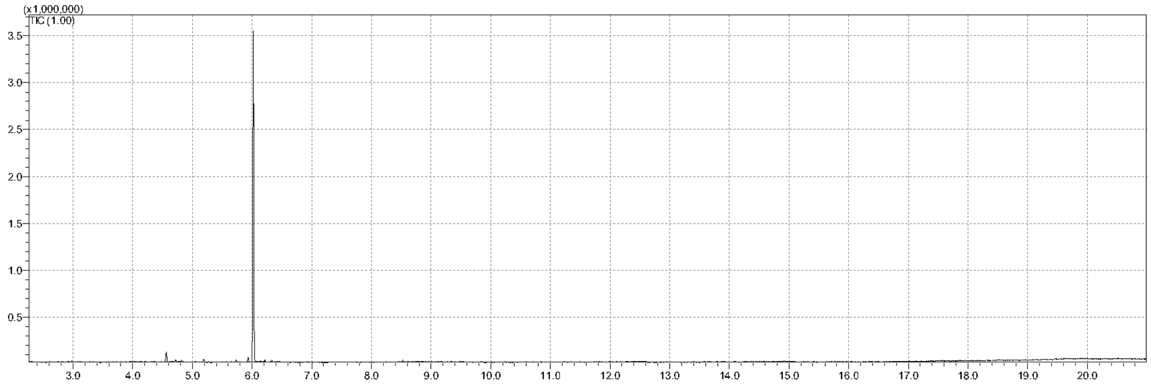

Figure S5. GC-MS Trace of Table S1: Entry 3; No BCF.

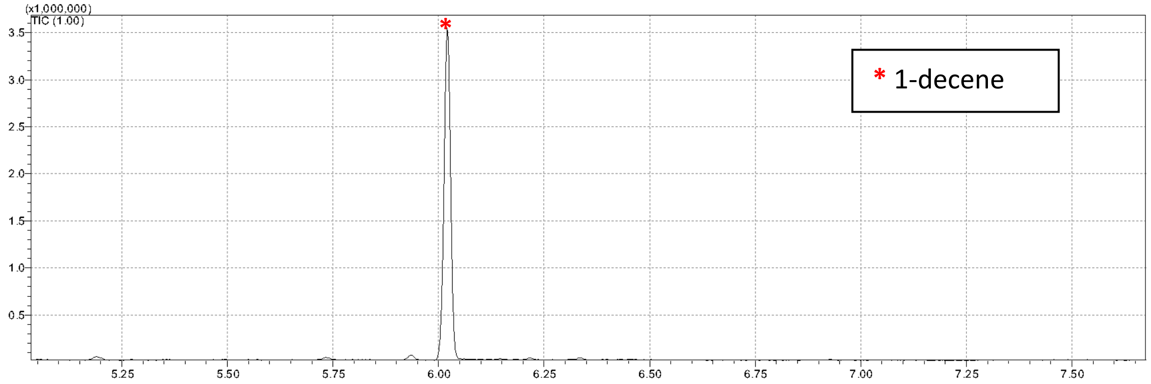

Figure S6. GC-MS Trace of Table S1: Entry 3; No BCF (Expanded View). Table below denoting peak identity, retention times, and areas of the peaks.

Table S4. Relative peak integrations for Table S1: entry 3

Compound	*RT*	*Start Time*	*End Time*	*Area*	*% Area*
1-decene	6.021	5.987	6.1	3864815	100

*Data for Table S1: Entry **4***

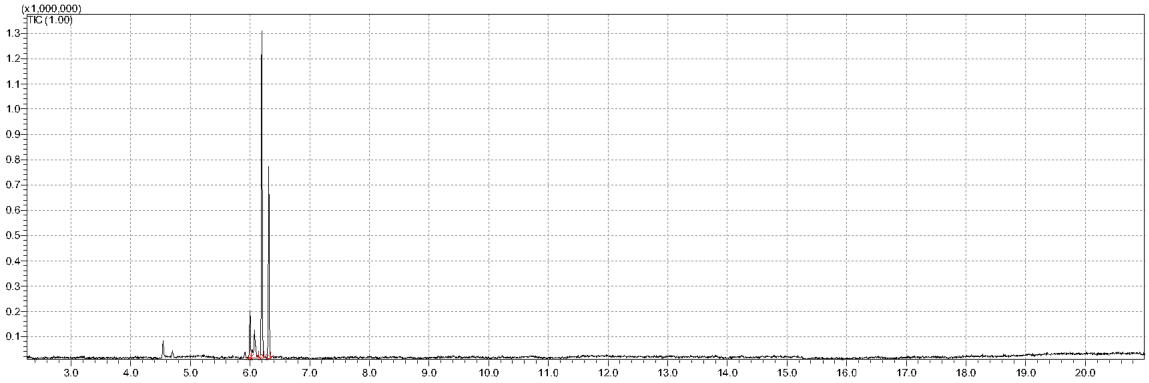

Figure S7. GC-MS Trace of Table S1: Entry 4; 2.5 mol% Pd(cod)Cl_2.

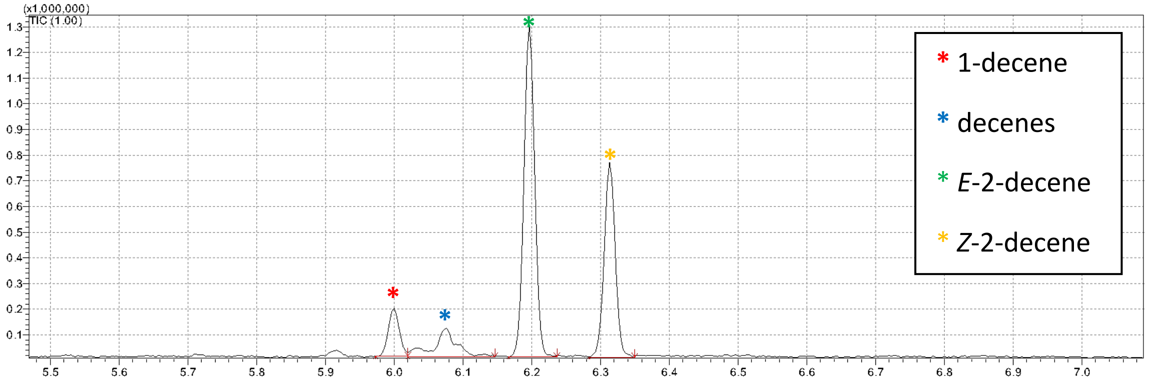

Figure S8. GC-MS Trace of Table S1: Entry 4; 2.5 mol% Pd(cod)Cl (Expanded View). Table below denoting peak identity, retention times, and areas of the peaks.

Table S5. Relative peak integrations for Table S1: entry 4

Compound	*RT*	*Start Time*	*End Time*	*Area*	*% Area*
1-decene	6	5.973	6.02	206354	8
decenes	6.075	6.02	6.147	249784	9
E-*2-decene*	6.197	6.167	6.237	1408596	52
Z-*2-decene*	6.314	6.283	6.35	847138	31

*Data for Table S1: Entry **5***

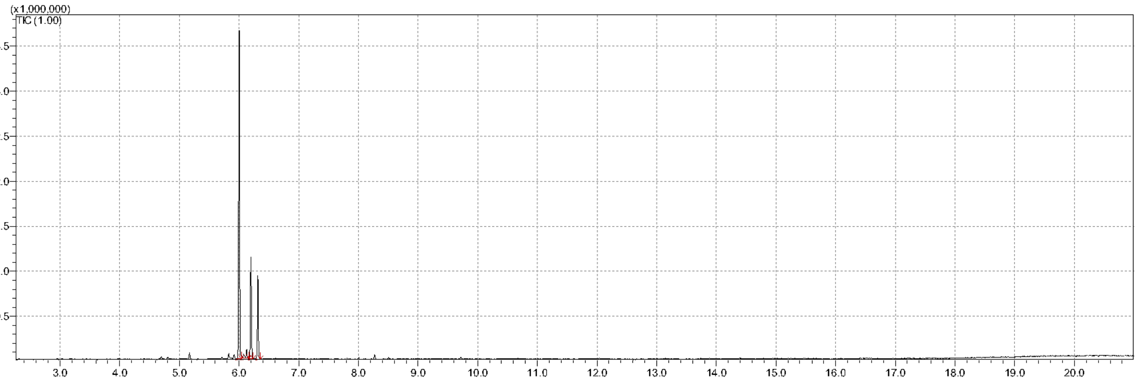

Figure S9. GC-MS Trace of Table S1: Entry 5; HBpin instead of Et_3Si-H.

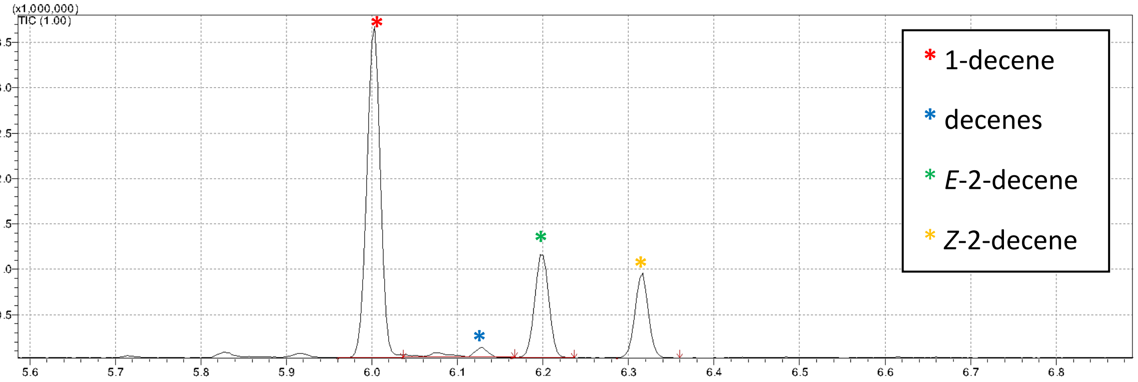

Figure S10. GC-MS Trace of Table S1: Entry 5; ; HBpin instead of Et_3Si-H (Expanded View). Table below denoting peak identity, retention times, and areas of the peaks.

Table S6. Relative peak integrations for Table S1: entry 5

Compound	*RT*	*Start Time*	*End Time*	*Area*	*% Area*
1-decene	6.002	5.96	6.037	4019896	61
decenes	6.128	6.037	6.167	189810	3
E-*2-decene*	6.198	6.163	6.237	1322013	20
Z-*2-decene*	6.316	6.287	6.36	1015535	16

*Data for Table S1: Entry **6***

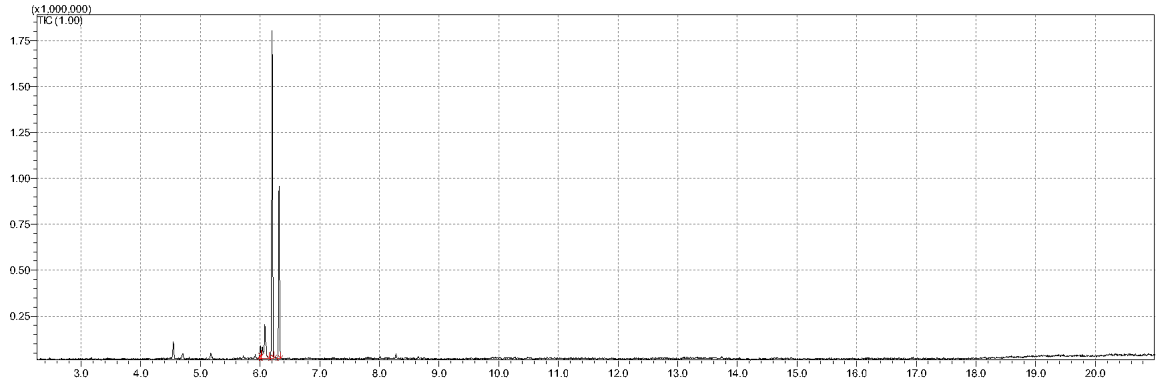

Figure S11. GC-MS Trace of Table S1: Entry 6; DCE instead of CH_2Cl_2.

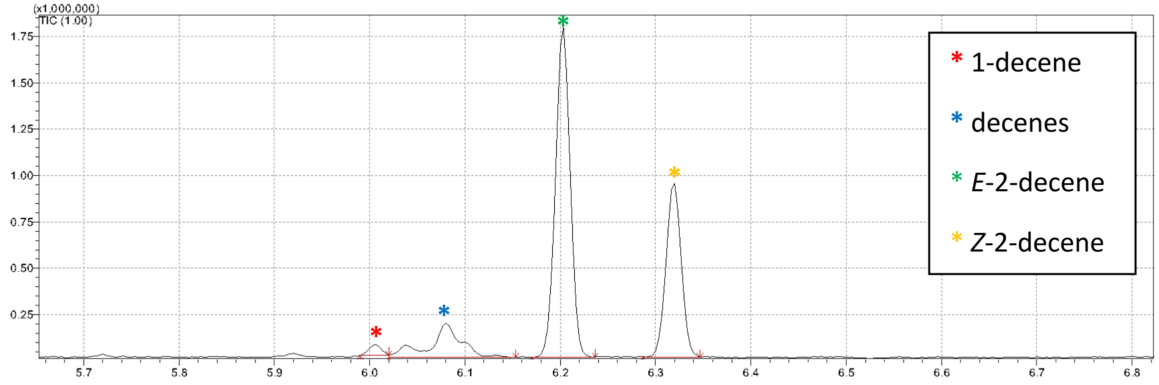

Figure S12. GC-MS Trace of Table S1: Entry 6; DCE instead of CH_2Cl_2 (Expanded View). Table below denoting peak identity, retention times, and areas of the peaks.

Table S7. Relative peak integrations for Table S1: entry 6

Compound	*RT*	*Start Time*	*End Time*	*Area*	*% Area*
1-decene	6.006	5.99	6.02	51961	2
decenes	6.08	6.02	6.153	407345	12
E-*2-decene*	6.203	6.17	6.237	1933340	56
Z-*2-decene*	6.319	6.287	6.347	1053818	31

Data for Table S1: Entry 7

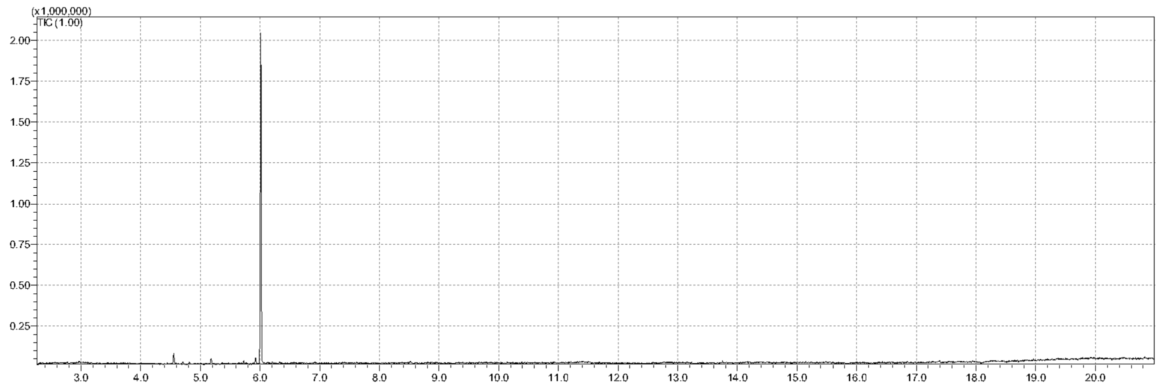

Figure S13. GC-MS Trace of Table S1: Entry 7; acetonitrile (ACN) instead of CH_2Cl_2. No isomerization occurred.

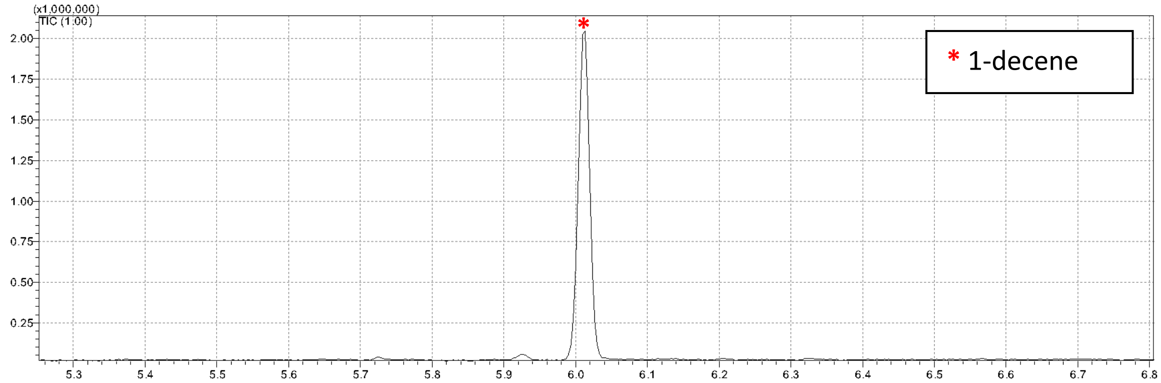

Figure S14. GC-MS Trace of Table S1: Entry 7; acetonitrile (ACN) instead of CH_2Cl_2. No isomerization occurred (Expanded View). Table below denoting peak identity, retention times, and areas of the peaks.

Table S8. Relative peak integrations for Table S1: entry 7

Compound	*RT*	*Start Time*	*End Time*	*Area*	*% Area*
1-decene	6.012	5.977	6.053	2282215	100

*Data for Table S1: Entry **8***

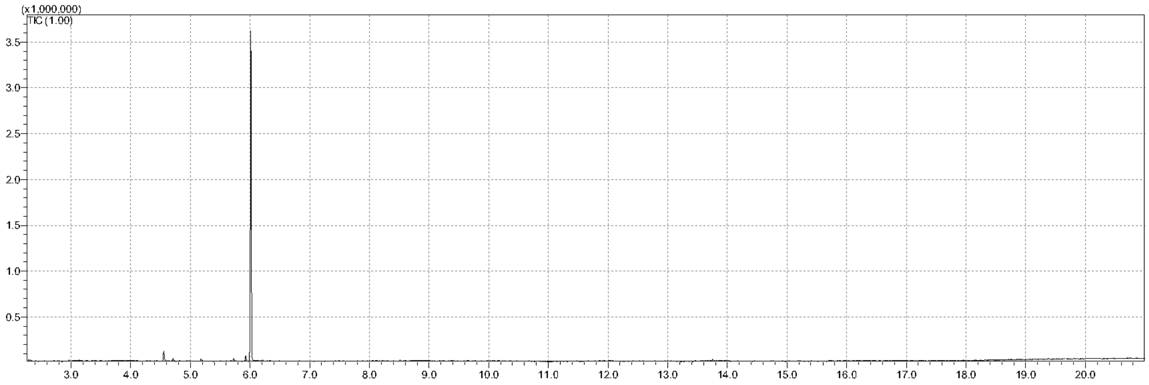

Figure S15. GC-MS Trace of Table S1: Entry 8; tetrahydrofuran (THF) instead of CH_2Cl_2. No isomerization occurred.

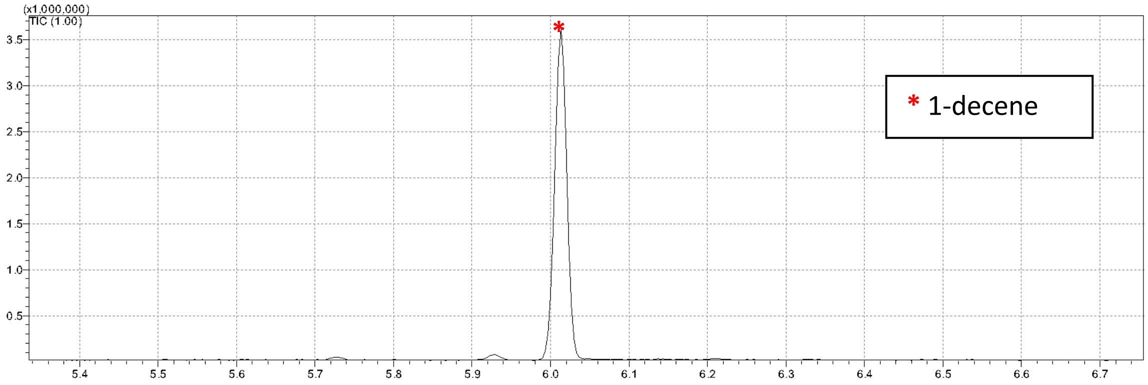

Figure S16. GC-MS Trace of Table S1: Entry 8; tetrahydrofuran (THF) instead of CH_2Cl_2. No isomerization occurred (Expanded View). Table below denoting peak identity, retention times, and areas of the peaks.

Table S9. Relative peak integrations for Table S1: entry 8

Compound	*RT*	*Start Time*	*End Time*	*Area*	*% Area*
1-decene	6.013	5.98	6.05	3907311	100

*Data for Table S1: Entry **9***

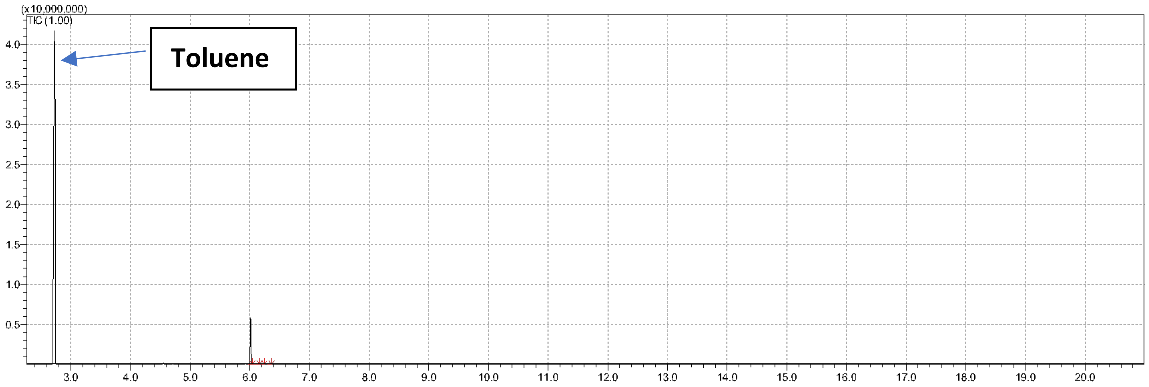

Figure S17. GC-MS Trace of Table S1: Entry 9; toluene instead of CH_2Cl_2. No isomerization occurred.

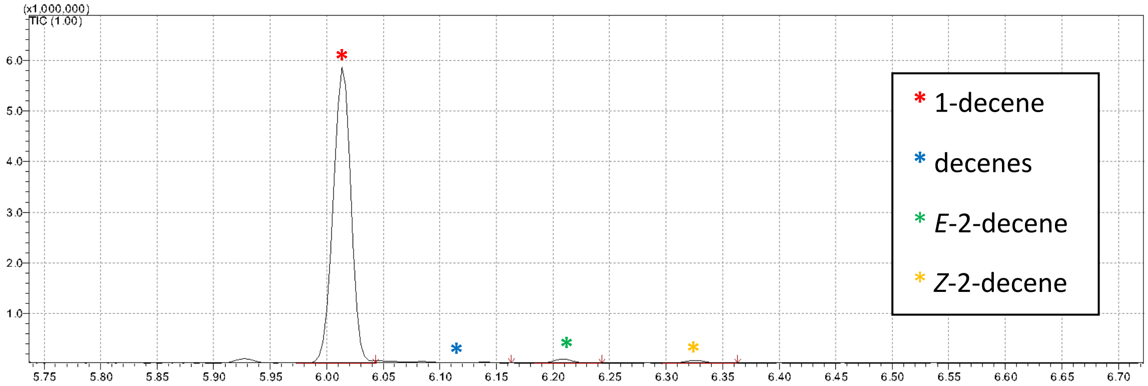

Figure S18. GC-MS Trace of Table S1: Entry 9; toluene instead of CH_2Cl_2 (Expanded View). Table below denoting peak identity, retention times, and areas of the peaks.

Table S10. Relative peak integrations for Table S1: entry 9

Compound	*RT*	*Start Time*	*End Time*	*Area*	*% Area*
1-decene	6.014	5.973	6.043	6374912	96
decenes	6.045	6.043	6.163	102986	2
E-*2-decene*	6.21	6.183	6.243	95174	1
Z-*2-decene*	6.327	6.297	6.363	66283	1

*Data for Table S1: Entry **10***

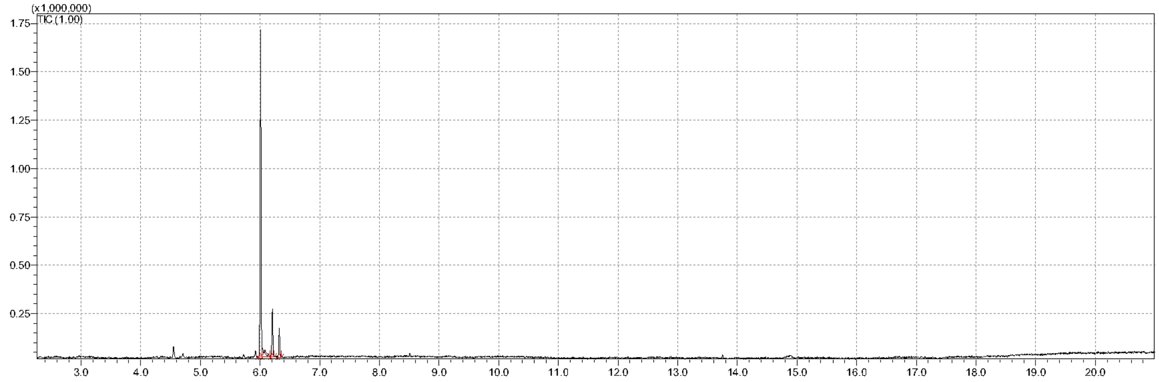

Figure S19. GC-MS Trace of Table S1: Entry 10; hexanes instead of CH_2Cl_2.

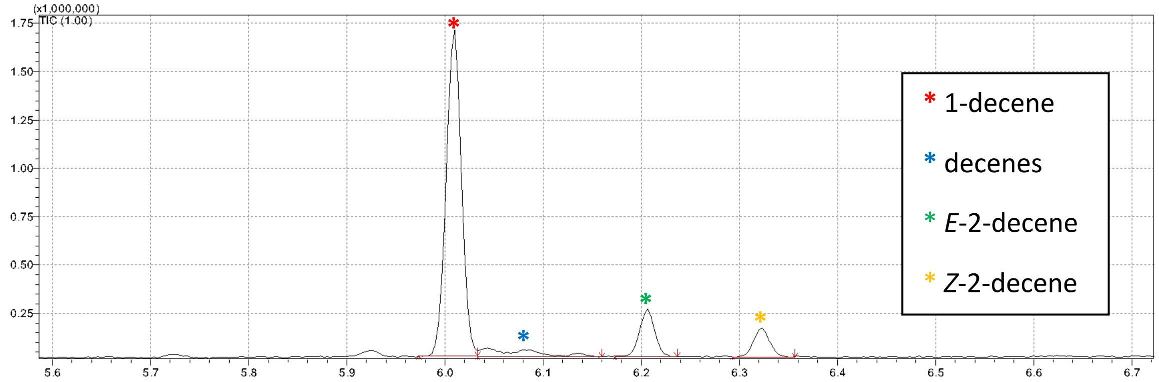

Figure S20. GC-MS Trace of Table S1: Entry 10; hexanes instead of CH_2Cl_2 (Expanded View). Table below denoting peak identity, retention times, and areas of the peaks.

Table S11. Relative peak integrations for Table S1: entry 10

Compound	*RT*	*Start Time*	*End Time*	*Area*	*% Area*
1-decene	6.009	5.973	6.033	1830588	76
decenes	6.043	6.033	6.16	147727	6
E-*2-decene*	6.206	6.173	6.237	268506	11
Z-*2-decene*	6.323	6.293	6.357	169983	7

*Data for Table S1: Entry **11***

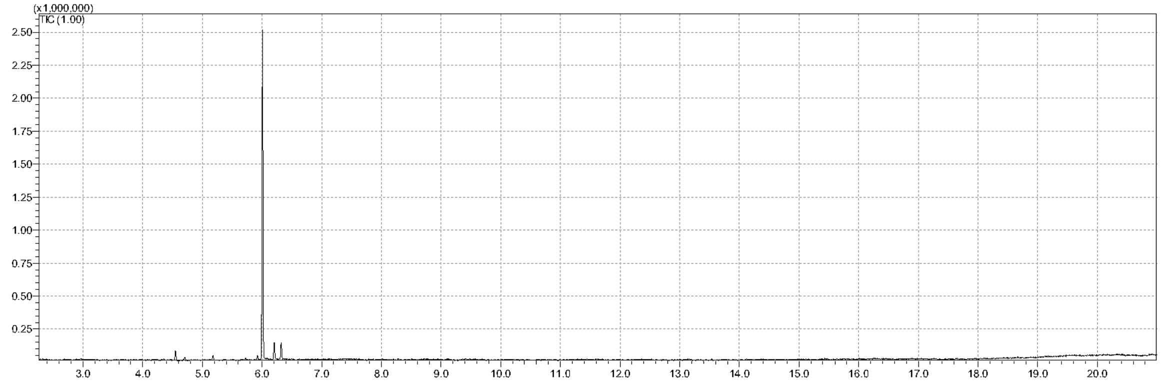

Figure S21. GC-MS Trace of Table S1: Entry 11; Hg^0 drop test for heterogeneity.

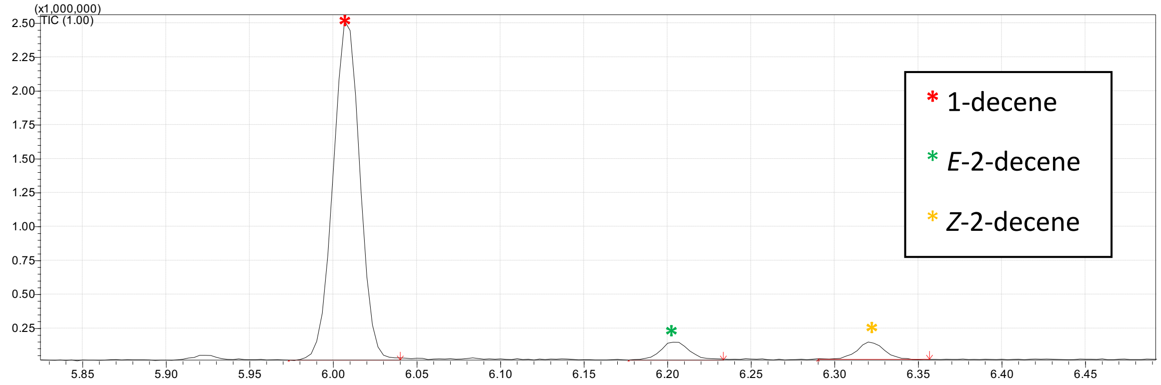

Figure S22. GC-MS Trace of Table S1: Entry 11; Hg^0 drop test for heterogeneity (Expanded View). Table below denoting peak identity, retention times, and areas of the peaks.

Table S12. Relative peak integrations for Table S1: entry 11

Compound	*RT*	*Start Time*	*End Time*	*Area*	*% Area*
1-decene	6.008	5.973	6.04	2796305	90
E-*2-decene*	6.204	6.177	6.233	168275	5
Z-*2-decene*	6.321	6.29	6.357	146419	5

*Data for Table S1: Entry **12***

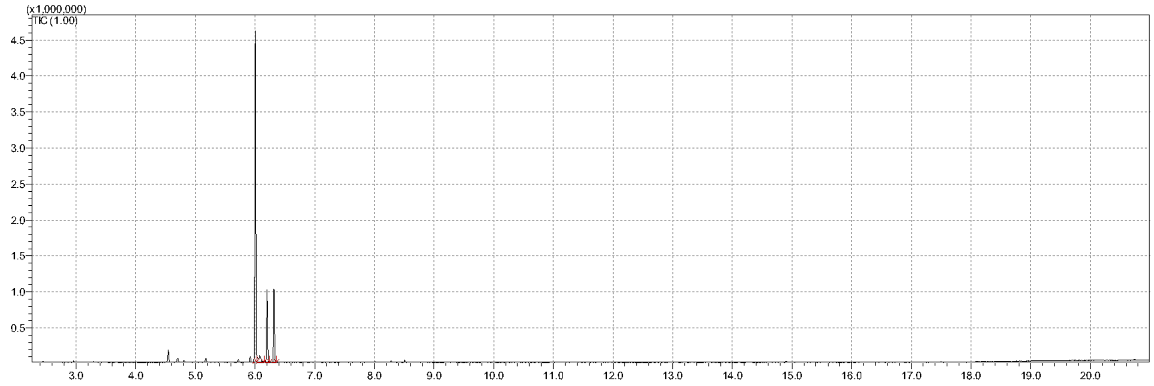

Figure S23. GC-MS Trace of Table S1: Entry 12; Filtered test for heterogeneity.

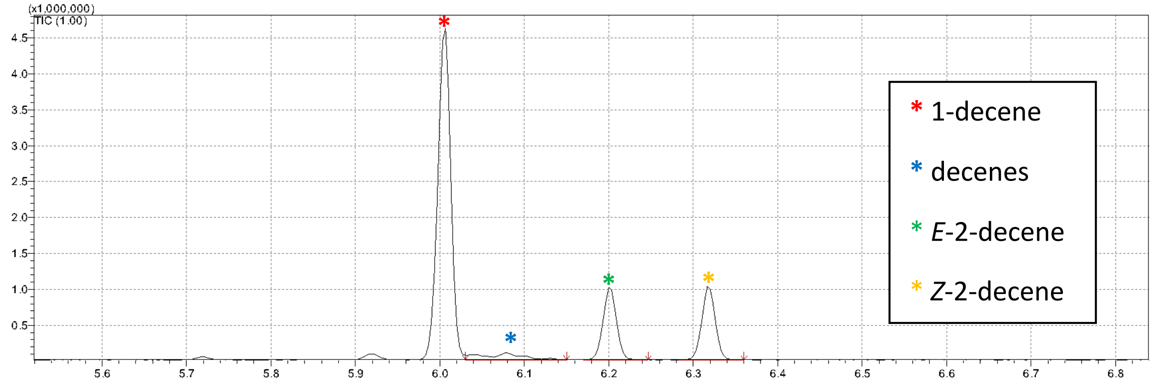

Figure S24. GC-MS Trace of Table S1: Entry 12; Filtered test for heterogeneity (Expanded View). Table below denoting peak identity, retention times, and areas of the peaks.

Table S13. Relative peak integrations for Table S1: entry 12

Compound	*RT*	*Start Time*	*End Time*	*Area*	*% Area*
1-decene	6.005	5.977	6.03	4953245	66
decenes	6.078	6.03	6.15	303977	4
E-*2-decene*	6.201	6.17	6.247	1114027	15
Z-2-decene	6.318	6.283	6.36	1142000	15

*Data for Table S1: Entry **13***

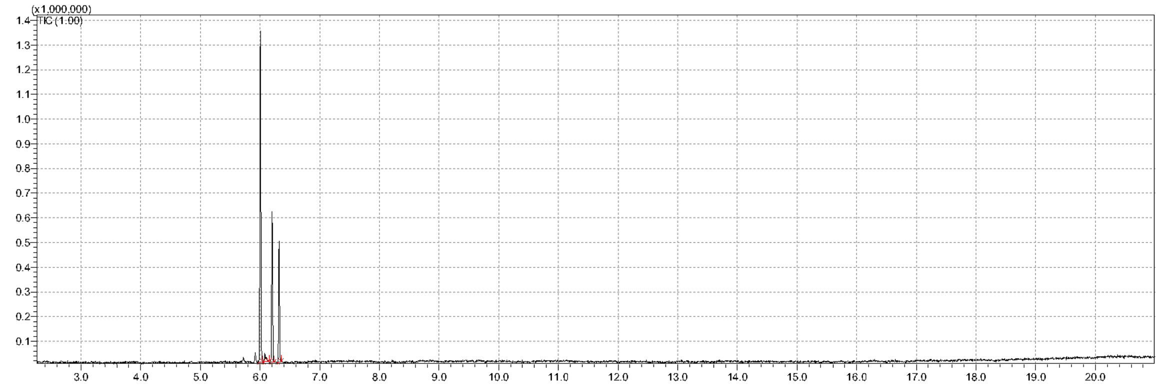

Figure S25. GC-MS Trace of Table S1: Entry 13; Isolated Pd metal Test.

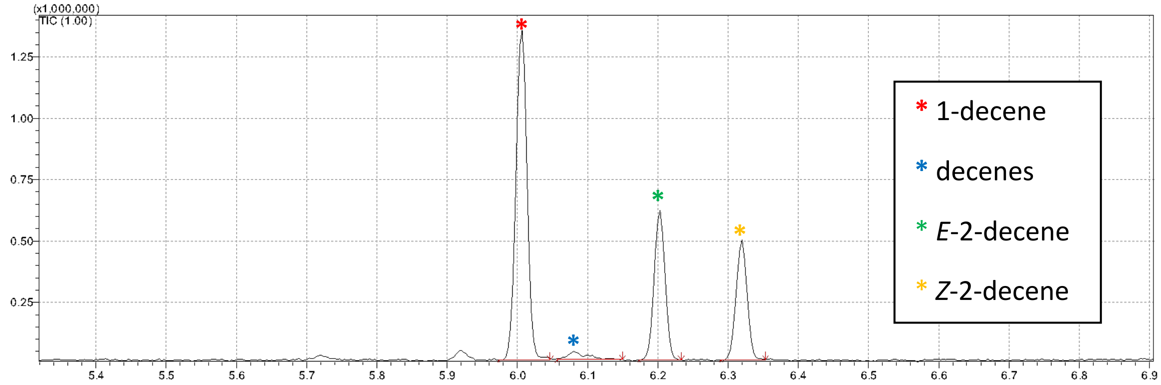

Figure S26. GC-MS Trace of Table S1: Entry 13; Isolated Pd metal Test (Expanded View). Table below denoting peak identity, retention times, and areas of the peaks.

Table S14. Relative peak integrations for Table S1: entry 13

Compound	*RT*	*Start Time*	*End Time*	*Area*	*% Area*
1-decene	6.006	5.973	6.047	1500240	54
decenes	6.08	6.057	6.15	59915	2
E-*2-decene*	6.203	6.173	6.233	667603	24
Z-*2-decene*	6.32	6.29	6.353	527306	19

*Data for Table S1: Entry **14***

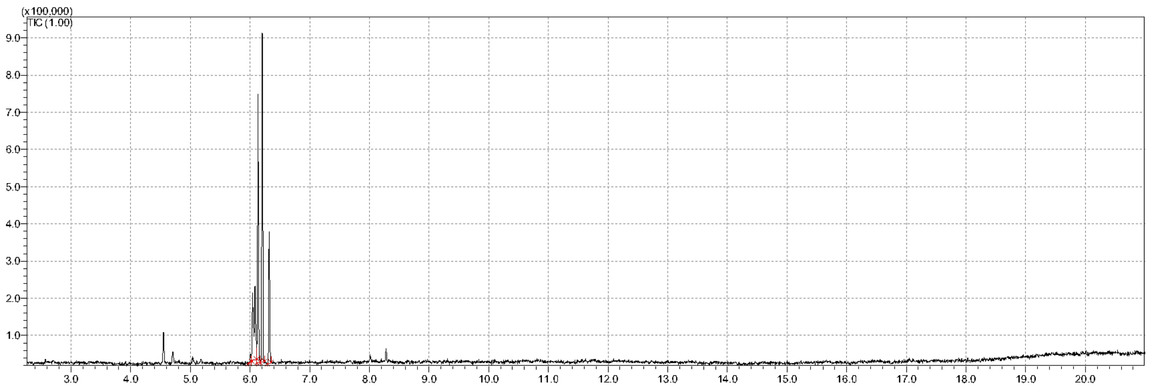

Figure S27. GC-MS Trace of Table S1: Entry 14; H_2 atmosphere instead of N_2.

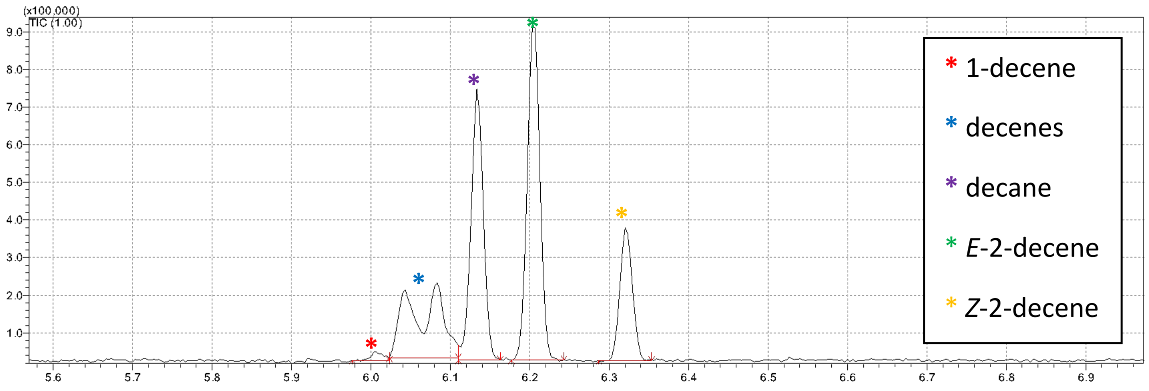

Figure S28. GC-MS Trace of Table S1: Entry 14; H_2 atmosphere instead of N_2. Table below denoting peak identity, retention times, and areas of the peaks.

Table S15. Relative peak integrations for Table S1: entry 14

Compound	*RT*	*Start Time*	*End Time*	*Area*	*% Area*
1-decene	6.005	5.977	6.023	23804	1
decenes	6.083	6.023	6.11	549875	20
decane	6.134	6.11	6.163	775268	28
E-*2-decene*	6.205	6.177	6.243	992706	36
Z-*2-decene*	6.321	6.287	6.353	390450	14

*Table S1: Entry **14**; H_2 Activity for Isomerization Selectivity*

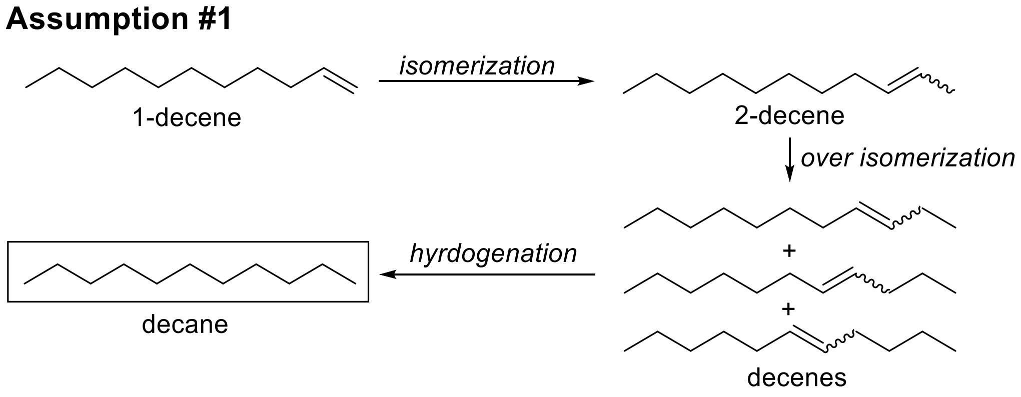

Scheme S1. Isomerization and hydrogenation under H_2 atmosphere assumption 1.

From the H_2 atmosphere experiment, we can make a few assumptions to determine the upper and lower limits for the mono-isomerization selectivity of this reaction. The 1st assumption is that only decenes were hydrogenated. In this instance we assume that the decane was over isomerized to decenes prior to hydrogenation giving us Equation S2 to determine % m.i.s.(decenes):

Equation S2: $$\%m.i.s.(decenes) = \frac{(Area\ of\ 2{-}Decenes)}{(Area\ of\ 2{-}Decenes) + (Area\ of\ Decenes) + (Area\ of\ Decane)}$$

$$\%m.i.s.(decenes) = \frac{(992706 + 390450)}{(992706 + 390450) + (549875) + (775268)} = \mathbf{51}\%$$

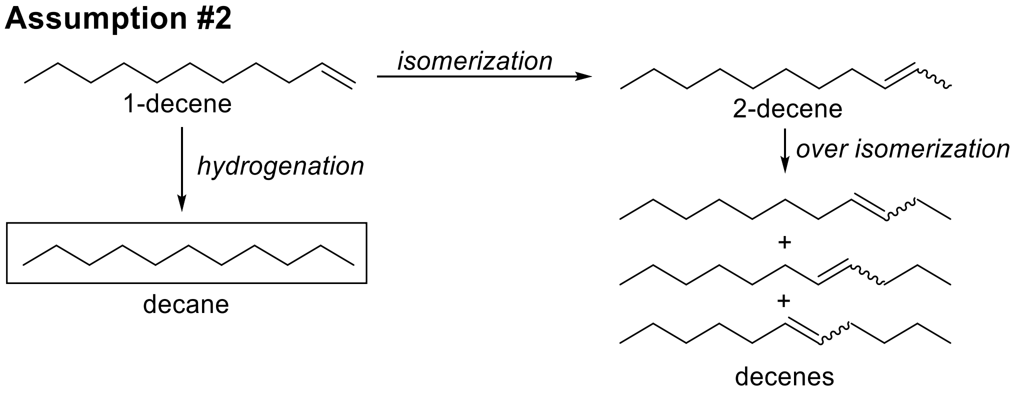

Scheme S2. Isomerization and hydrogenation under H_2 atmosphere assumption 2.

Now, the 2nd assumption that can be made is that only 1-decene was hydrogenated. In this instance the isomerization chemistries selectivity can be determined by just analyzing the relative amounts of isomerized olefin products, giving us Equation S3 to determine % m.i.s.(1-decene):

Equation S3: $\%M.I.S.(1-decene) = \frac{(Area\ of\ 2\text{–}Decenes)}{(Area\ of\ 2\text{–}Decenes) + (Area\ of\ Decenes)}$

$$\%M.I.S.(1-decene) = \frac{(992706\ +\ 390450)}{(549875) + (775268) + (549875)} = \mathbf{56}\%$$

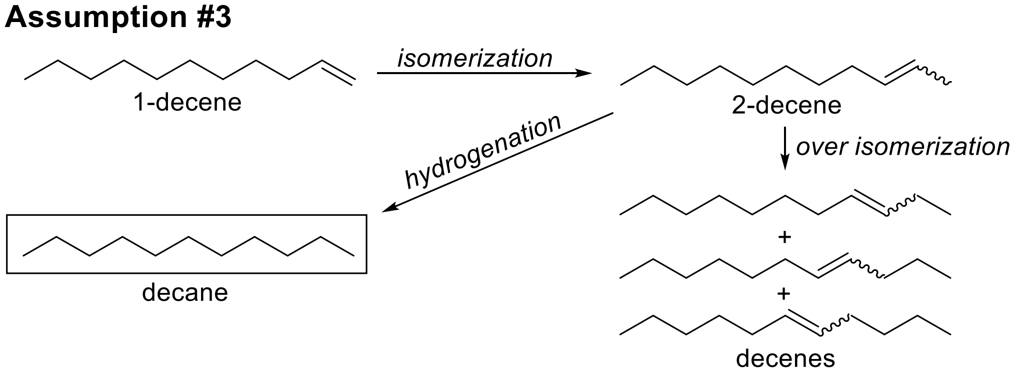

Scheme S3. Isomerization and hydrogenation under H_2 atmosphere assumption 3.

Finally, the 3rd assumption that can be made is that only 2-decene was hydrogenated. In this instance we assume that all the decane was isomerized to 2-decenes prior to hydrogenation which then gives us this Equation S4 for % m.i.s. (2-decenes):

Equation S4: $\%m.i.s.(2-decenes) = \frac{(Area\ of\ 2\text{–}Decenes) + (Area\ of\ Decane)}{(Area\ of\ 2\text{–}Decenes) + (Area\ of\ Decane) + (Area\ of\ Decenes)}$

$$\%m.i.s.(2-decenes) = \frac{(992706\ +\ 390450) + (775268)}{(992706\ +\ 390450)\ +\ (549875)\ +\ (775268)} = \mathbf{80}\%$$

In reality, we cannot assume only one of these processes are occurring but are all occurring in varying amounts at the same time. Assumption #3 enforces mono-isomerization selectivity by removing the possibility for further 2-decene isomerization to occur upon hydrogenation to decane therefore we state that the maximum %m.i.s.(2-decene) = 80%. Analysis of assumption #1 shows a substantial drop in 2-decene selectivity giving a minimum %m.i.s.(decenes) = 51%. Finally, given the higher prevalence of 1-decene at the start of the reaction, assumption #2 is likely more prevalent at the start of the reaction and gives a %m.i.s.(1-decene) = 56%. A possibility is that upon hydrogenation of 1-decene to decanes, the remaining olefins are effectively over-isomerized due to an effective increase in catalytic loading. However, when analyzing reactions where the catalytic loading was increased up to 20 mol% Pd(cod)Cl_2/BCF/Et_3Si-H (Table S1; Entry 20) there is no loss of mono-isomerization selectivity with increased catalytic loading. We can therefore disregard this possibility meaning that even if the effective catalytic loading is increasing due to

1-decene hydrogenation that is not the reason for the loss in mono-isomerization selectivity when an H_2 atmosphere is introduced to these isomerization conditions.

A crude average of these mono-isomerization selectivity values gives %m.i.s.$_{avg}$ = 62%. Therefore, from this analysis we conclude that the H_2 formed from Et_3Si-H reduction of Pd(cod)Cl_2 is absorbed on the surface of the reduced Pd nanoparticles and is active in the isomerization chemistry in this system. When the atmosphere in H_2, the reactivity of the system increases due to increasing amounts of absorbed H_2 on the Pd nanoparticle surfaces which decreases mono-isomerization selectivity relative to when the atmosphere is N_2 gas (%m.i.s. = 90% for standard conditions).

*Data for Table S1: Entry **15***

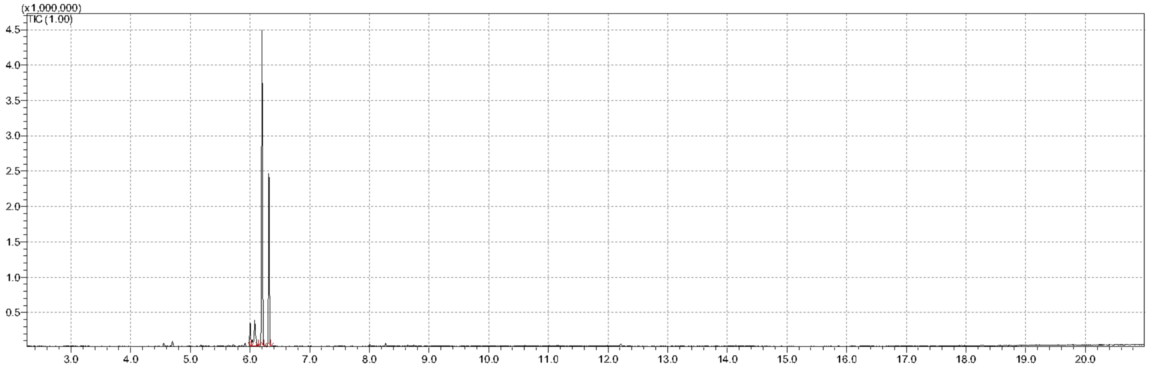

Figure S29. GC-MS Trace of Table S1: Entry 15; Large Scale of 1.25 mmol.

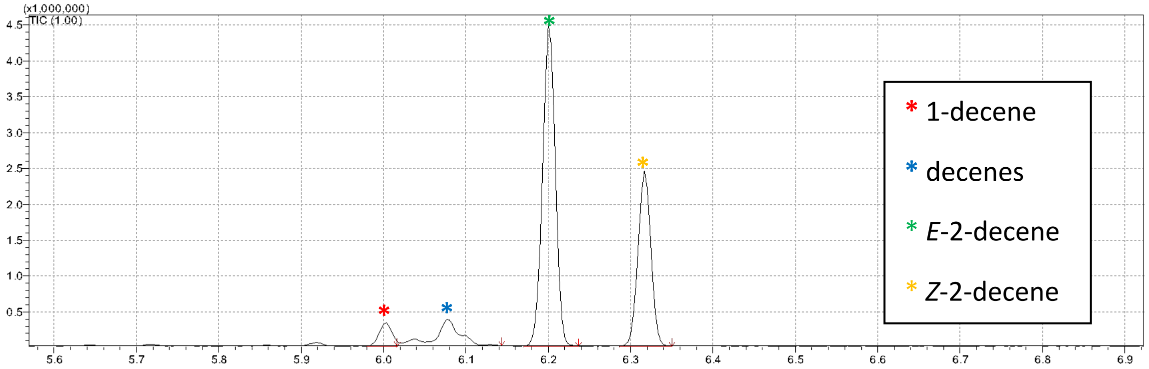

Figure S30. GC-MS Trace of Table S1: Entry 15; Large Scale of 1.25 mmol (Expanded View). Table below denoting peak identity, retention times, and areas of the peaks.

Table S16. Relative peak integrations for Table S1: entry 15

Compound	*RT*	*Start Time*	*End Time*	*Area*	*% Area*
1-decene	6.003	5.98	6.017	359687	4
decenes	6.078	6.017	6.143	732357	9

E-2-decene	6.201	6.17	6.237	4844773	56
Z-2-decene	6.317	6.287	6.35	2667148	31

Data for Table S1: Entry ***16***

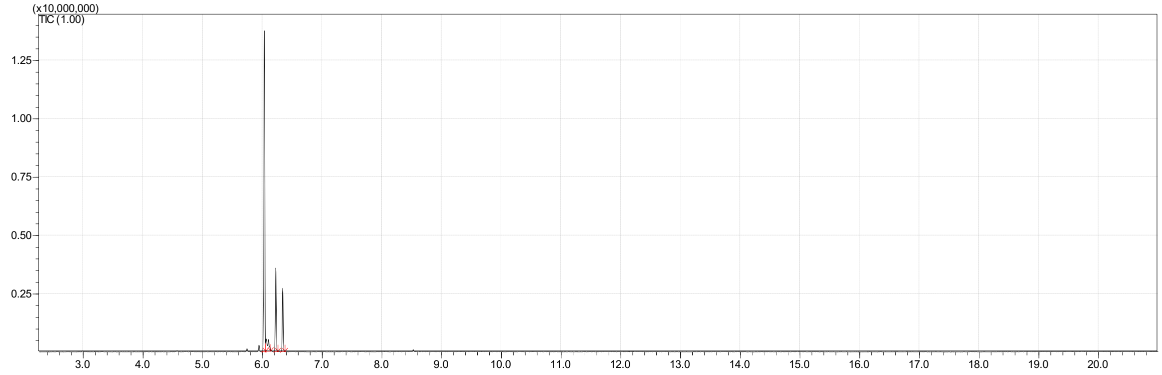

Figure S31. GC-MS Trace of Table S1: Entry 16; 0.5 mol% Pd(cod)Cl_2/BCF/Et_3Si-H.

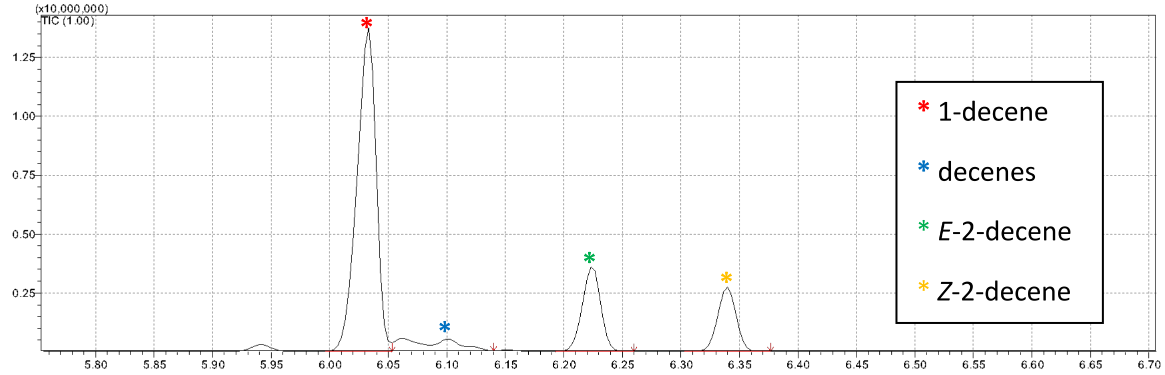

Figure S32. GC-MS Trace of Table S1: Entry 16; 0.5 mol% Pd(cod)Cl_2/BCF/Et_3Si-H (Expanded View). Table below denoting peak identity, retention times, and areas of the peaks.

Table S17. Relative peak integrations for Table S1: entry 16

Compound	*RT*	*Start Time*	*End Time*	*Area*	*% Area*
1-decene	6.033	5.997	6.053	15675919	65
decenes	6.062	6.053	6.14	1477142	6
E-2-decene	6.224	6.193	6.26	3920449	16
Z-2-decene	6.339	6.303	6.377	2960366	12

*Data for Table S1: Entry **17***

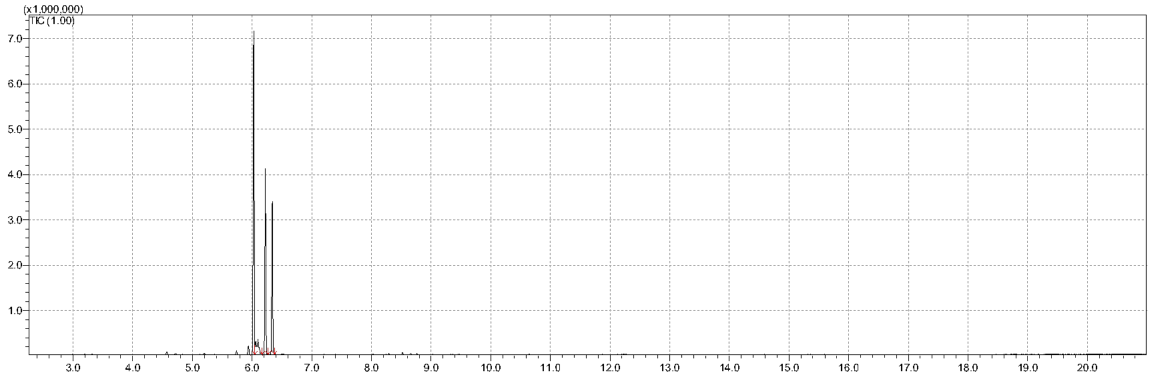

Figure S33. GC-MS Trace of Table S1: Entry 17; 1.0 mol% Pd(cod)Cl_2/BCF/Et_3Si-H.

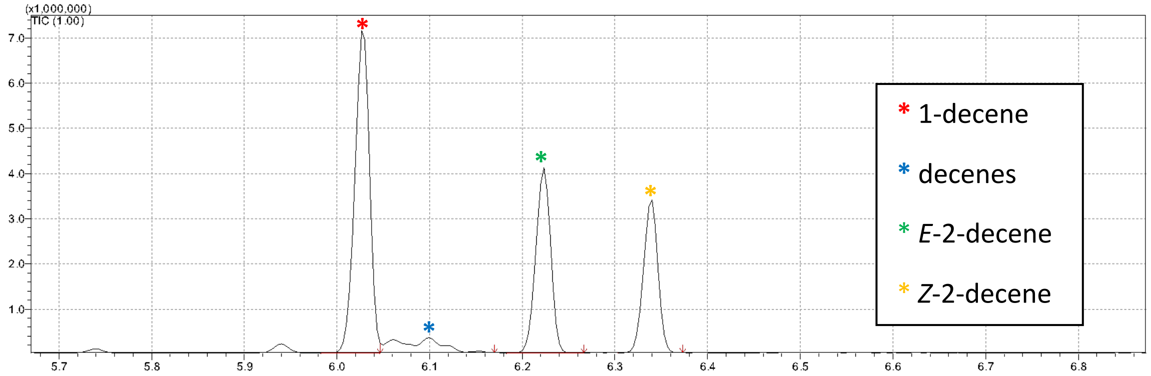

Figure S34. GC-MS Trace of Table S1: Entry 17; 1.0 mol% Pd(cod)Cl_2/BCF/Et_3Si-H (Expanded View). Table below denoting peak identity, retention times, and areas of the peaks.

Table S18. Relative peak integrations for Table S1: entry 17

Compound	*RT*	*Start Time*	*End Time*	*Area*	*% Area*
1-decene	6.028	5.983	6.047	7955583	46
decenes	6.099	6.047	6.17	1070356	6
E-*2-decene*	6.223	6.183	6.267	4579732	26
Z-*2-decene*	6.339	6.303	6.373	3684043	21

*Data for Table S1: Entry **18***

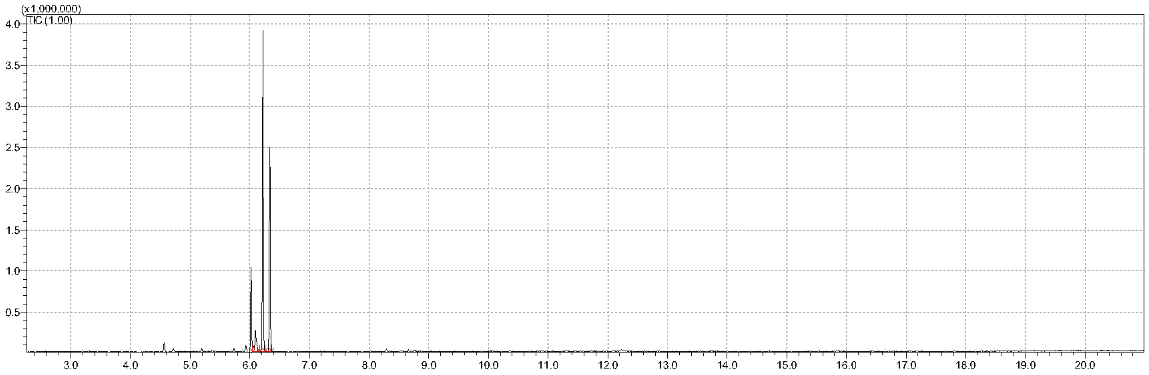

Figure S35. GC-MS Trace of Table S1: Entry 18; 2.5 mol% Pd(cod)Cl_2/BCF/Et_3Si-H.

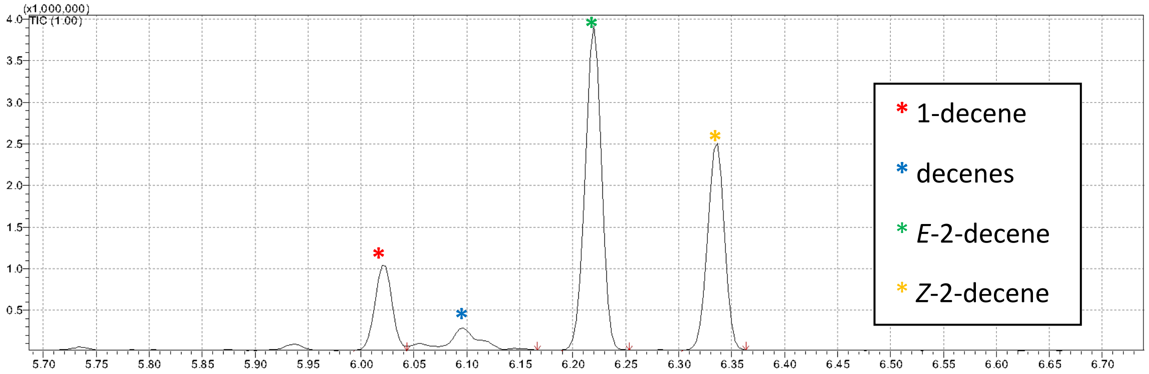

Figure S36. GC-MS Trace of Table S1: Entry 18; 2.5 mol% Pd(cod)Cl_2/BCF/Et_3Si-H (Expanded View). Table below denoting peak identity, retention times, and areas of the peaks.

Table S19. Relative peak integrations for Table S1: entry 18

Compound	*RT*	*Start Time*	*End Time*	*Area*	*% Area*
1-decene	6.021	5.993	6.043	1172555	13
decenes	6.096	6.043	6.167	583230	7
E-*2-decene*	6.22	6.19	6.253	4228173	48
Z-*2-decene*	6.335	6.303	6.363	2776752	32

*Data for Table S1: Entry **19***

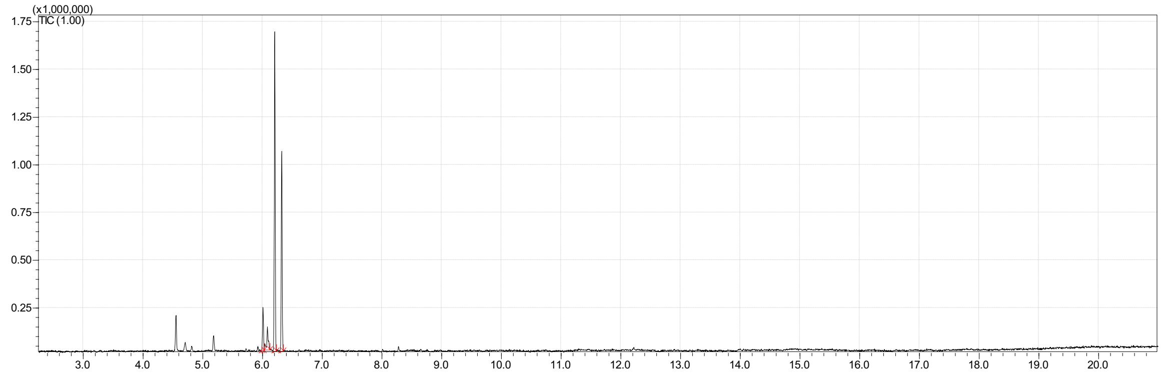

Figure S37. GC-MS Trace of Table S1: Entry 19; 10 mol% Pd(cod)Cl_2/BCF/Et_3Si-H.

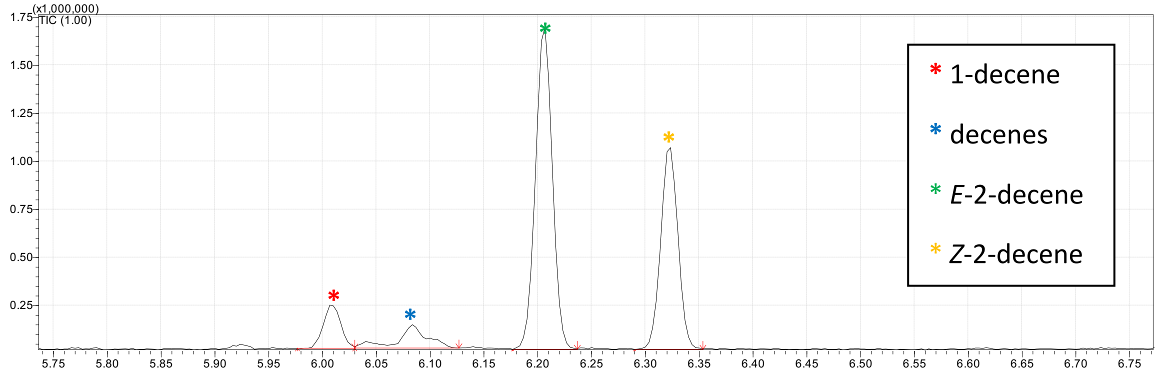

Figure S38. GC-MS Trace of Table S1: Entry 19; 10 mol% Pd(cod)Cl_2/BCF/Et_3Si-H (Expanded View). Table below denoting peak identity, retention times, and areas of the peaks.

Table S20. Relative peak integrations for Table S1: entry 19

Compound	*RT*	*Start Time*	*End Time*	*Area*	*% Area*
1-decene	6.008	5.977	6.03	255294	7
decenes	6.083	6.03	6.127	222535	6
E-*2-decene*	6.206	6.177	6.237	1835850	53
Z-*2-decene*	6.322	6.29	6.353	1177140	34

*Data for Table S1: Entry **20***

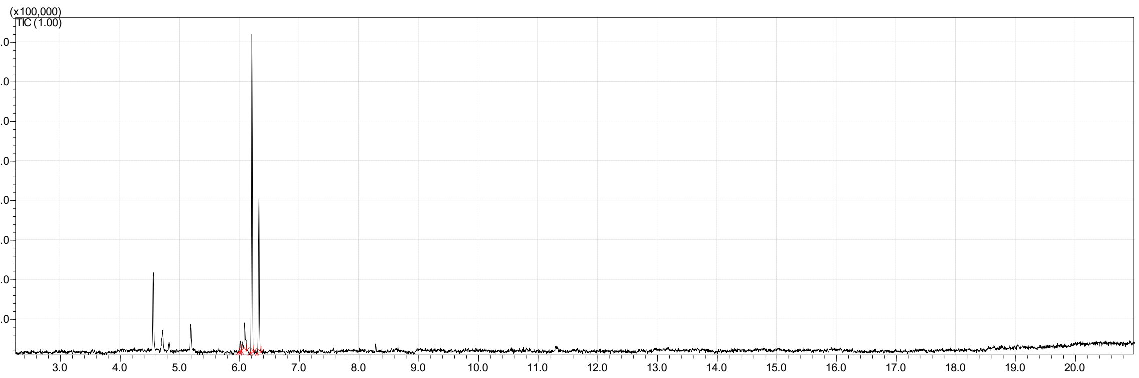

Figure S39. GC-MS Trace of Table S1: Entry 20; 20 mol% Pd(cod)Cl_2/BCF/Et_3Si-H.

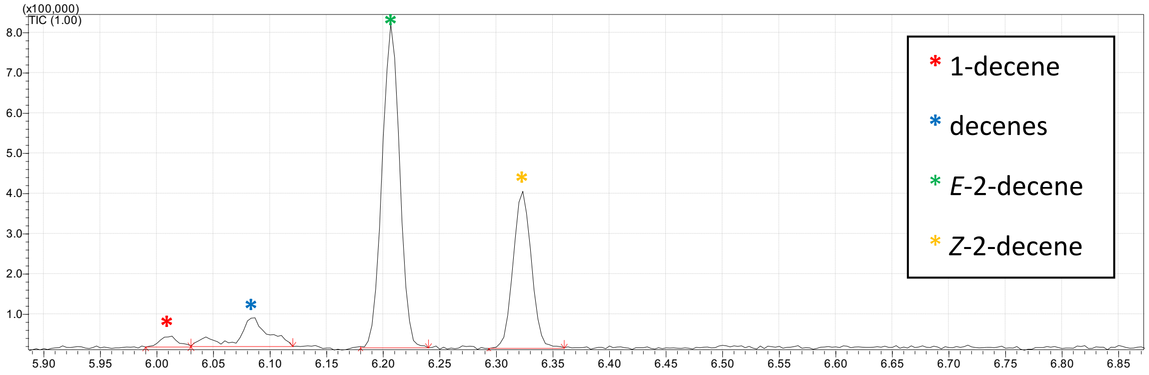

Figure S40. GC-MS Trace of Table S1: Entry 20; 20 mol% Pd(cod)Cl_2/BCF/Et_3Si-H (Expanded View). Table below denoting peak identity, retention times, and areas of the peaks.

Table S21. Relative peak integrations for Table S1: entry 20

Compound	*RT*	*Start Time*	*End Time*	*Area*	*% Area*
1-decene	6.012	5.99	6.03	30045	2
decenes	6.085	6.03	6.12	138347	9
E-*2-decene*	6.207	6.18	6.24	874011	59
Z-*2-decene*	6.323	6.293	6.36	439769	30

*Data for Table S1: Entry **21***

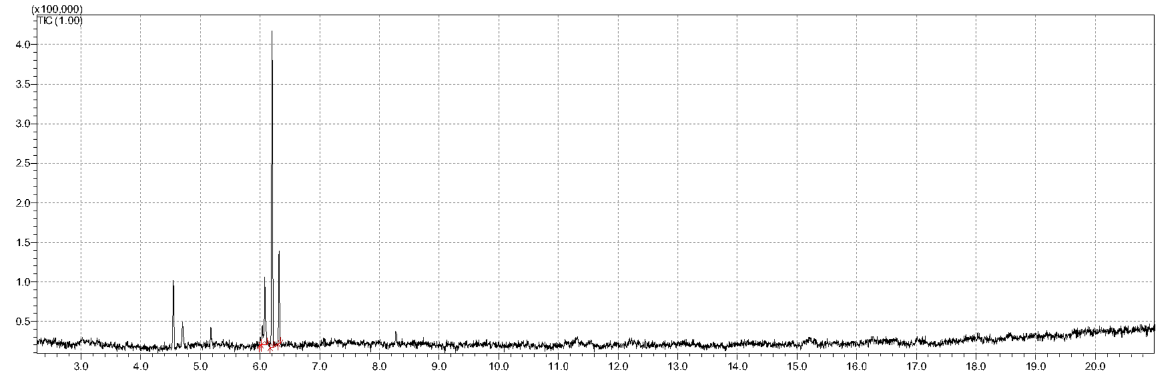

Figure S41. GC-MS Trace of Table S1: Entry 21; 24 hours.

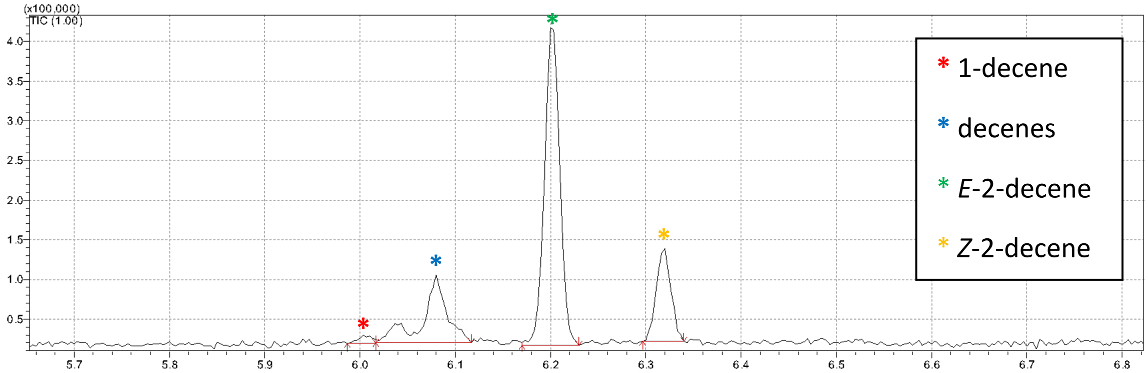

Figure S42. GC-MS Trace of Table S1: Entry 21; 24 hours (Expanded View). Table below denoting peak identity, retention times, and areas of the peaks.

Table S22. Relative peak integrations for Table S1: entry 21

Compound	*RT*	*Start Time*	*End Time*	*Area*	*% Area*
1-decene	6.004	5.987	6.017	10799	1
decenes	6.08	6.017	6.117	150291	20
E-*2-decene*	6.201	6.17	6.23	455875	61
Z-2-decene	6.319	6.297	6.34	126247	17

*Data for Table S1: Entry **22***

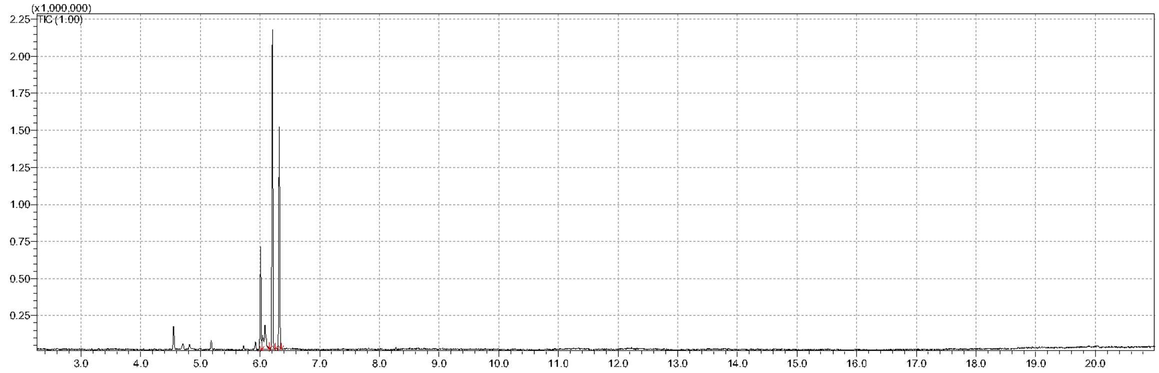

Figure S43. GC-MS Trace of Table S1: Entry 22; 1 hr timepoint; pre-addition of additional 0.25 mmol 1-decene.

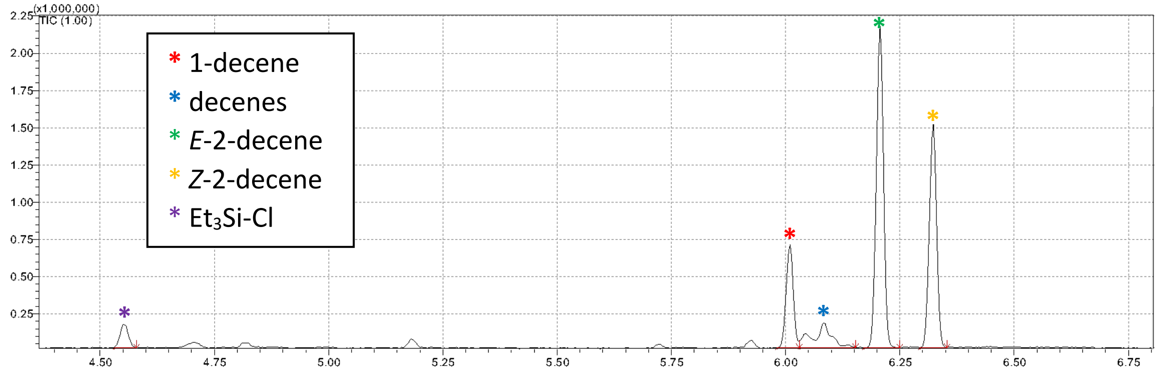

Figure S44. GC-MS Trace of Table S1: Entry 22; 1 hr timepoint; pre-addition of additional 0.25 mmol 1-decene (Expanded View). Table below denoting peak identity, retention times, and areas of the peaks.

Table S23. Relative peak integrations for Table S1: entry 22

Compound	*RT*	*Start Time*	*End Time*	*Area*	*% Area*
Et_3Si-Cl	4.551	4.53	4.58	202049	4
1-decene	6.009	5.98	6.03	767446	14
decenes	6.084	6.03	6.153	447069	8
E-*2-decene*	6.207	6.17	6.25	2376024	44
Z-*2-decene*	6.323	6.293	6.353	1669465	31

At 1 hour, the reaction had gone to 85% completion. Given that $Et_3Si\text{-}Cl$ is a nonreactive side product of the PdNP formation, comparison between the relative areas of the $Et_3Si\text{-}Cl$ and the total area of olefins can give information and the subsequent conversion of more 1-decene to isomerized products. This ratio is equal to $Et_3Si\text{-}Cl_{Area}/Olefins_{Area} = 0.04$.

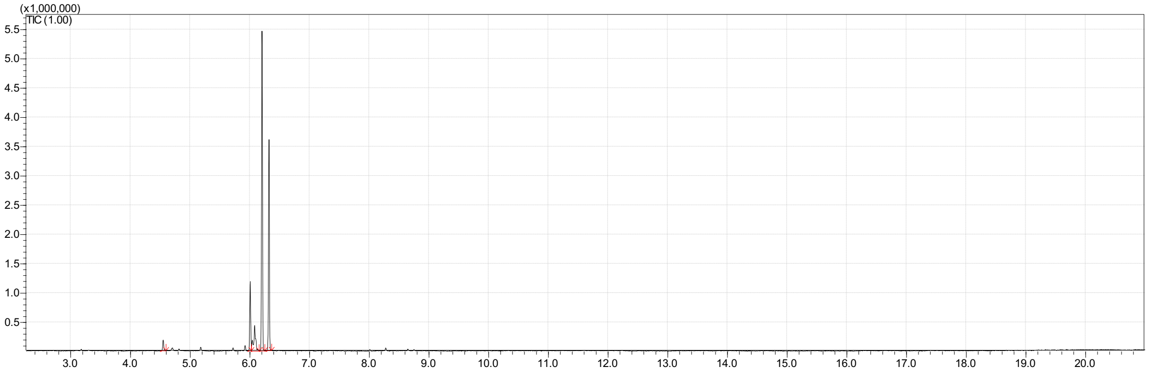

Figure S45. GC-MS Trace of Table S1: Entry 22; 4 hr timepoint; post-addition of additional 0.25 mmol 1-decene.

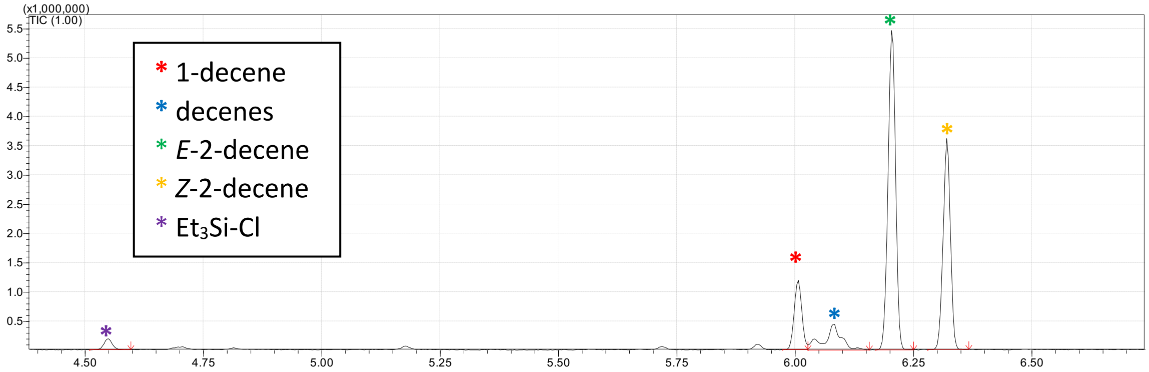

Figure S46. GC-MS Trace of Table S1: Entry 22; 4 hr timepoint; post-addition of additional 0.25 mmol 1-decene (Expanded View). Table below denoting peak identity, retention times, and areas of the peaks.

Table S24. Relative peak integrations for Table S1: entry 22

Compound	*RT*	*Start Time*	*End Time*	*Area*	*% Area*
$Et_3Si\text{-}Cl$	4.549	4.51	4.597	237334	2
1-decene	6.006	5.973	6.027	1346648	11
decenes	6.082	6.027	6.157	1089103	9
E-2-decene	6.204	6.17	6.25	6082958	48
Z-2-decene	6.32	6.28	6.367	3881107	31

At 4 hours, the reaction had gone to 89% completion. Given that $Et_3Si\text{-}Cl$ is a nonreactive side product of the PdNP formation, comparison between the relative areas of the $Et_3Si\text{-}Cl$ and the total area of olefins can give information and the subsequent conversion of more 1-decene to isomerized products. This ratio is equal to $Et_3Si\text{-}Cl_{Area}/Olefins_{Area} = 0.02$. This shows that the catalyst will continue to catalyze isomerization once the reaction has finished.

*Data for Table S1: Entry **23***

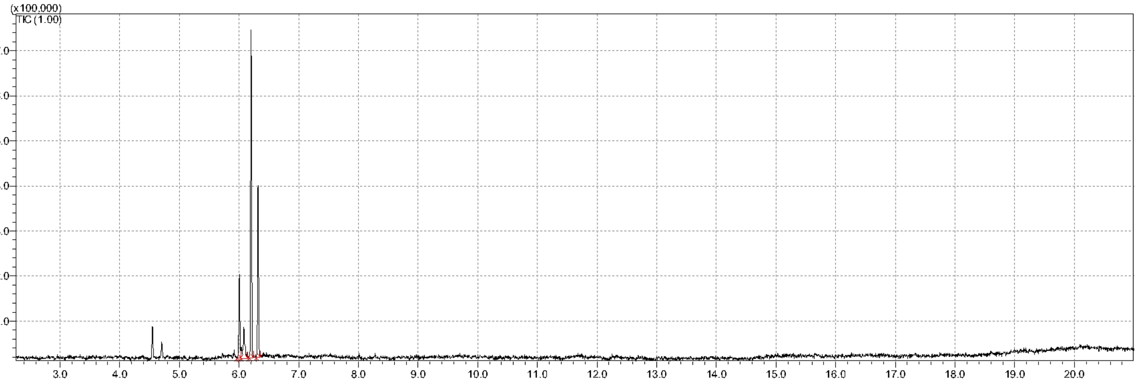

Figure S47. GC-MS Trace of Table S1: Entry 23; 10 mol% $Et_3Si\text{-}H$

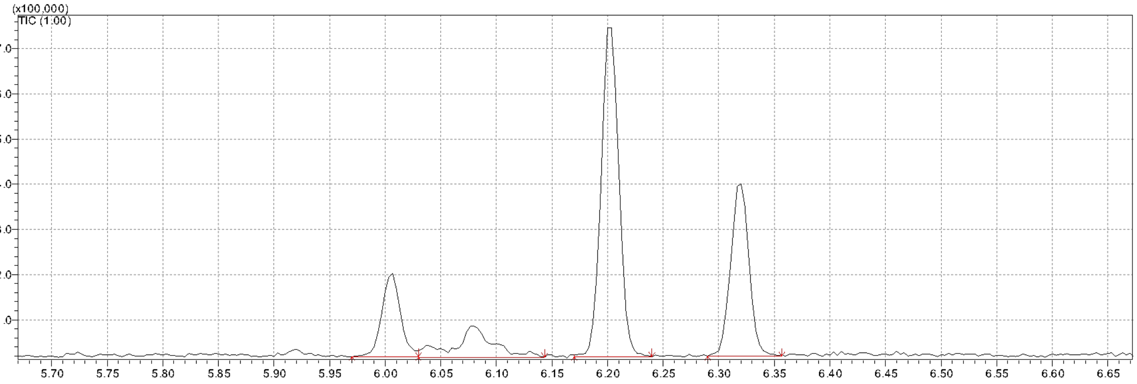

Figure S48. GC-MS Trace of Table S1: Entry 23; 10 mol% $Et_3Si\text{-}H$ (Expanded View). Table below denoting peak identity, retention times, and areas of the peaks.

Table S25. Relative peak integrations for Table S1: entry 23

Compound	*RT*	*Start Time*	*End Time*	*Area*	*% Area*
1-decene	6.005	5.97	6.03	217024	13
decenes	6.078	6.03	6.143	162319	10
E-*2-decene*	6.202	6.17	6.24	816800	50
Z-*2-decene*	6.319	6.29	6.357	440670	27

*Data for Table S1: Entry **24***

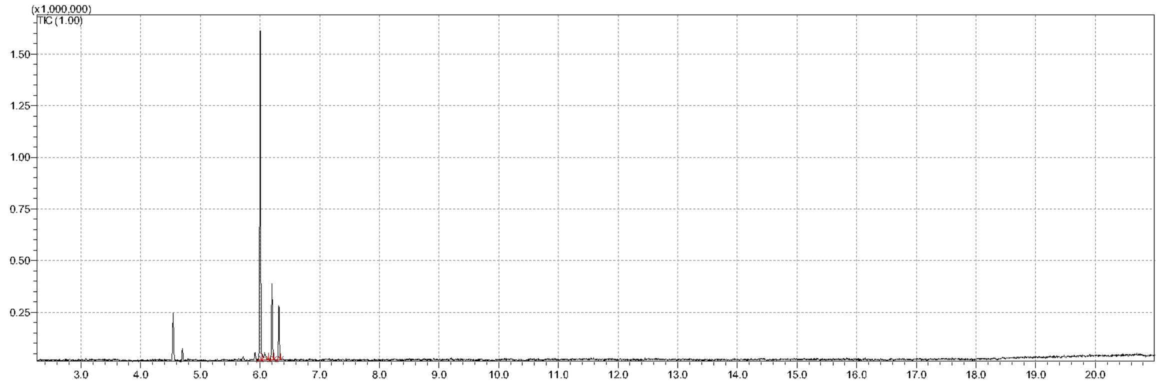

Figure S49. GC-MS Trace of Table S1: Entry 24; 20 mol% Et_3Si-H

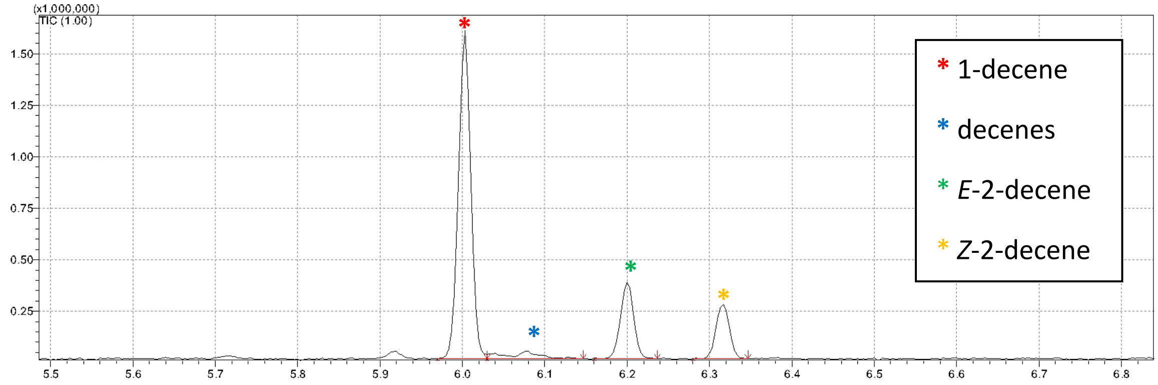

Figure S50. GC-MS Trace of Table S1: Entry 24; 20 mol% Et_3Si-H (Expanded View). Table below denoting peak identity, retention times, and areas of the peaks.

Table S26. Relative peak integrations for entry Table S1: 24

Compound	*RT*	*Start Time*	*End Time*	*Area*	*% Area*
1-decene	6.003	5.97	6.03	1669465	68
decenes	6.078	6.03	6.147	88780	4
E-*2-decene*	6.2	6.16	6.237	410548	17
Z-*2-decene*	6.316	6.28	6.347	303202	12

GC-MS Traces of Substrate Standards

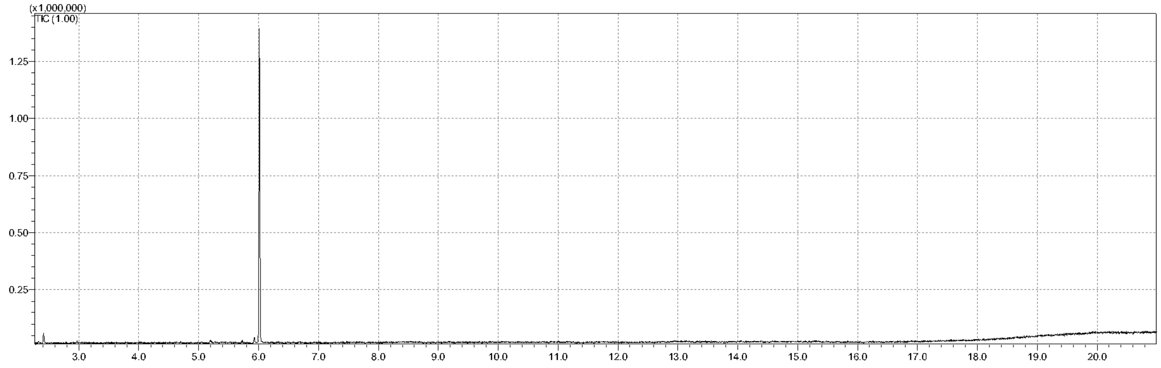

Figure S51. 1-decene GC-MS standard. Retention time of 6.056 min.

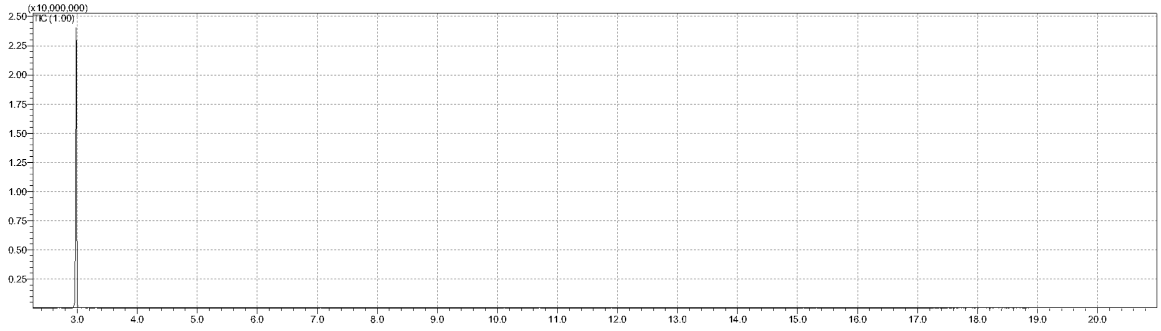

Figure S52. 1-octene GC-MS standard. Retention time of 2.981 min.

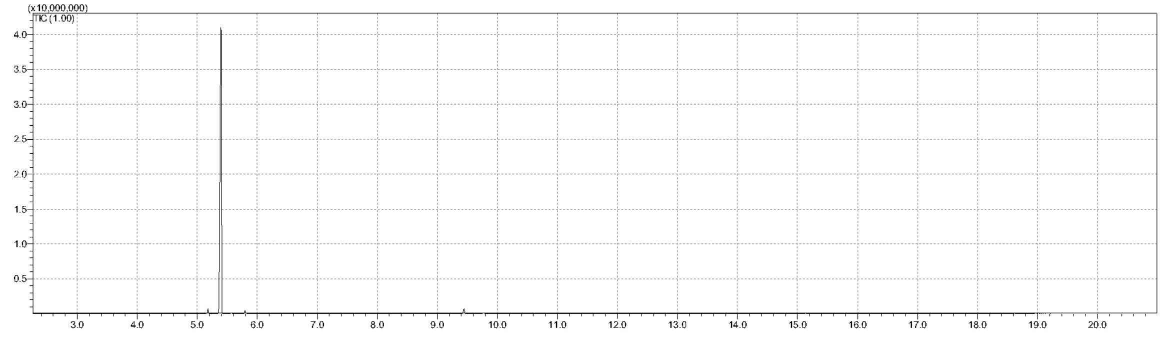

Figure S53. Allylbenzene GCMS standard. Retention time of 5.394 min.

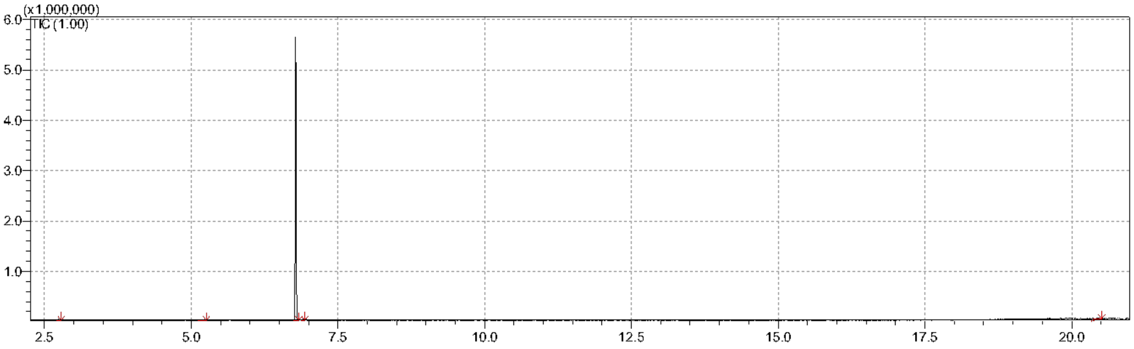

Figure S54. But-3-en-1-ylbenzene GCMS standard. Retention time of 6.779 min.

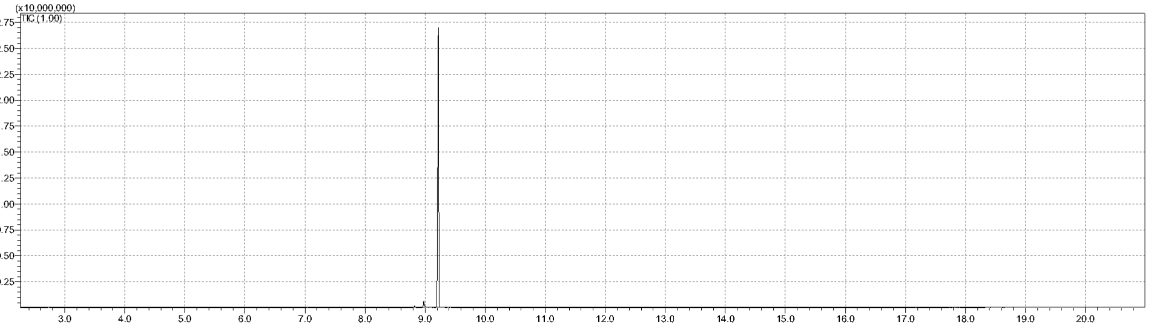

Figure S55. Hex-5-en-1-ylbenzeneGCMS standard. Retention time of 9.219

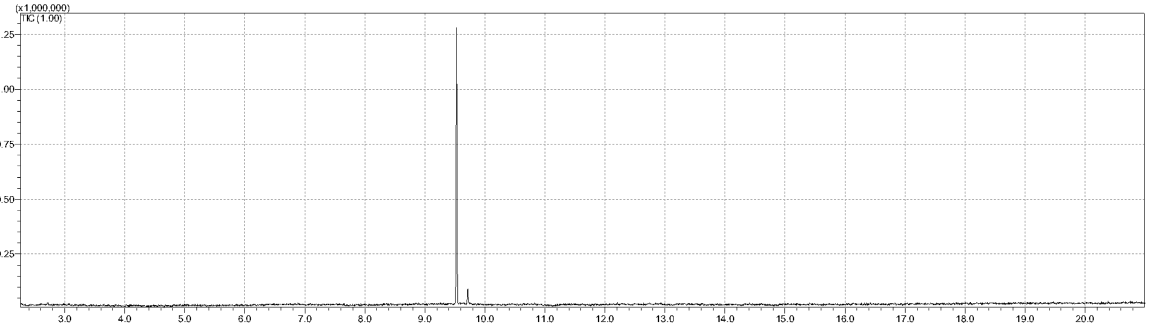

Figure S56. Triethyl(hex-5-en-1-yloxy)silane GCMS standard. Retention time of 9.527 min.

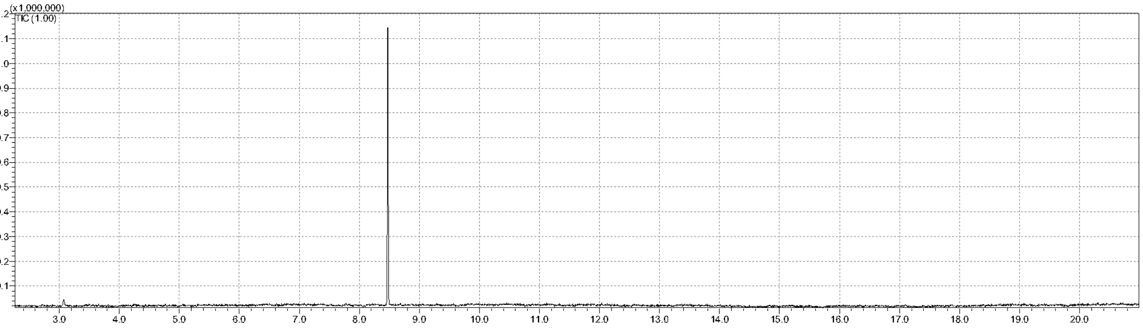

Figure S57. Tert-butyl(hex-5-en-1-yloxy)dimethylsilane GCMS standard. Retention time of 8.474 min.

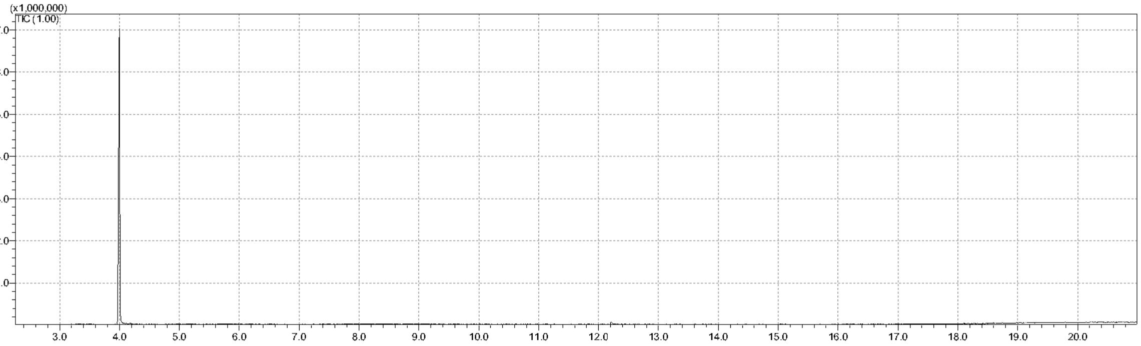

Figure S58. Hex-5-en-1-ol GCMS standard. Retention time of 4.035 min.

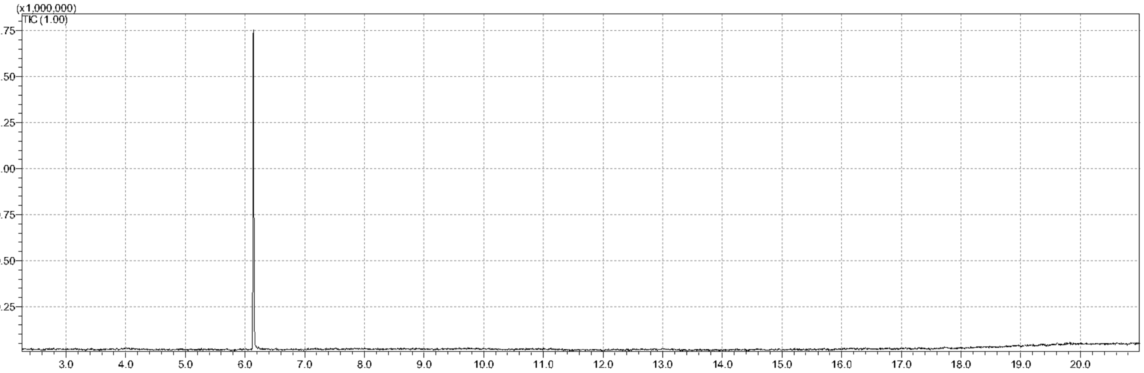

Figure S59. Hex-5-en-1-yl acetate GCMS standard. Retention time of 6.139 min.

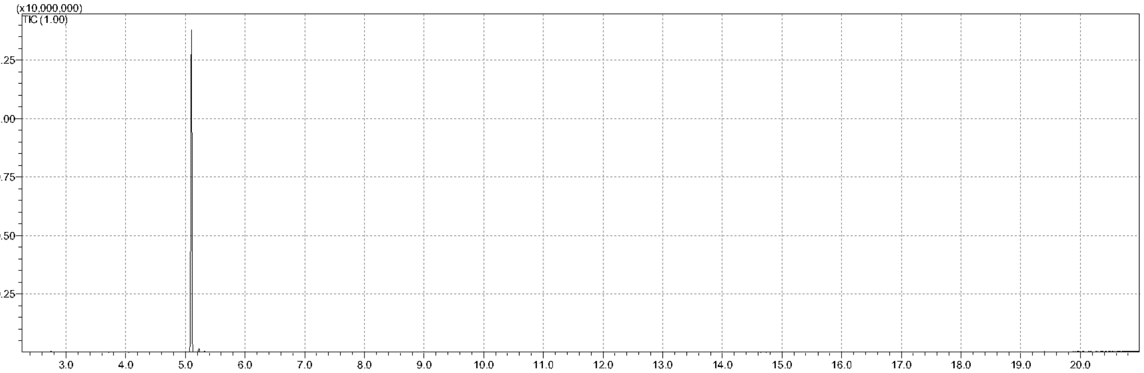

Figure S60. 6-bromohex-1-ene acetate GCMS standard. Retention time of 5.101 min.

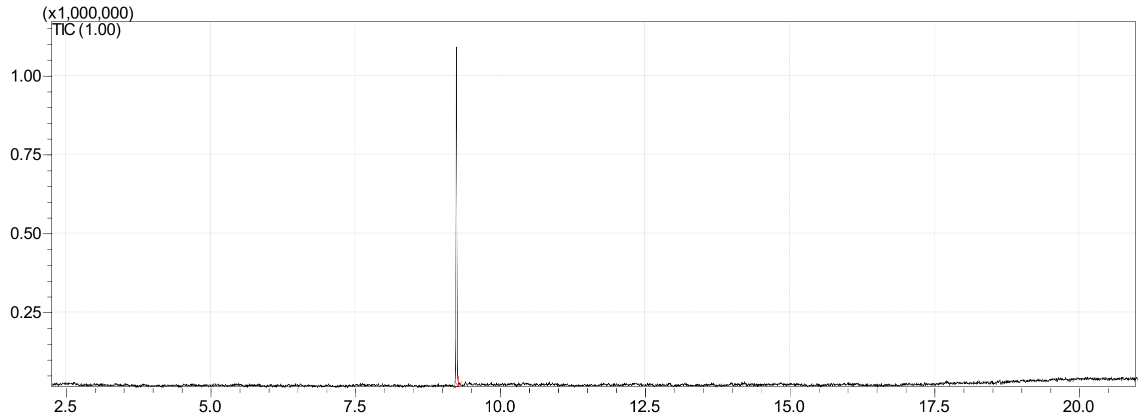

Figure S61. 2-(hex-5-en-1-yl)thiophene GCMS standard. Retention time of 3.319 min.

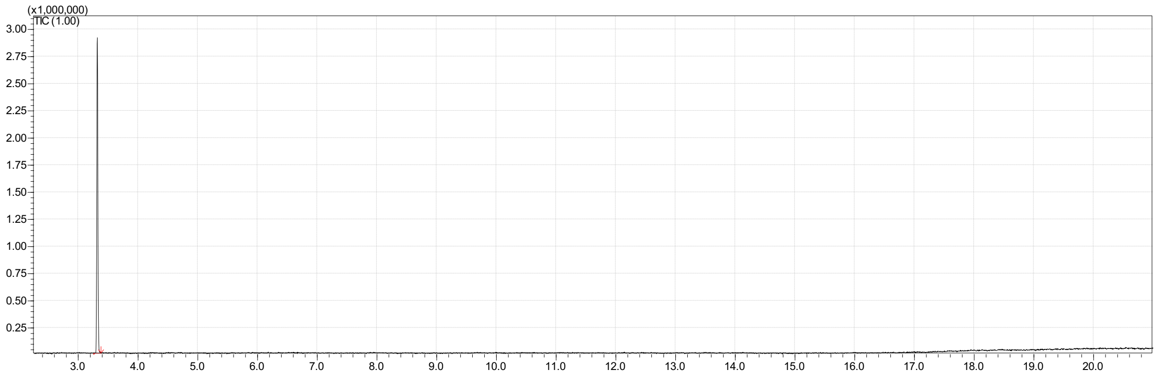

Figure S62. 6-methoxyhex-1-ene GCMS standard. Retention time of 3.319 min.

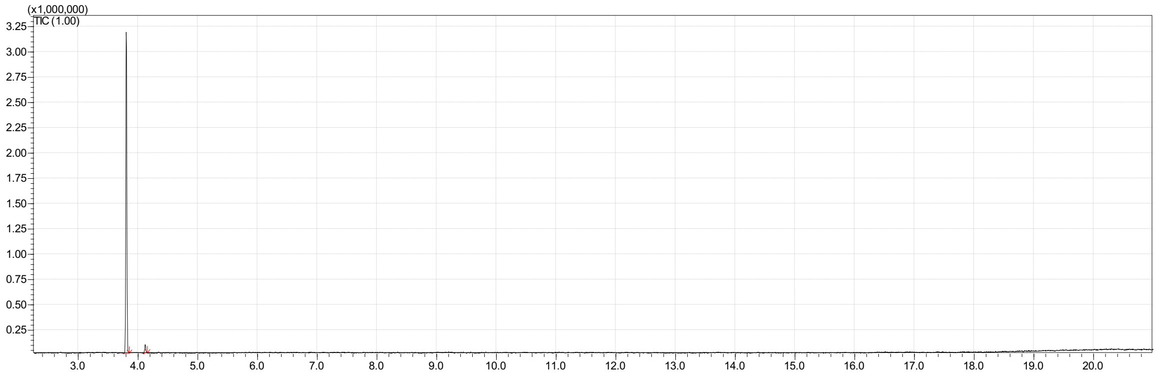

Figure S63. 6-chlorohex-1-ene GCMS standard. Retention time of 3.804 min.

GC-MS Traces of Substrate Screening

Table S27: Substrate Scope

Entry	*Substrate*	*% Conversion*	*% m.i.s.*	*E/Z Ratio*	*Time [hr]*
1	1-decene	96	90	1.9	2
2	hex-5-en-1-yl acetate	87	100	1.7	2
3	but-3-en-1-ylbenzene	94	96	2.0	2
4	hex-5-en-1-ylbenzene	93	92	1.7	2
5	6-bromohex-1-ene	86	98	1.6	2
6	allylbenzene	69	---	0.2	3
7	1-octene	96	89	2.0	2
8	hex-5-en-1-ol	89	100	2.2	2
9	2-(hex-5-en-1-yl)thiophene	86	96	3.1	16
10	triethyl(undec-10-en-1-yloxy)silane	58	98	1.3	7
11	tert-butyldimethyl(undec-10-en-1-yloxy)silane	90	98	1.8	2
12	6-methoxyhex-1-ene	97	100	1.9	7
13	6-chlorohex-1-ene	96	96	1.9	2
14[a]	hex-5-en-1-yl 2-(4-isobutylphenyl)propanoate	76	98	2.3	4

All substrates were screened using standard conditions (Table S1; Entry 1) on a 0.25 mmol scale. % Conversion was determined with GC-MS by seeing how much 1-decene was converted to internal olefins. % Mono-isomerization selectivity (m.i.s.) was determined with GC-MS by seeing what percentage of the isomerized product was mono-isomerized. [a]%Conversion, %m.i.s., and E/Z ratio determined by NMR analysis.

*Data for Table S27: Entry **1***

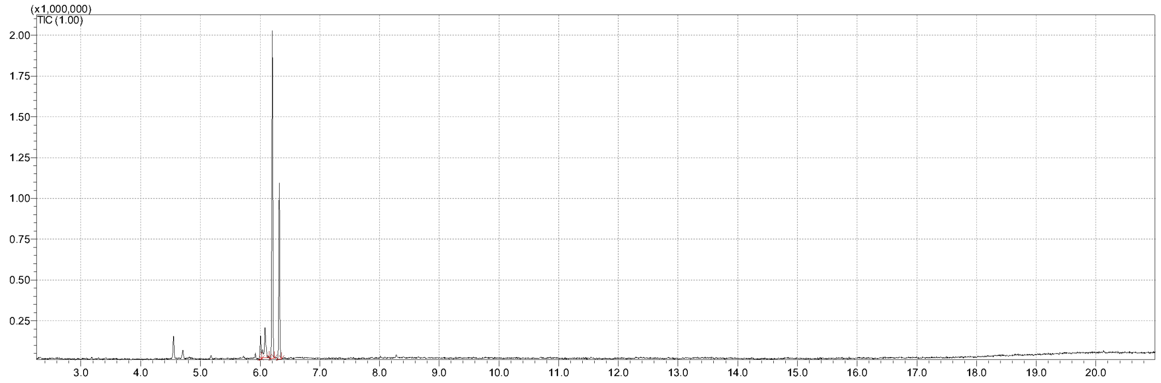

Figure S64. GC-MS Trace of Table S27: Entry 1; 1-decene.

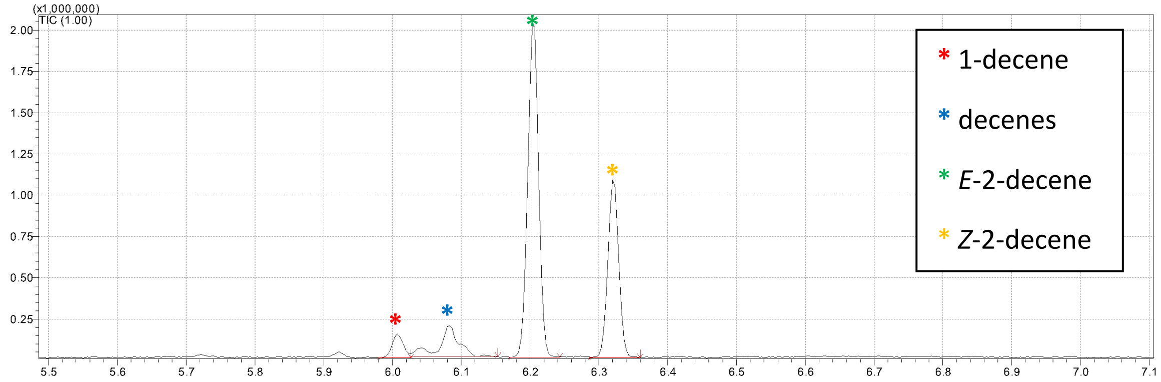

Figure S65. GC-MS Trace of Table S27: Entry 1; 1-decene (Expanded View). Table below denoting peak identity, retention times, and areas of the peaks.

Table S28. Relative peak integrations for Table S27: entry 1

Compound	*RT*	*Start Time*	*End Time*	*Area*	*% Area*
1-decene	6.007	5.98	6.027	168951	4
decenes	6.082	6.027	6.153	363107	9
E-*2-decene*	6.205	6.17	6.243	2232179	56
Z-2-decene	6.321	6.287	6.36	1193802	31

*Data for Table S27: Entry **2***

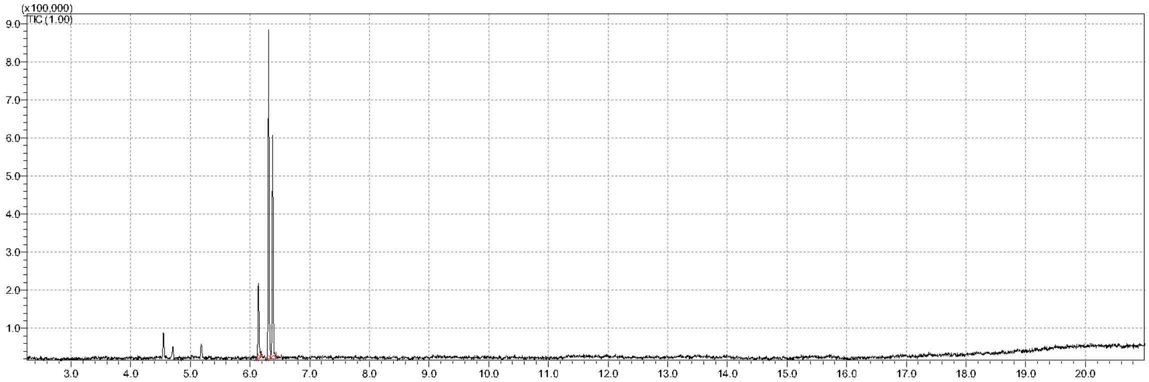

Figure S66. GC-MS Trace of Table S27: Entry 2; hex-5-en-1-yl acetate.

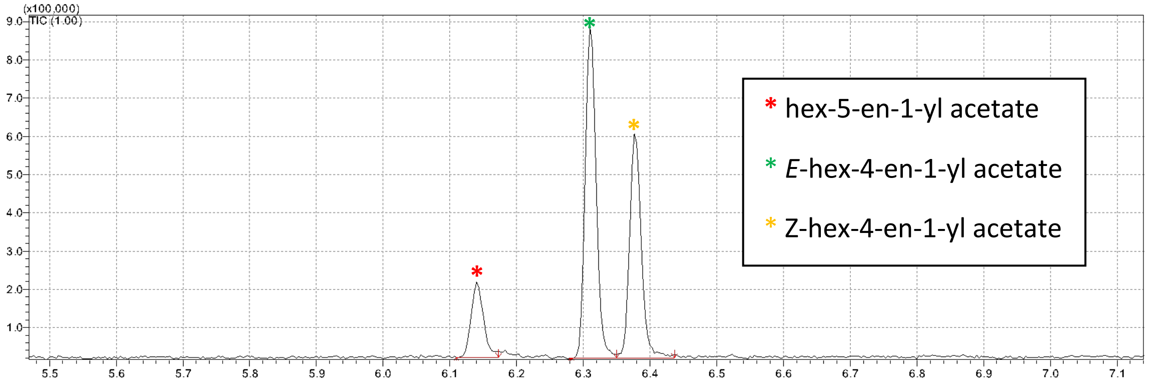

Figure S67. GC-MS Trace of Table S27: Entry 2; hex-5-en-1-yl acetate (Expanded View). Table below denoting peak identity, retention times, and areas of the peaks.

Table S29. Relative peak integrations for Table S27: entry 2

Compound	*RT*	*Start Time*	*End Time*	*Area*	*% Area*
hex-5-en-1-yl acetate	6.141	6.11	6.173	253448	9
E-hex-4-en-1-yl acetate	6.311	6.283	6.437	1703363	58
Z-hex-4-en-1-yl acetate	6.311	6.28	6.35	986604	34

*Data for Table S27: Entry **3***

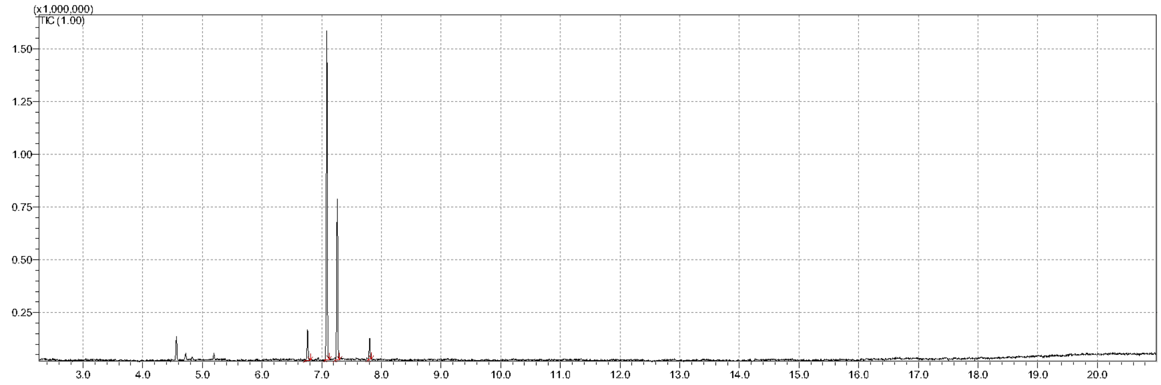

Figure S68. GC-MS Trace of Table S27: Entry 3; but-3-en-1-ylbenzene.

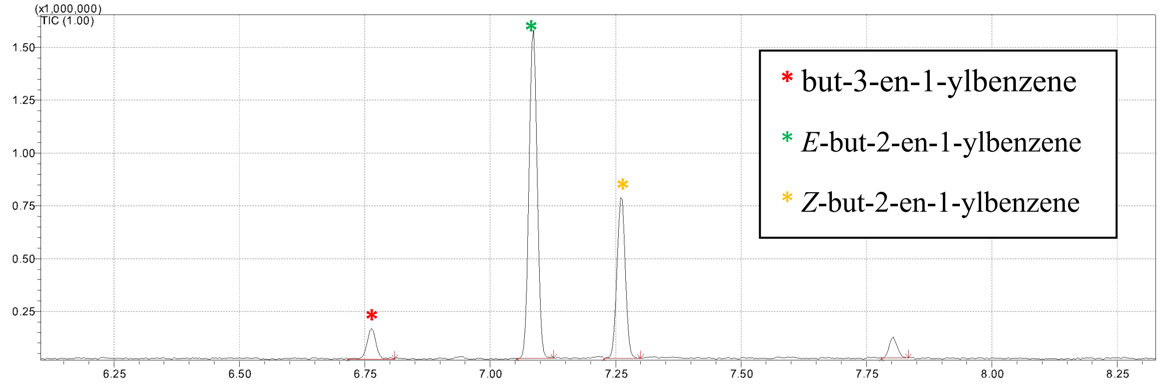

Figure S69. GC-MS Trace of Table S27: Entry 3; but-3-en-1-ylbenzene (Expanded View). Table below denoting peak identity, retention times, and areas of the peaks.

Table S30. Relative peak integrations for Table S27: entry 3

Compound	*RT*	*Start Time*	*End Time*	*Area*	*% Area*
but-3-en-1-ylbenzene	6.763	6.717	6.81	183541	6
E-*but-2-en-1-ylbenzene*	7.086	7.053	7.127	1761319	62
Z-*but-2-en-1-ylbenzene*	7.261	7.227	7.3	889355	31

*Table S27: Entry **4***

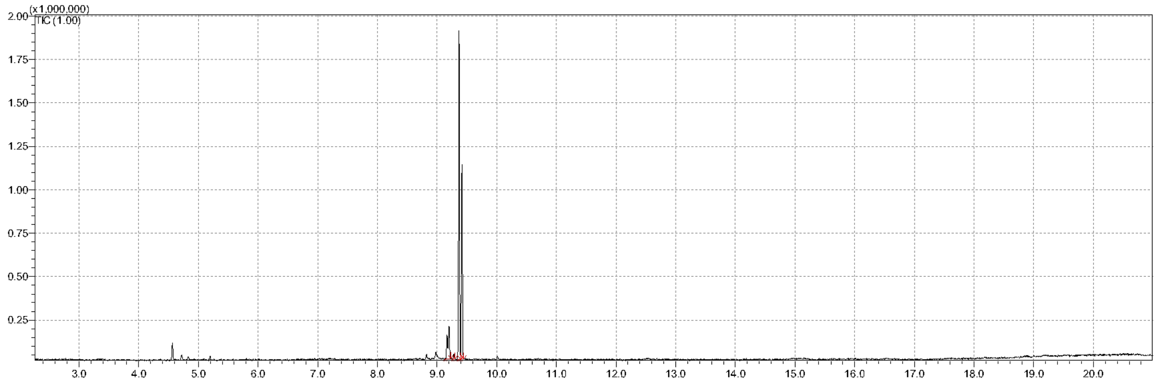

Figure S70. GC-MS Trace of Table S27: Entry 4; hex-5-en-1-ylbenzene.

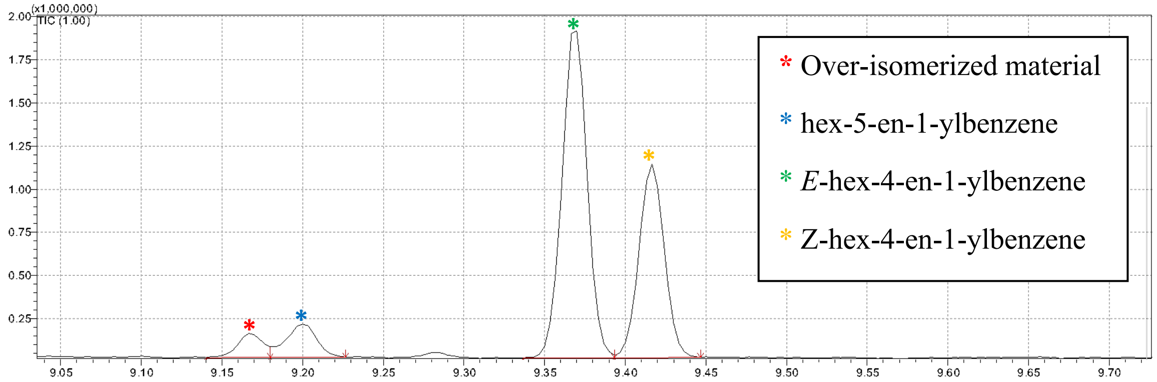

Figure S71. GC-MS Trace of Table S27: Entry 4; hex-5-en-1-ylbenzene (Expanded View). Table below denoting peak identity, retention times, and areas of the peaks.

Table S31. Relative peak integrations for Table S27: entry 4

Compound	*RT*	*Start Time*	*End Time*	*Area*	*% Area*
Over-isomerization	9.167	9.14	9.18	160753	4
hex-5-en-1-ylbenzene	9.2	9.18	9.227	253059	7
E-*hex-4-en-1-ylbenzene*	9.368	9.337	9.393	2075629	54
Z-hex-4-en-1-ylbenzene	9.416	9.393	9.447	1227262	32

Data for Table S27: Entry ***5***

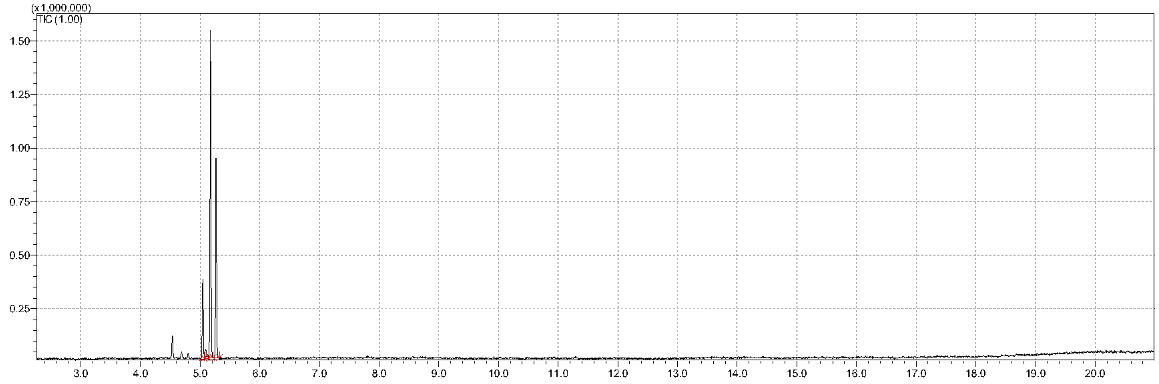

Figure S72. GC-MS Trace of Table S27: Entry 5; 6-bromohex-1-ene.

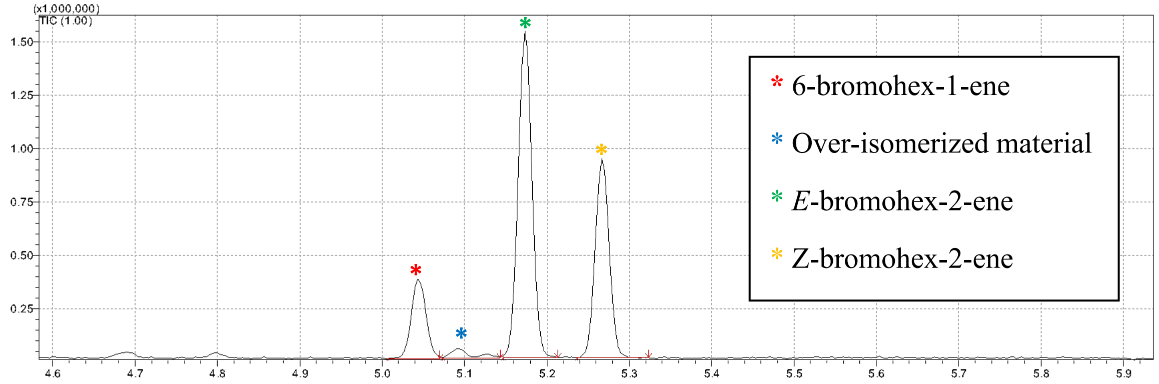

Figure S73. GC-MS Trace of Table S27: Entry 5; 6-bromohex-1-ene (Expanded View). Table below denoting peak identity, retention times, and areas of the peaks.

Table S32. Relative peak integrations for Table S27: entry 5

Compound	*RT*	*Start Time*	*End Time*	*Area*	*% Area*
6-bromohex-1-ene	5.044	5.007	5.07	468682	14
Over-isomerization	5.092	5.073	5.143	72756	2
E-*6-bromohex-2-ene*	5.174	5.147	5.213	1804138	52
Z-*6-bromohex-2-ene*	5.267	5.237	5.323	1110968	32

Data for Table S27: Entry ***6***

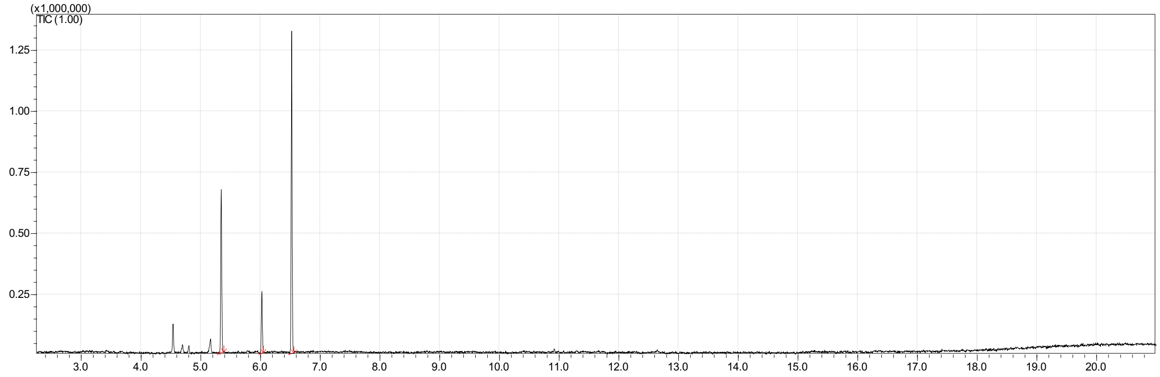

Figure S74. GC-MS Trace of Table S27: Entry 6; allylbenzene.

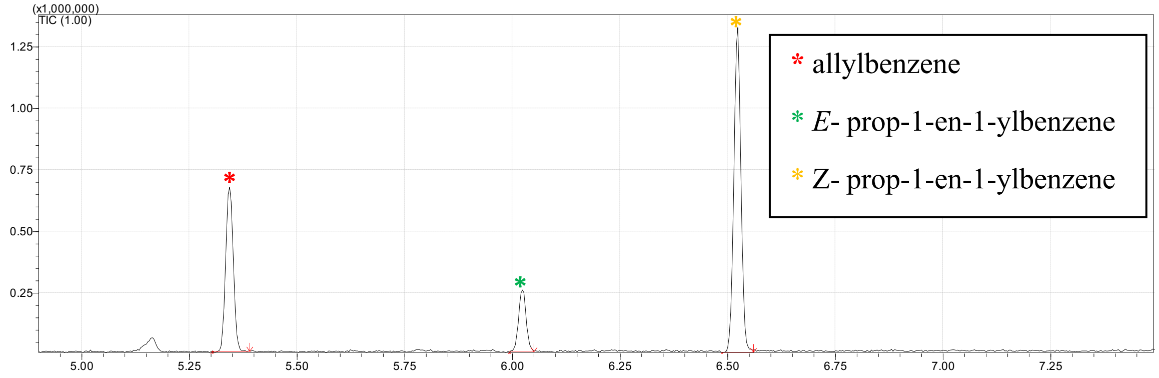

Figure S75. GC-MS Trace of Table S27: Entry 6; allylbenzene (Expanded View). Table below denoting peak identity, retention times, and areas of the peaks.

Table S33. Relative peak integrations for Table S27: entry 6

Compound	*RT*	*Start Time*	*End Time*	*Area*	*% Area*
allylbenzene	5.343	5.303	5.39	814391	31
E- *prop-1-en-1-ylbenzene*	6.023	5.993	6.05	312934	12
Z- *prop-1-en-1-ylbenzene*	6.522	6.487	6.56	1521996	57

Data for Table S27: Entry 7

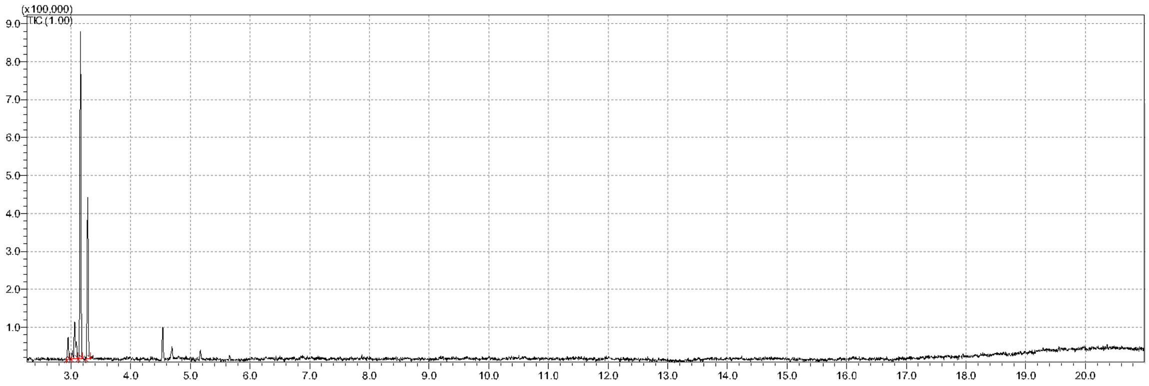

Figure S76. GC-MS Trace of Table S27: Entry 7; 1-octene.

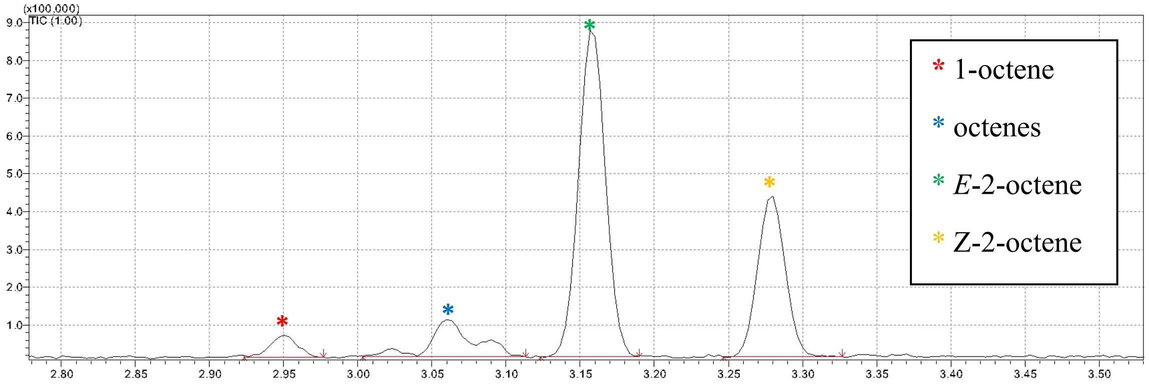

Figure S77. GC-MS Trace of Table S27: Entry 7; 1-octene (Expanded View). Table below denoting peak identity, retention times, and areas of the peaks.

Table S34. Relative peak integrations for Table S27: entry 7

Compound	*RT*	*Start Time*	*End Time*	*Area*	*% Area*
1-octene	2.95	2.923	2.977	74770	4
octenes	3.06	3.003	3.113	202157	11
E-2-octene	3.158	3.123	3.19	1095561	57
Z-2-octene	3.279	3.247	3.327	552022	29

*Data for Table S27: Entry **8***

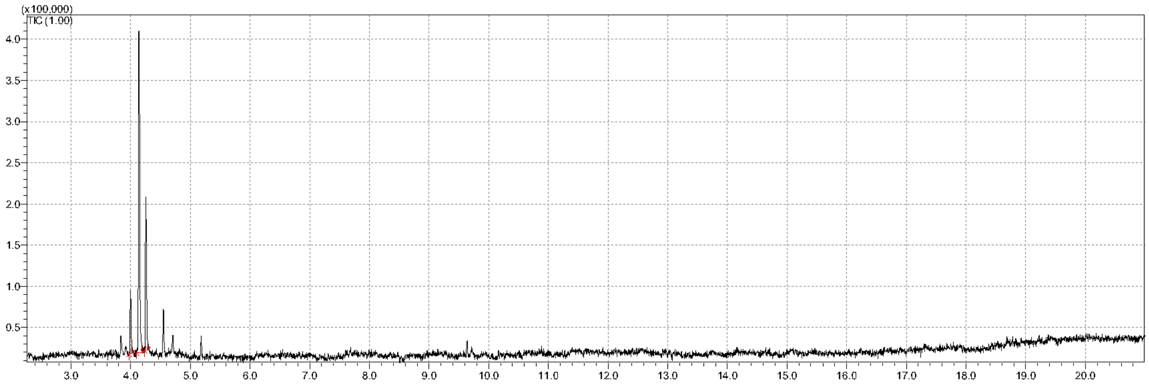

Figure S78. GC-MS Trace of Table S27: Entry 8; hex-5-en-1-ol.

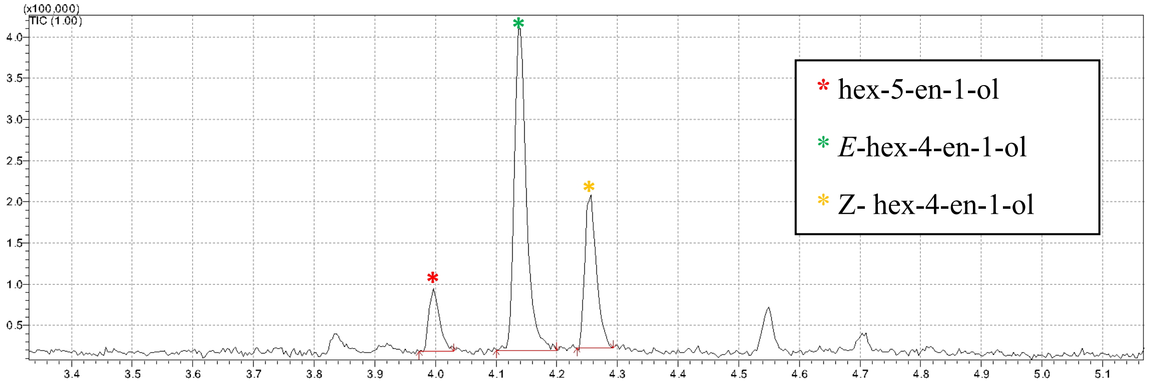

Figure S79. GC-MS Trace of Table S27: Entry 8; hex-5-en-1-ol (Expanded View). Table below denoting peak identity, retention times, and areas of the peaks.

Table S35. Relative peak integrations for Table S27: entry 8

Compound	*RT*	*Start Time*	*End Time*	*Area*	*% Area*
hex-5-en-1-ol	3.997	3.973	4.03	94828	11
E-hex-4-en-1-ol	4.138	4.1	4.2	546533	62
Z- hex-4-en-1-ol	4.256	4.233	4.293	247114	28

*Data for Table S27: Entry **9***

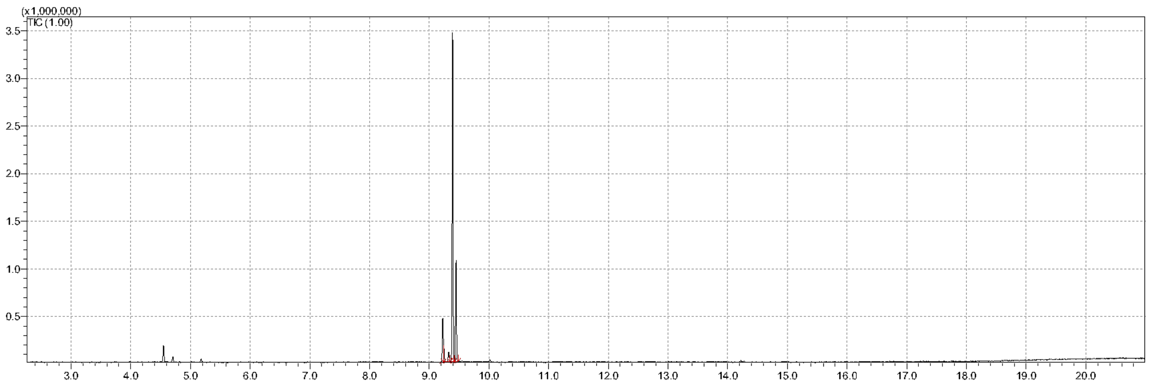

Figure S80. GC-MS Trace of Table S27: Entry 9; 2-(hex-5-en-1-yl)thiophene.

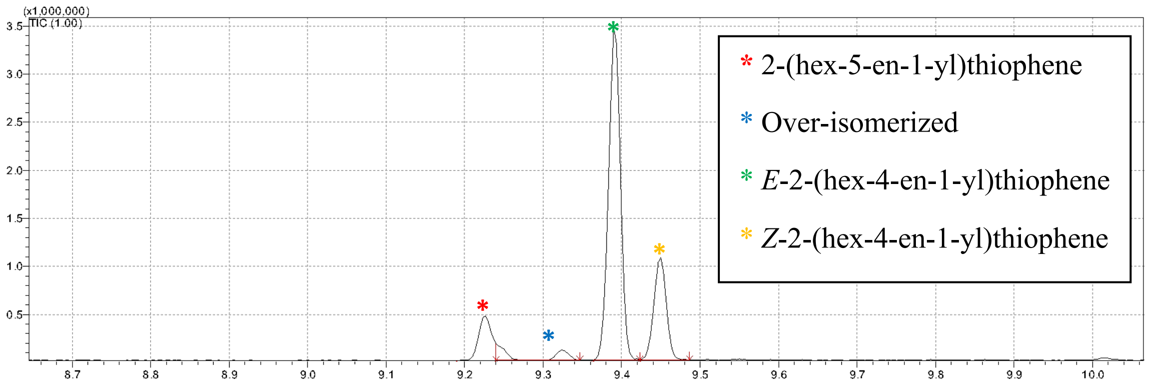

Figure S81. GC-MS Trace of Table S27: Entry 9; 2-(hex-5-en-1-yl)thiophene (Expanded View). Table below denoting peak identity, retention times, and areas of the peaks.

Table S36. Relative peak integrations for Table S27: entry 9

Compound	*RT*	*Start Time*	*End Time*	*Area*	*% Area*
2-(hex-5-en-1-yl)thiophene	9.226	9.19	9.24	551793	10
Over-isomerized	9.243	9.24	9.347	223996	4
E-*2-(hex-4-en-1-yl)thiophene*	9.391	9.363	9.423	3660239	65
Z-2-(hex-4-en-1-yl)thiophene	9.449	9.423	9.487	1168386	21

*Data for Table S27: Entry **10***

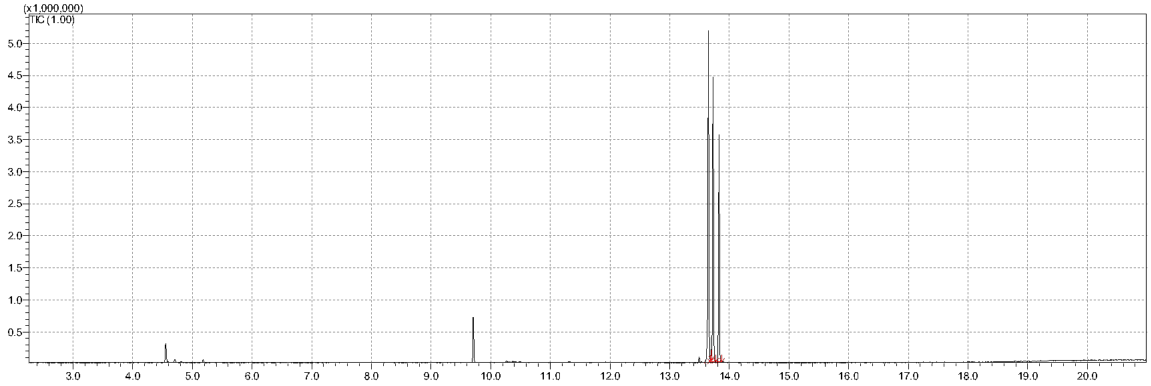

Figure S82. GC-MS Trace of Table S27: Entry 10; triethyl(undec-10-en-1-yloxy)silane.

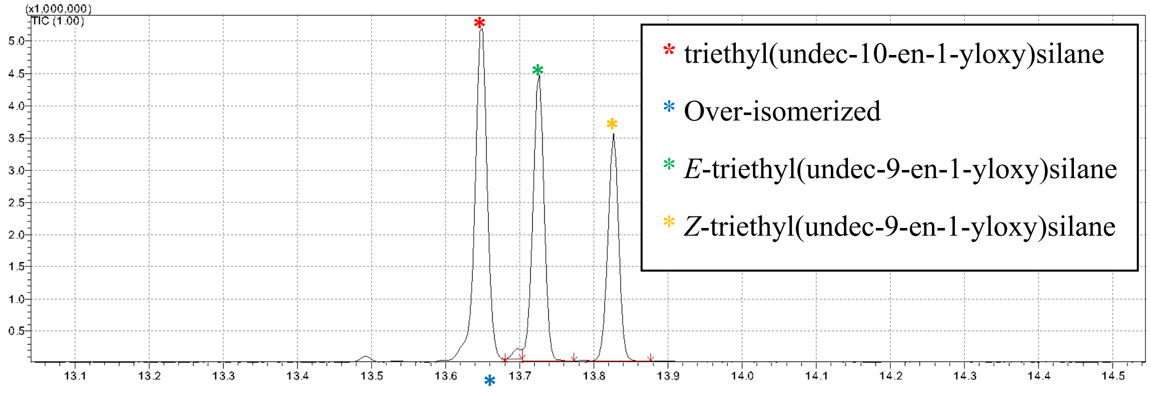

Figure S83. GC-MS Trace of Table S27: Entry 10; triethyl(undec-10-en-1-yloxy)silane (Expanded View). Table below denoting peak identity, retention times, and areas of the peaks.

Table S37. Relative peak integrations for Table S27: entry 10

Compound	*RT*	*Start Time*	*End Time*	*Area*	*% Area*
triethyl(undec-10-en-1-yloxy)silane	13.648	13.587	13.68	6073687	42
Over-isomerized	13.697	13.68	13.703	147966	1
E-*triethyl(undec-9-en-1-yloxy)silane*	13.726	13.703	13.773	4673430	32
Z-*triethyl(undec-9-en-1-yloxy)silane*	13.827	13.79	13.877	3590271	25

*Table S27: Entry **11***

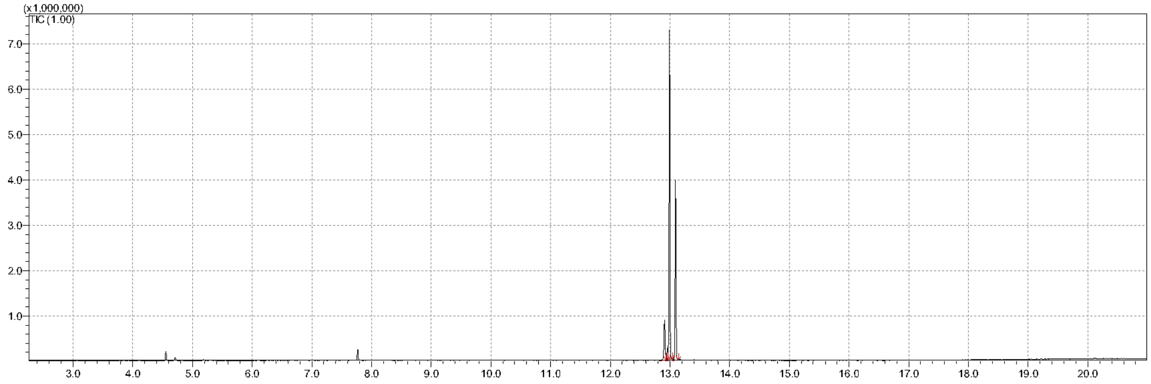

Figure S84. GC-MS Trace of Table S27: Entry 11; tert-butyldimethyl(undec-10-en-1-yloxy)silane.

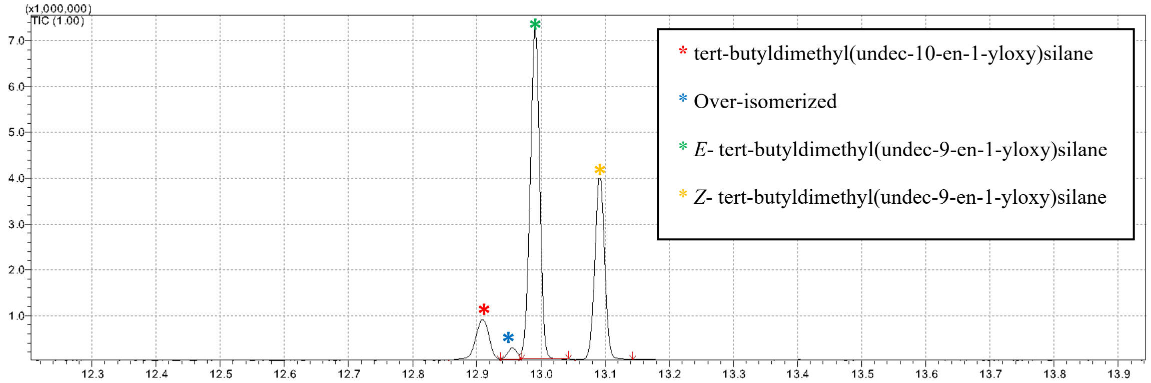

Figure S85. GC-MS Trace of Table S27: Entry 10; tert-butyldimethyl(undec-10-en-1-yloxy)silane (Expanded View). Table below denoting peak identity, retention times, and areas of the peaks.

Table S38. Relative peak integrations for Table S27: entry 10

Compound	*RT*	*Start Time*	*End Time*	*Area*	*% Area*
tert-butyldimethyl(undec-10-en-1-yloxy)silane	12.909	12.857	12.937	1302681	10
Over-isomerized	12.956	12.937	12.97	285349	2
E-*tert-butyldimethyl(undec-9-en-1-yloxy)silane*	12.991	12.967	13.043	7445912	56
Z-*tert-butyldimethyl(undec-9-en-1-yloxy)silane*	13.092	13.053	13.143	4264092	32

Table S27: Entry ***12***

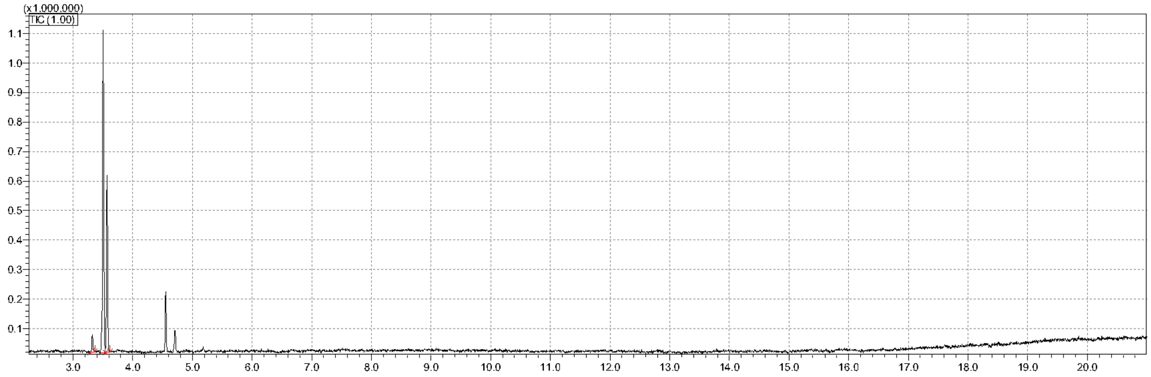

Figure S86. GC-MS Trace of Table S27: Entry 12; 6-methoxyhex-1-ene.

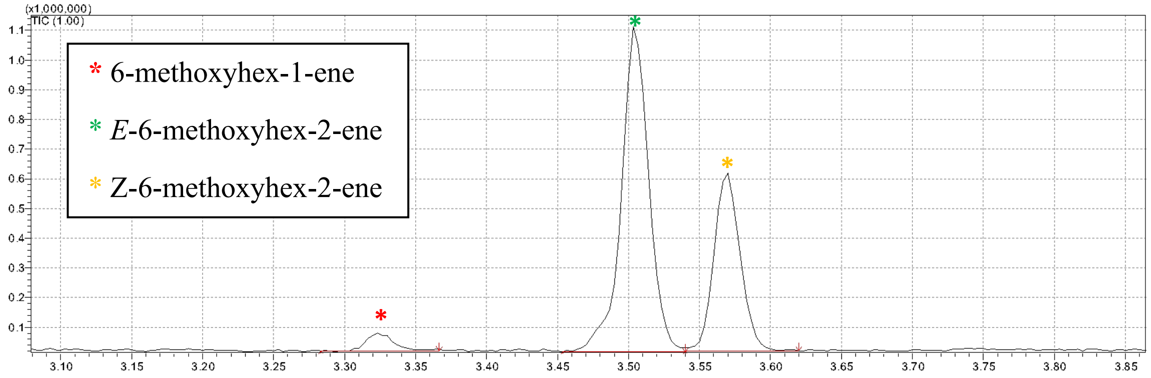

Figure S87. GC-MS Trace of Table S27: Entry 12; 6-methoxyhex-1-ene (Expanded View). Table below denoting peak identity, retention times, and areas of the peaks.

Table S39. Relative peak integrations for Table S27: entry 12

Compound	*RT*	*Start Time*	*End Time*	*Area*	*% Area*
6-methoxyhex-1-ene	3.323	3.283	3.367	79730	3
E-*6-methoxyhex-2-ene*	3.504	3.453	3.54	1479319	63
Z-6-methoxyhex-2-ene	3.569	3.54	3.62	771037	33

*Table S27: Entry **13***

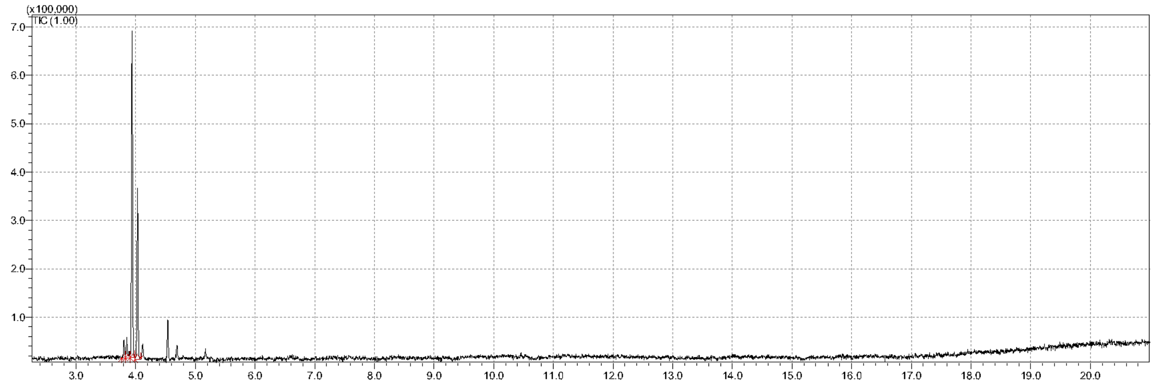

Figure S88. GC-MS Trace of Table S27: Entry 13; 6-chlorohex-1-ene.

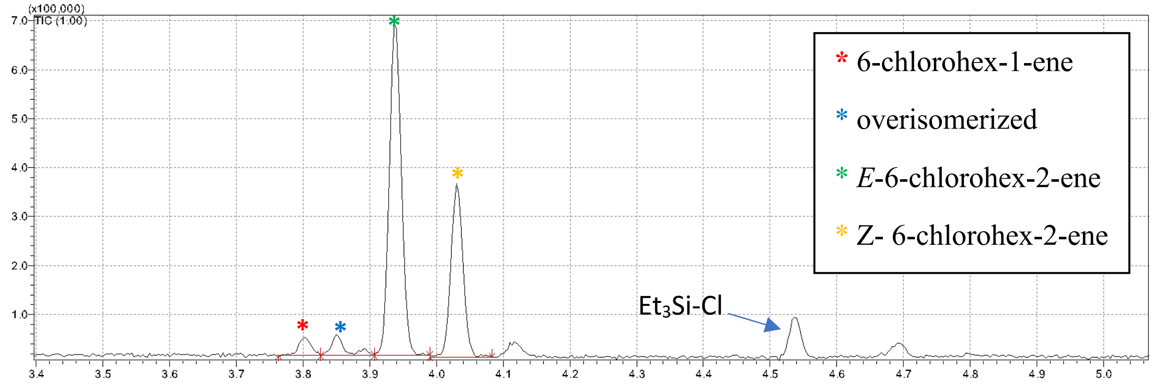

Figure S89. GC-MS Trace of Table S27: Entry 13; 6-chlorohex-1-ene (Expanded View). Table below denoting peak identity, retention times, and areas of the peaks.

Table S40. Relative peak integrations for Table S27: entry 13

Compound	*RT*	*Start Time*	*End Time*	*Area*	*% Area*
6-chlorohex-1-ene	3.803	3.763	3.827	50135	4
Over-isomerized	3.85	3.827	3.907	63274	4
E-*6-chlorohex-2-ene*	3.938	3.907	3.99	852535	60
Z-*6-chlorohex-2-ene*	4.031	3.99	4.083	446065	32

Robustness Screening Standard GC-MS Traces

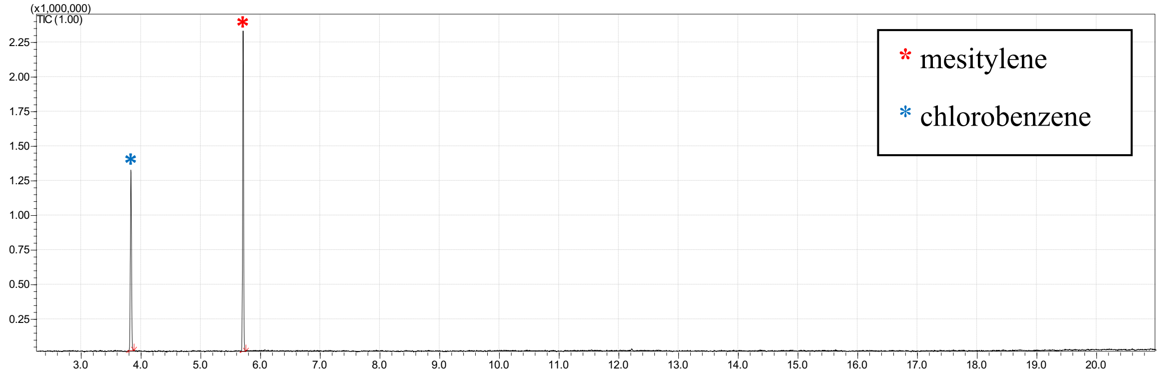

Figure S90. GC-MS trace of an equimolar solution of chlorobenzene and mesitylene. Table below denoting peak identity, retention times, and areas of the peaks.

Table S41. Relative peak integration from Figure S90.

Compound	*RT*	*Start Time*	*End Time*	*Area*	*% Area*
mesitylene	3.831	3.783	3.883	1803877	40
chlorobenzene	5.71	5.667	5.757	2667035	60

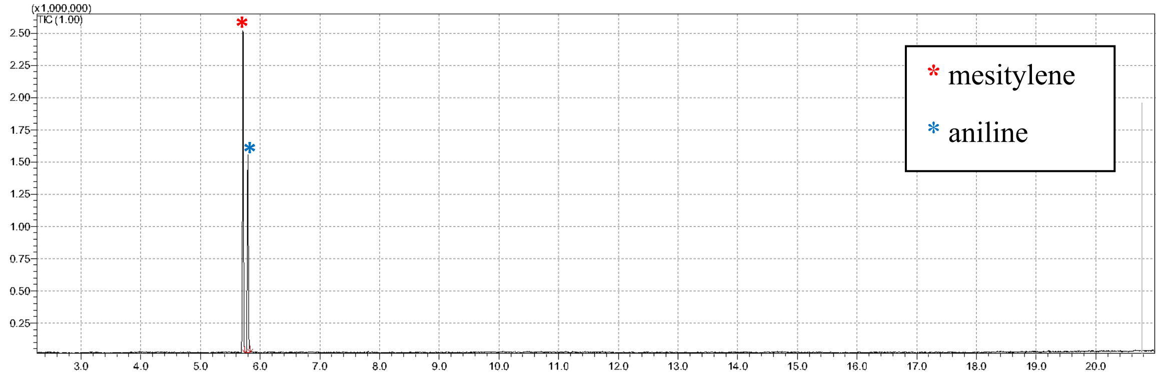

Figure S91. GC-MS trace of an equimolar solution of aniline and mesitylene. Table below denoting peak identity, retention times, and areas of the peaks.

Table S42. Relative peak integration from Figure S91.

Compound	*RT*	*Start Time*	*End Time*	*Area*	*% Area*
mesitylene	5.712	5.677	5.743	2898408	61
aniline	5.793	5.76	5.833	1888563	39

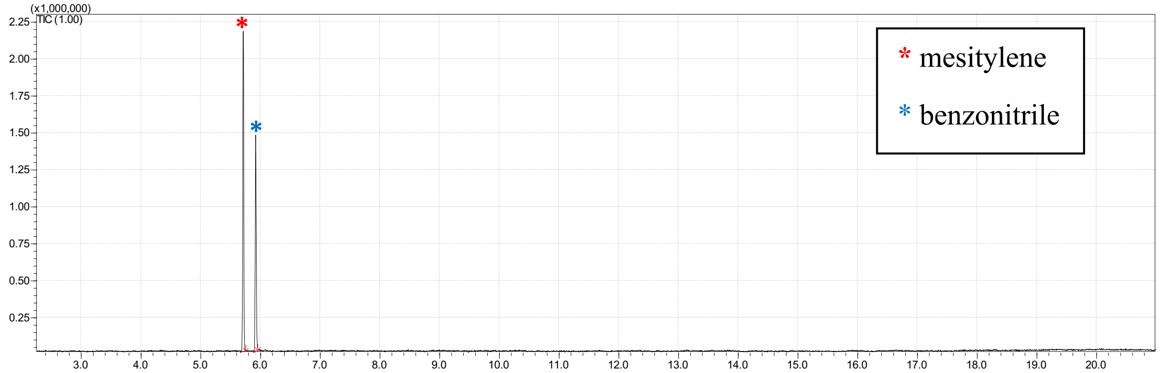

Figure S92. GC-MS trace of an equimolar solution of benzonitrile and mesitylene. Table below denoting peak identity, retention times, and areas of the peaks.

Table S43. Relative peak integration from Figure S92.

Compound	*RT*	*Start Time*	*End Time*	*Area*	*% Area*
mesitylene	5.712	5.67	5.753	2524349	58
benzonitrile	5.921	5.893	5.957	1813436	42

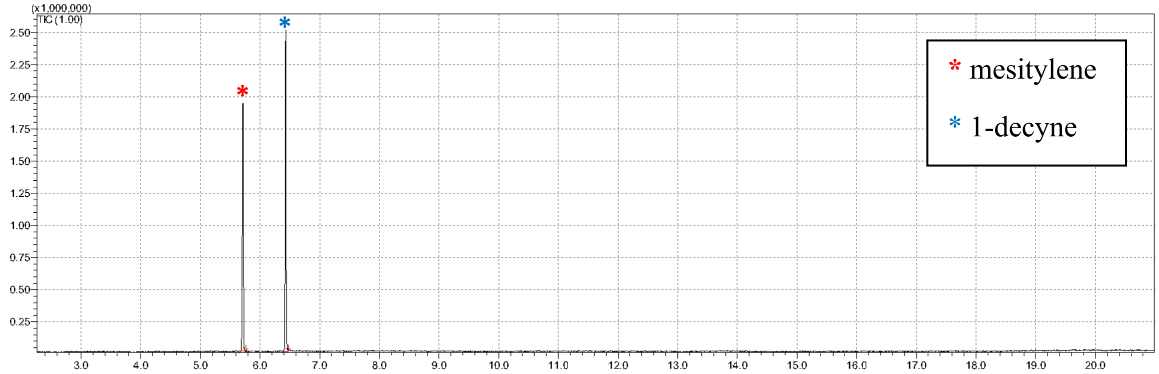

Figure S93. GC-MS trace of an equimolar solution of 1-decyne and mesitylene. Table below denoting peak identity, retention times, and areas of the peaks.

Table S44. Relative peak integration from Figure S93.

Compound	*RT*	*Start Time*	*End Time*	*Area*	*% Area*
mesitylene	5.712	5.67	5.76	2292488	47
1-decyne	6.43	6.393	6.477	2579968	53

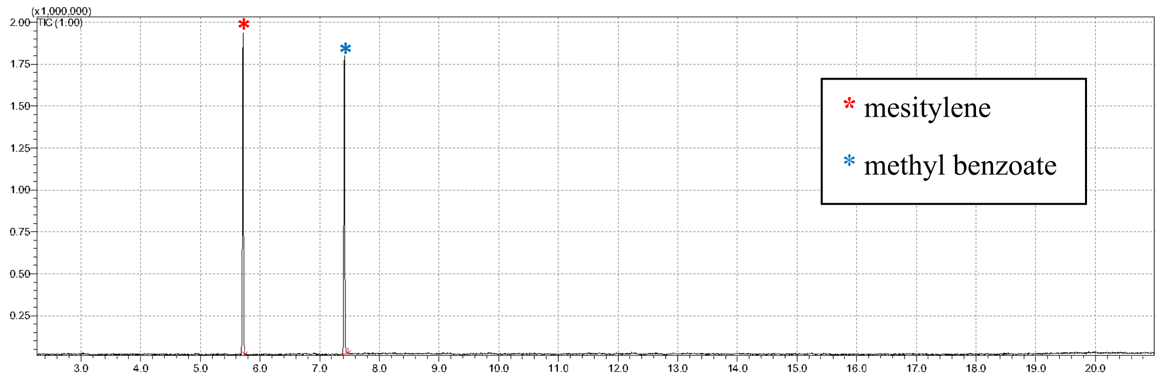

Figure S94. GC-MS trace of an equimolar solution of methylbenzoate and mesitylene. Table below denoting peak identity, retention times, and areas of the peaks.

Table S45. Relative peak integration from Figure S94.

Compound	*RT*	*Start Time*	*End Time*	*Area*	*% Area*
mesitylene	5.713	5.673	5.747	2217055	52
methylbenzoate	7.413	7.38	7.477	2015707	48

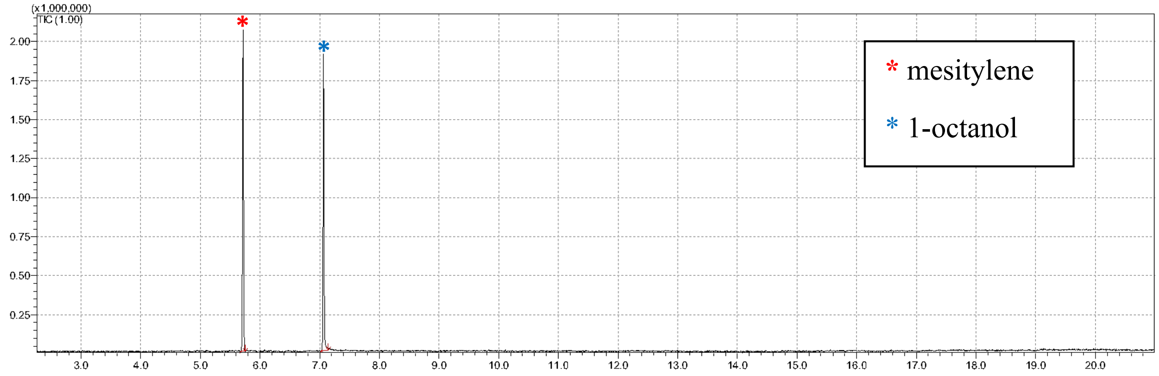

Figure S95. GC-MS trace of an equimolar solution of 1-octanol and mesitylene. Table below denoting peak identity, retention times, and areas of the peaks.

Table S46. Relative peak integration from Figure S95.

Compound	*RT*	*Start Time*	*End Time*	*Area*	*% Area*
mesitylene	5.712	5.67	5.76	2292488	47
1-octanol	6.43	6.393	6.477	2579968	53

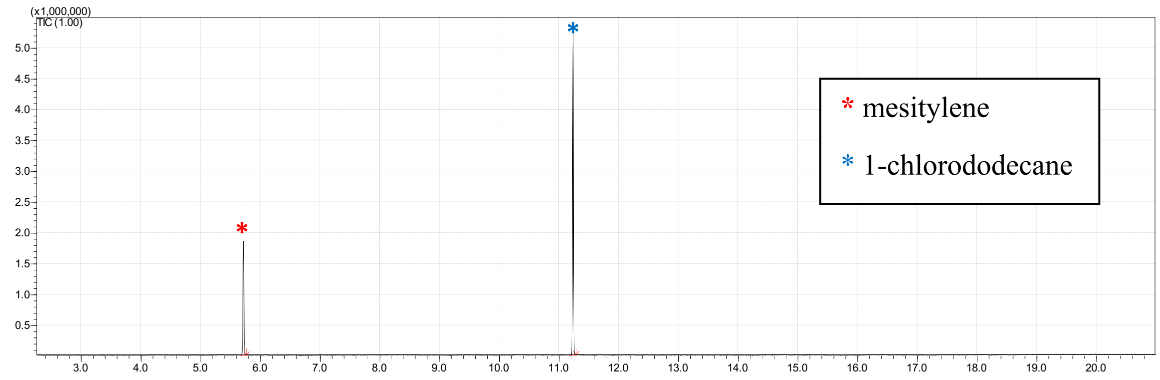

Figure S96. GC-MS trace of an equimolar solution of 1-chlorododecane and mesitylene. Table below denoting peak identity, retention times, and areas of the peaks.

Table S47. Relative peak integration from Figure S96.

Compound	*RT*	*Start Time*	*End Time*	*Area*	*% Area*
mesitylene	5.715	5.68	5.767	2164487	28
1-chlorododecane	11.233	11.19	11.283	5431888	72

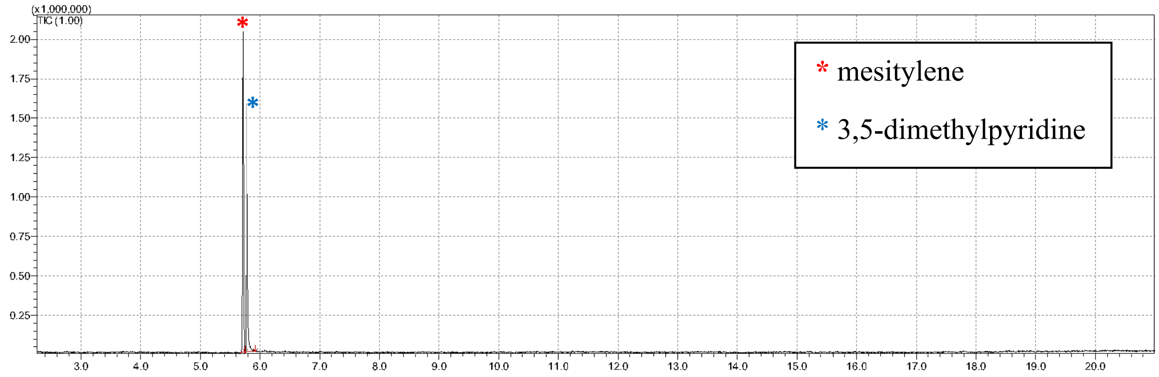

Figure S97. GC-MS trace of an equimolar solution of 3,5-dimethylpyridine and mesitylene. Table below denoting peak identity, retention times, and areas of the peaks.

Table S48. Relative peak integration from Figure S97.

Compound	*RT*	*Start Time*	*End Time*	*Area*	*% Area*
mesitylene	5.716	5.68	5.753	2358967	59
3,5-dimethylpyridine	5.776	5.753	5.923	1629776	41

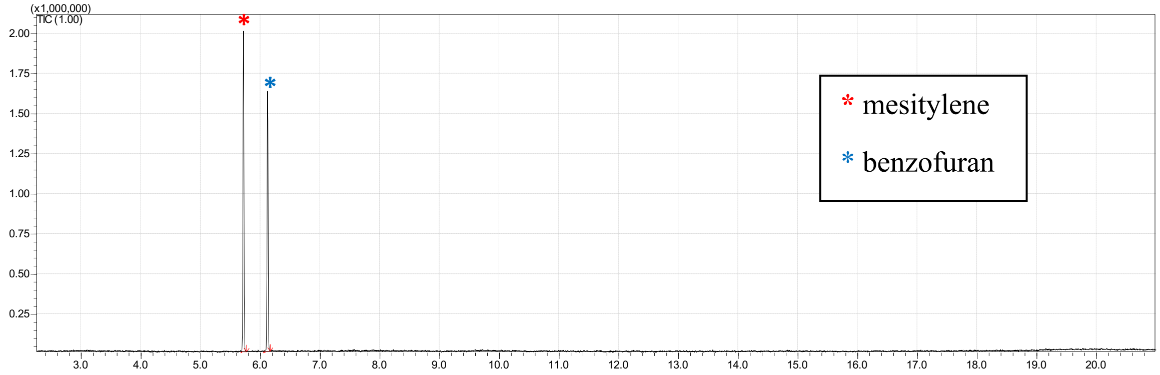

Figure S98. GC-MS trace of an equimolar solution of benzofuran and mesitylene. Table below denoting peak identity, retention times, and areas of the peaks.

Table S49. Relative peak integration from Figure S98.

Compound	*RT*	*Start Time*	*End Time*	*Area*	*% Area*
mesitylene	5.716	5.68	5.767	2345538	54
benzofuran	6.119	6.063	6.163	1991751	46

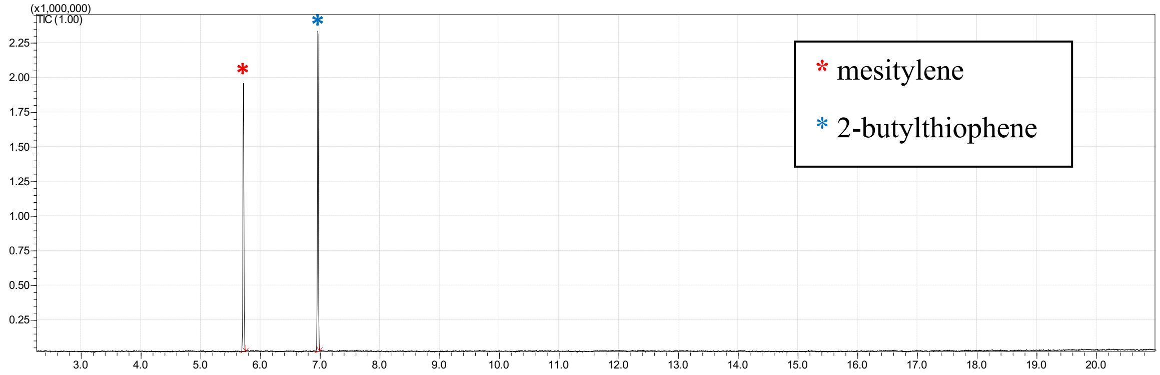

Figure S99. GC-MS trace of an equimolar solution of 2-butyl-thiophene and mesitylene. Table below denoting peak identity, retention times, and areas of the peaks.

Table S50. Relative peak integration from Figure S99.

Compound	*RT*	*Start Time*	*End Time*	*Area*	*% Area*
mesitylene	5.715	5.68	5.753	2281825	47
2-butylthiophene	6.961	6.93	6.993	2611383	53

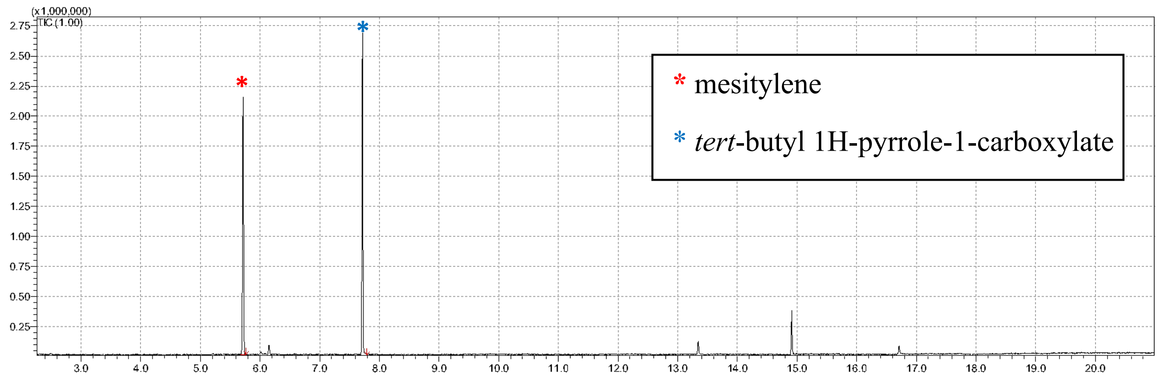

Figure S100. GC-MS trace of an equimolar solution of *tert*-butyl 1H-pyrrole-1-carboxylate and mesitylene. Table below denoting peak identity, retention times, and areas of the peaks.

Table S51. Relative peak integration from Figure S100.

Compound	*RT*	*Start Time*	*End Time*	*Area*	*% Area*
mesitylene	5.715	5.677	5.767	2534145	47
tert-*butyl 1H-pyrrole-1-carboxylate*	7.717	7.68	7.79	2892201	53

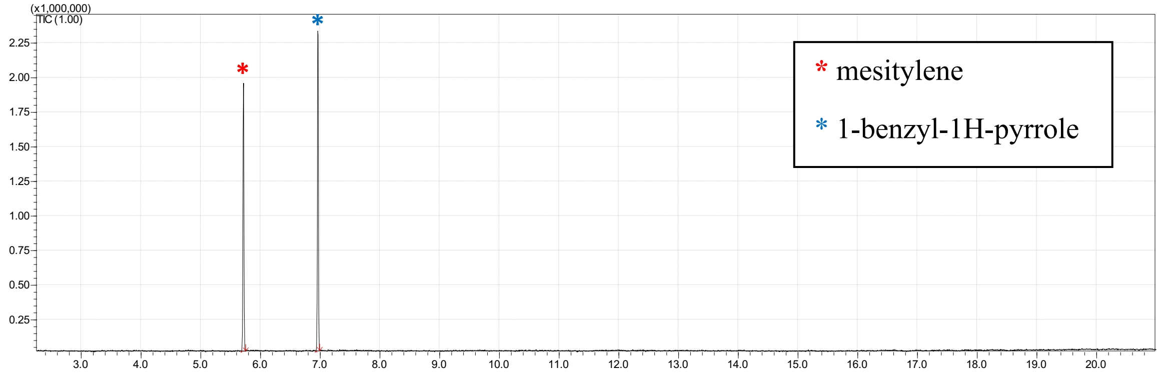

Figure S101. GC-MS trace of an equimolar solution of 1-benzyl-1H-pyrrole and mesitylene. Table below denoting peak identity, retention times, and areas of the peaks.

Table S52. Relative peak integration from Figure S101.

Compound	*RT*	*Start Time*	*End Time*	*Area*	*% Area*
mesitylene	5.715	5.683	5.747	2595155	50
1-benzyl-1H-pyrrole	10.203	10.17	10.25	2636399	50

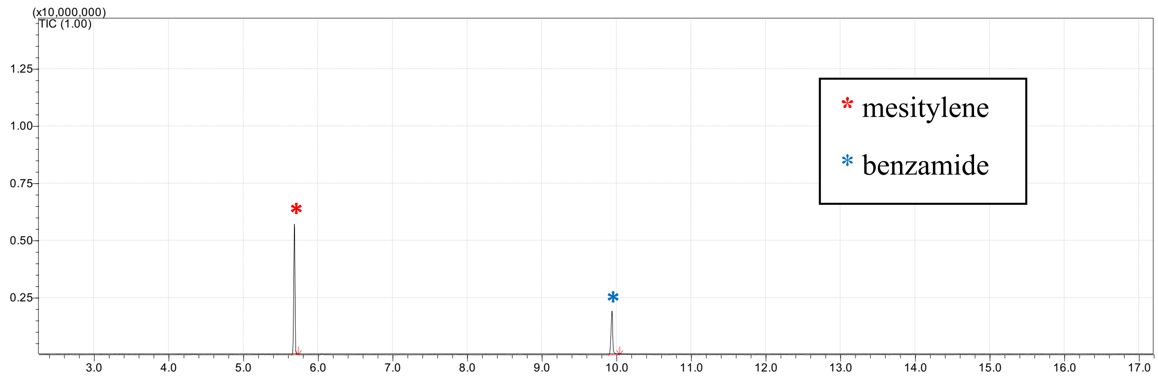

Figure S102. GC-MS trace of an equimolar solution of benzamide and mesitylene. Table below denoting peak identity, retention times, and areas of the peaks.

Table S53. Relative peak integration from Figure S102.

Compound	*RT*	*Start Time*	*End Time*	*Area*	*% Area*
mesitylene	5.681	5.643	5.733	6732062	68
benzamide	9.937	9.883	10.04	3233667	32

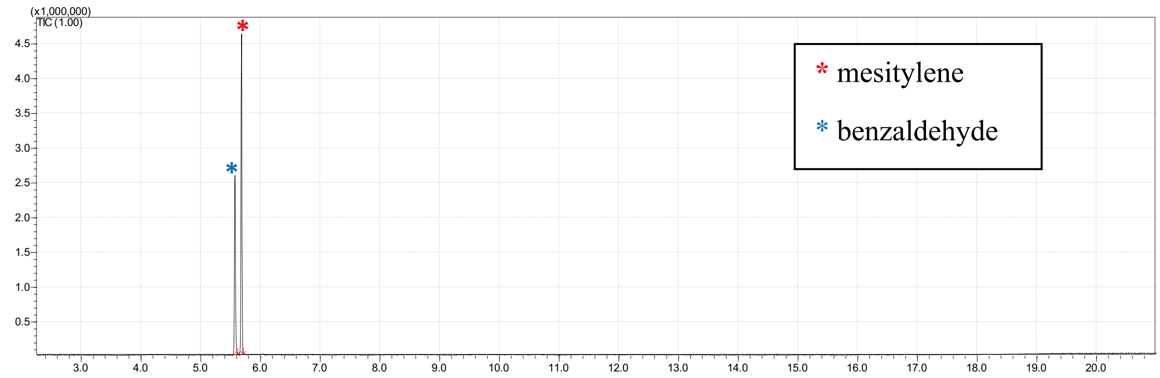

Figure S103. GC-MS trace of an equimolar solution of benzaldehyde and mesitylene. Table below denoting peak identity, retention times, and areas of the peaks.

Table S54. Relative peak integration from Figure S103.

Compound	*RT*	*Start Time*	*End Time*	*Area*	*% Area*
benzaldehyde	5.573	5.533	5.62	3315840	38
mesitylene	5.683	5.647	5.717	5360819	62

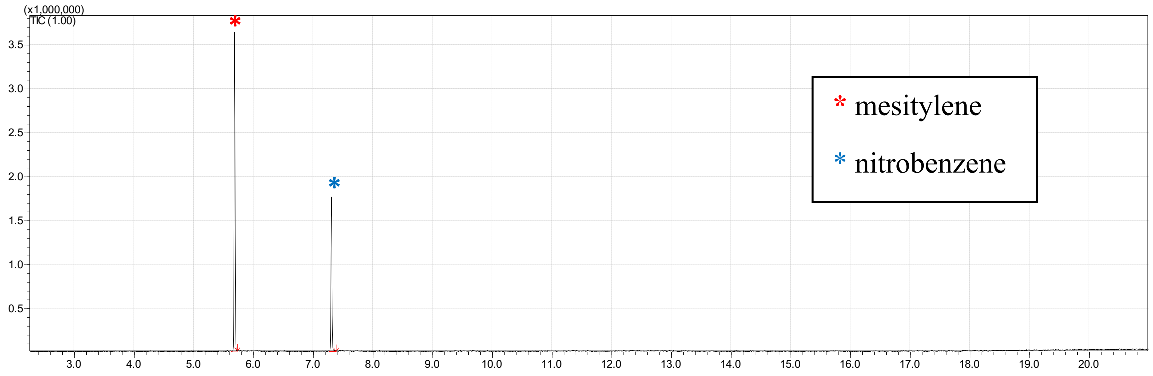

Figure S104. GC-MS trace of an equimolar solution of nitrobenzene and mesitylene. Table below denoting peak identity, retention times, and areas of the peaks.

Table S55. Relative peak integration from Figure S104.

Compound	RT	Start Time	End Time	Area	% Area
mesitylene	5.683	5.643	5.727	4191708	68
nitrobenzene	7.304	7.273	7.383	2012388	32

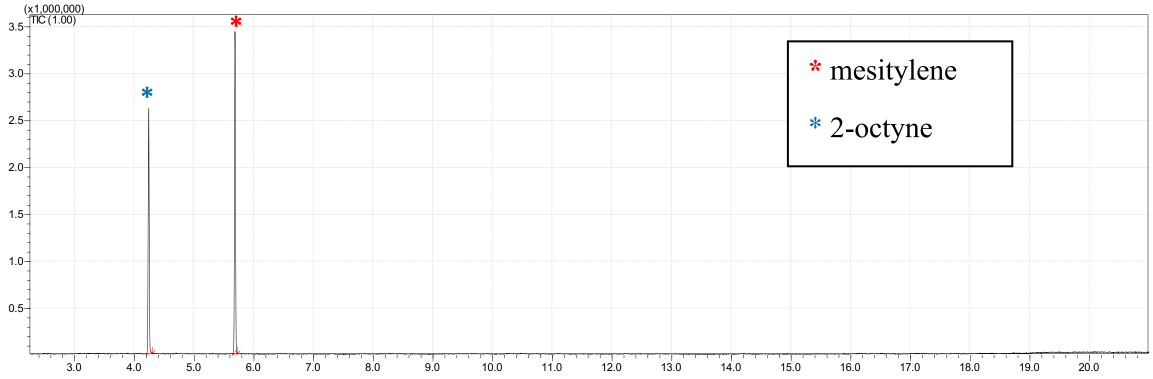

Figure 105. GC-MS trace of an equimolar solution of 2-octyne and mesitylene. Table below denoting peak identity, retention times, and areas of the peaks.

Table S56. Relative peak integration from Figure S105.

Compound	RT	Start Time	End Time	Area	% Area
2-octyne	4.239	4.203	4.303	3167279	44
mesitylene	5.684	5.643	5.727	4021869	56

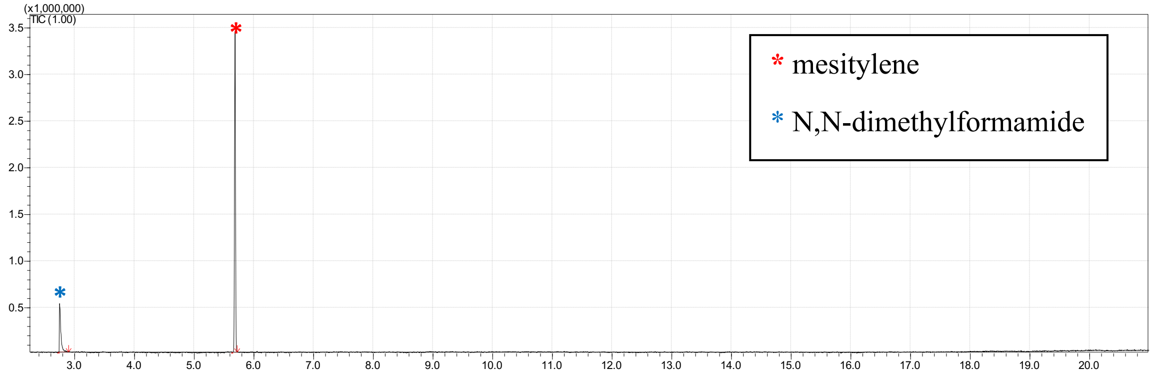

Figure S106. GC-MS trace of an equimolar solution of N,N-dimethylformamide and mesitylene. Table below denoting peak identity, retention times, and areas of the peaks.

Table S57. Relative peak integration from Figure S106.

Compound	*RT*	*Start Time*	*End Time*	*Area*	*% Area*
N,N-dimethylformamide	2.748	2.73	2.897	910594	18
mesitylene	5.685	5.64	5.72	4092222	82

Robustness Screening GC-MS Traces

Table S58: Robustness Screening Results

Entry	*Additive*	*% Conversion*	*% m.i.s.*	*E/Z Ratio*	*% Recovery*
1	chlorobenzene	91	91	1.5	96
2	aniline	3	63	1.4	88
3	benzonitrile	85	91	1.7	94
4	nitrobenzene	94	92	1.7	100
5	benzamide	10	92	1.2	51
6	methyl benzoate	89	92	1.5	98
7	benzaldehyde	97	92	1.5	93
8	benzofuran	89	92	1.1	98
9	1-chlorododecane	84	94	1.4	98
10	2-butylthiophene	73	91	1.3	94
11	3,5-dimethylpyridine	0	---	---	90
12	N,N-dimethyl-formamide	36	93	1.2	89
13	*tert*-butyl 1H-pyrrole-1-carboxylate	25	96	1.3	75
14	1-benzyl-1H-pyrrole	81	93	1.4	91
15	2-octyne	0	---	---	86
16	1-decyne	0	---	---	80
17	1-octanol	88	88	1.9	94

All additives were screened according to the general procedure described previously herein with 1-decene as the model substrate. % Conversion was determined with GC-MS by seeing how much 1-decene was converted to internal olefins. % Mono-isomerization selectivity (m.i.s.) was determined with GC-MS by seeing what percentage of the isomerized product was mono-isomerized. E/Z ratio was determined comparing the relative area of *E*-2-decene and *Z*-2-decene. % Recovery was determined using Equation S1.

Data for Table S58: Entry ***1***

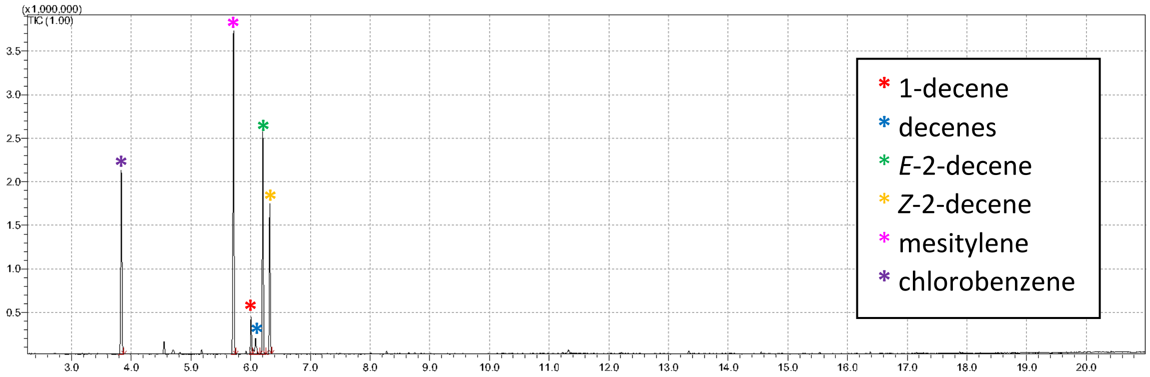

Figure S107. GC-MS Trace of Table S58: Entry 1; chlorobenzene.

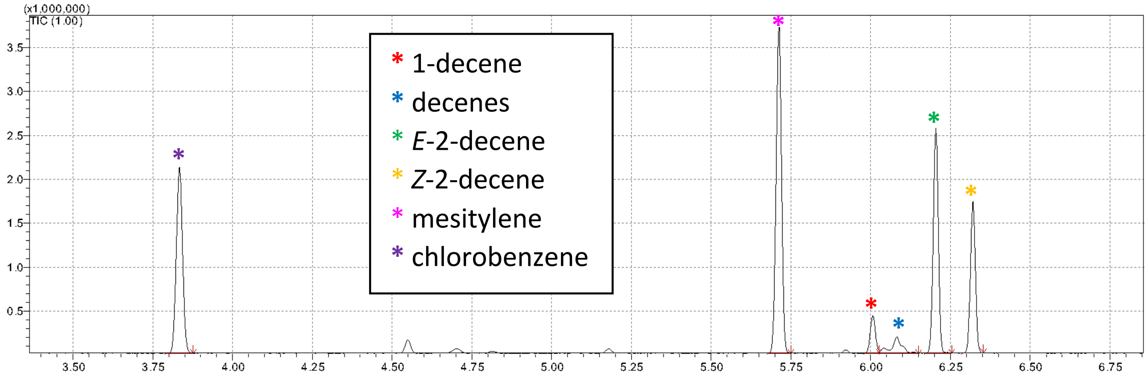

Figure S108. GC-MS Trace of Table S58: Entry 1; chlorobenzene (Expanded View). Table below denoting peak identity, retention times, and areas of the peaks. 0.25 mmol (35 µL) of mesitylene was added to the reaction just prior to GC-MS sampling.

Table S59. Relative peak integrations for Table S58: entry 1

Compound	*RT*	*Start Time*	*End Time*	*Area*	*% Area*
chlorobenzene	3.833	3.79	3.877	2889180	22
mesitylene (I.S.)	5.712	5.677	5.75	4448447	34
1-decene	6.006	5.98	6.027	493248	4
decenes	6.082	6.027	6.15	443664	3
E-2-decene	6.204	6.17	6.253	2839401	22
Z-2-decene	6.32	6.287	6.353	1864895	14

*Data for Table S58: Entry **2***

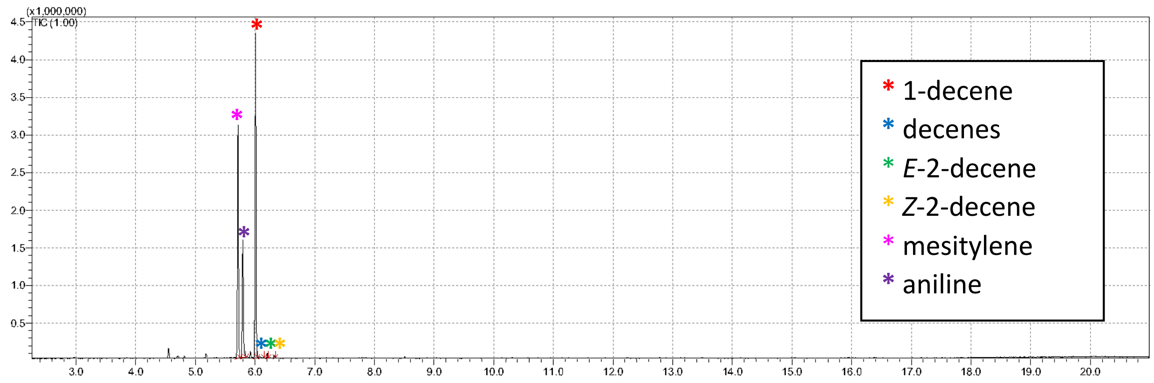

Figure S109. GC-MS Trace of Table S58: Entry 2; aniline.

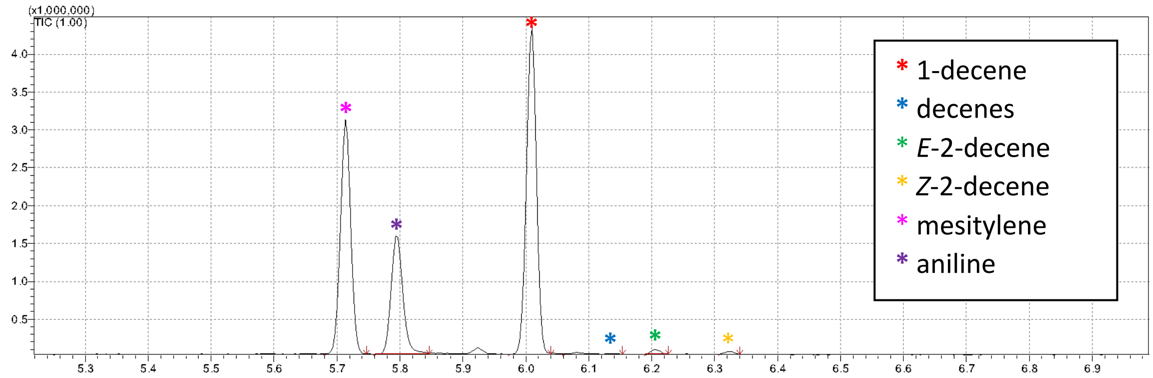

Figure S110. GC-MS Trace of Table S58: Entry 2; aniline (Expanded View). Table below denoting peak identity, retention times, and areas of the peaks. 0.25 mmol (35 µL) of mesitylene was added to the reaction just prior to GC-MS sampling.

Table S60. Relative peak integrations for Table S58: entry 2

Compound	*RT*	*Start Time*	*End Time*	*Area*	*% Area*
mesitylene (I.S.)	5.714	5.68	5.747	3535723	34
aniline	5.795	5.76	5.847	2038583	19
1-decene	6.009	5.973	6.04	4788276	46
decenes	6.08	6.06	6.153	60080	0.6
E-2-decene	6.205	6.19	6.227	59011	0.6
Z-2-decene	6.324	6.307	6.34	41434	0.4

*Data for Table S58: Entry **3***

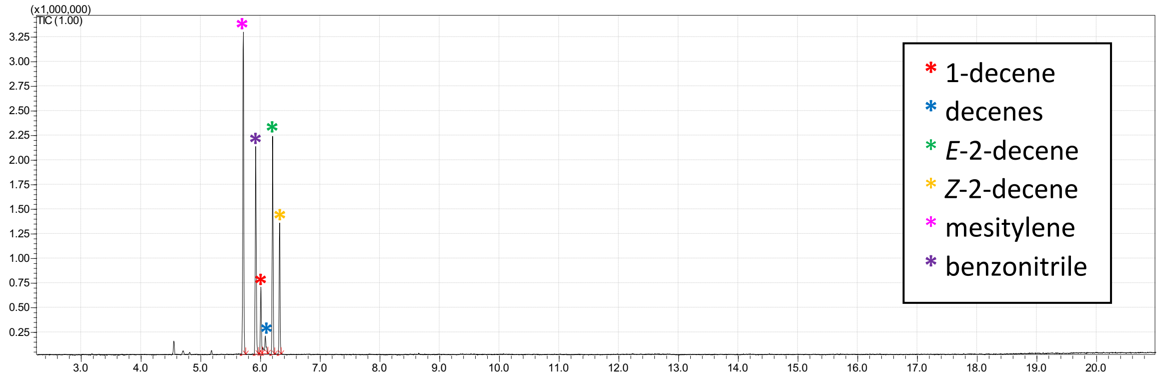

Figure S111. GC-MS Trace of Table S58: Entry 3; benzonitrile.

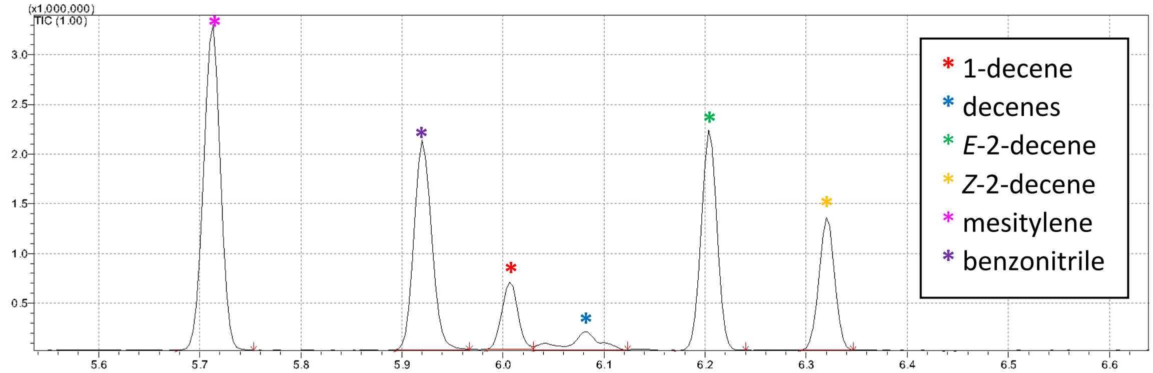

Figure S112. GC-MS Trace of Table S58: Entry 3; benzonitrile (Expanded View). Table below denoting peak identity, retention times, and areas of the peaks. 0.25 mmol (35 μL) of mesitylene was added to the reaction just prior to GC-MS sampling.

Table S61. Relative peak integrations for Table S58: entry 3

Compound	*RT*	*Start Time*	*End Time*	*Area*	*% Area*
mesitylene (I.S.)	5.713	5.677	5.753	3828449	34
benzonitrile	5.92	5.893	5.967	2582647	23
1-decene	6.007	5.983	6.03	754160	7
decenes	6.082	6.03	6.123	372483	3
E-2-decene	6.204	6.17	6.24	2411275	21
Z-2-decene	6.321	6.293	6.347	1447138	13

*Data for Table S58: Entry **4***

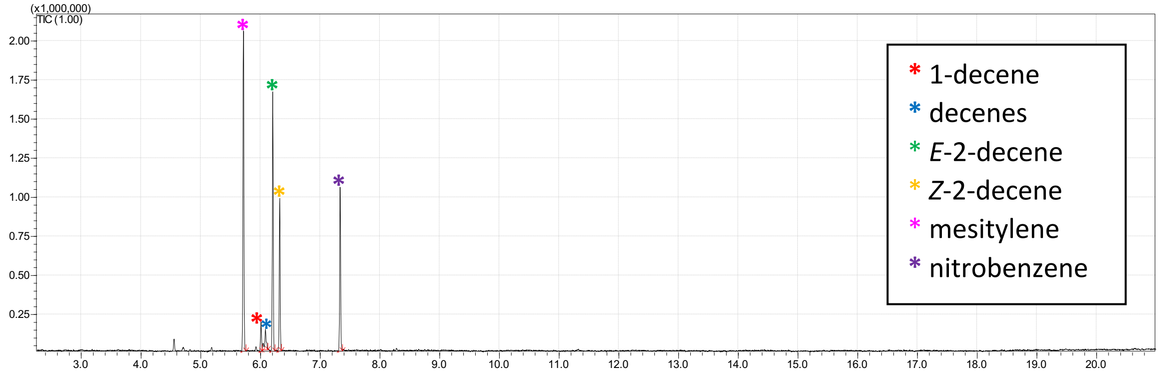

Figure S113. GC-MS Trace of Table S58: Entry 4; nitrobenzene.

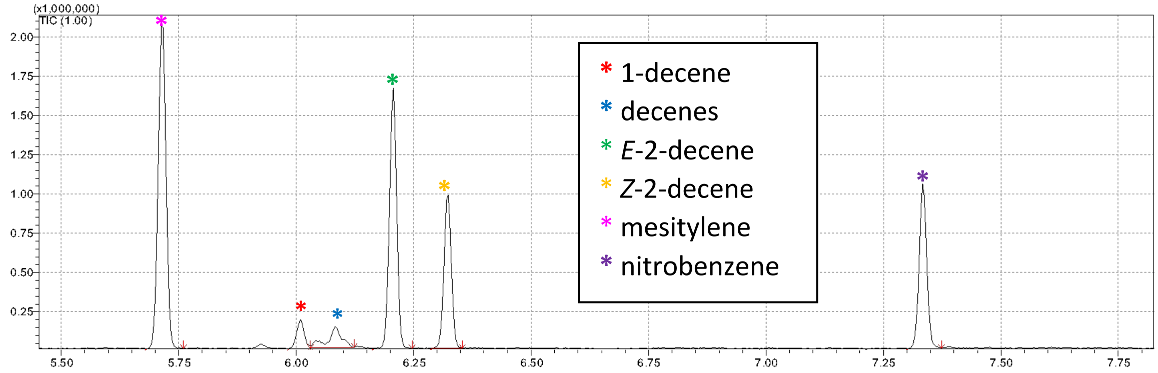

Figure S114. GC-MS Trace of Table S58: Entry 4; nitrobenzene. Table below denoting peak identity, retention times, and areas of the peaks. 0.25 mmol (35 µL) of mesitylene was added to the reaction just prior to GC-MS sampling.

Table S62. Relative peak integrations for Table S58: entry 4

Compound	*RT*	*Start Time*	*End Time*	*Area*	*% Area*
mesitylene (I.S.)	5.715	5.68	5.76	2425374	35
1-decene	6.01	5.983	6.03	211547	3
decenes	6.084	6.03	6.123	257335	4
E-2-decene	6.206	6.163	6.247	1794752	26
Z-2-decene	6.322	6.287	6.353	1075544	15
nitrobenzene	7.334	7.3	7.373	1181685	17

*Data for Table S58: Entry **5***

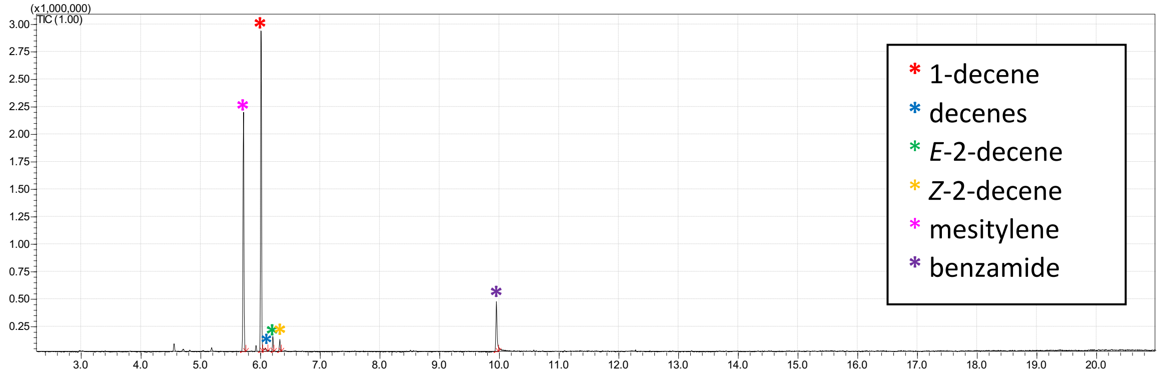

Figure S115. GC-MS Trace of Table S58: Entry 5; benzamide.

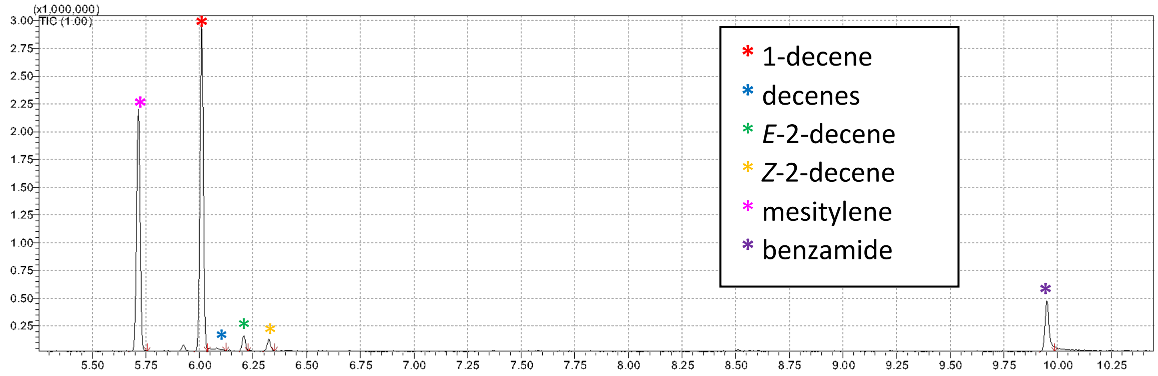

Figure S116. GC-MS Trace of Table S58: Entry 5; benzamide. Table below denoting peak identity, retention times, and areas of the peaks. 0.25 mmol (35 µL) of mesitylene was added to the reaction just prior to GC-MS sampling.

Table S63. Relative peak integrations for Table S58: entry 5

Compound	*RT*	*Start Time*	*End Time*	*Area*	*% Area*
mesitylene (I.S.)	5.716	5.67	5.757	2523573	38
1-decene	6.011	5.977	6.037	3207341	48
decenes	6.08	6.037	6.123	87216	1
E-2-decene	6.208	6.187	6.227	156080	2

Z-2-decene	6.324	6.297	6.35	126939	2
benzamide	9.95	9.917	9.987	621929	9

*Data for Table S58: Entry **6***

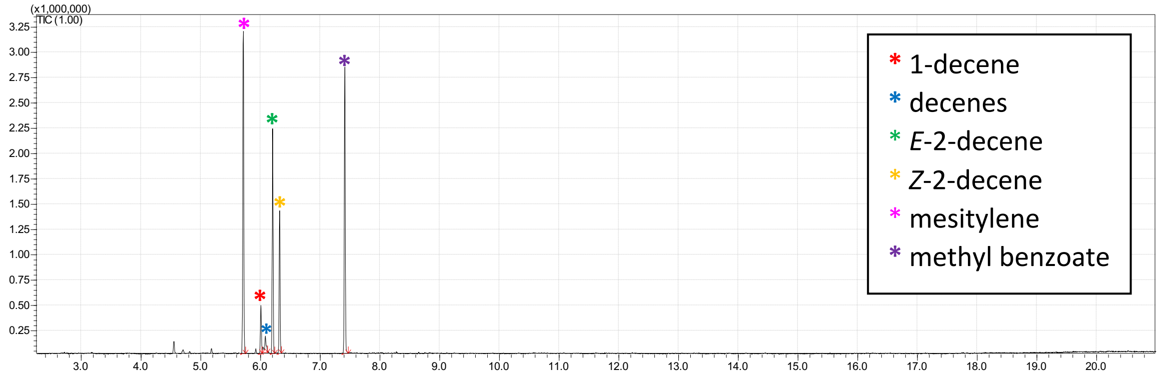

Figure S117. GC-MS Trace of Table S58: Entry 6; methyl benzoate.

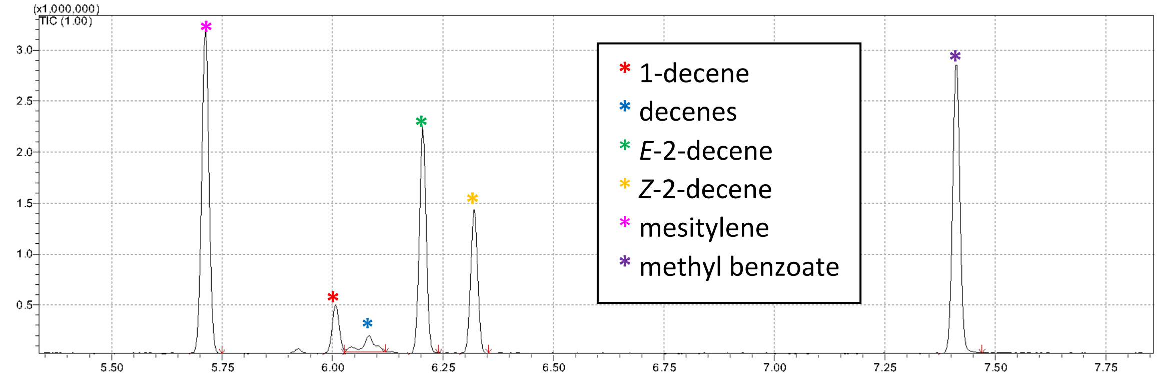

Figure S118. GC-MS Trace of Table S58: Entry 6; methyl benzoate (Expanded View). Table below denoting peak identity, retention times, and areas of the peaks. 0.25 mmol (35 μL) of mesitylene was added to the reaction just prior to GC-MS sampling.

Table S64. Relative peak integrations for Table S58: entry 6

Compound	*RT*	*Start Time*	*End Time*	*Area*	*% Area*
mesitylene (I.S.)	5.712	5.677	5.75	3699105	31
1-decene	6.007	5.98	6.027	529752	4

decenes	6.082	6.027	6.12	333777	3
E-2-decene	6.204	6.173	6.24	2391122	20
Z-2-decene	6.321	6.29	6.353	1559409	13
methyl benzoate	7.412	7.373	7.47	3303225	28

Data for Table S58: Entry 7

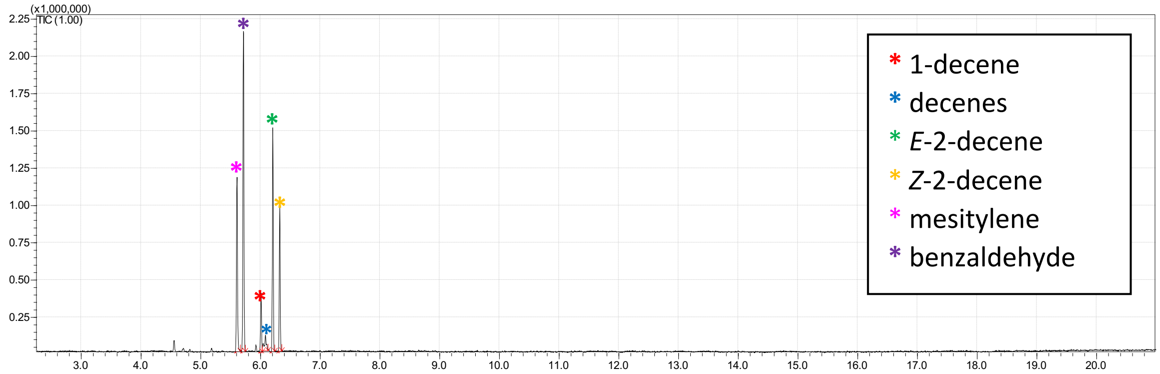

Figure S119. GC-MS Trace of Table S58: Entry 7; benzaldehyde.

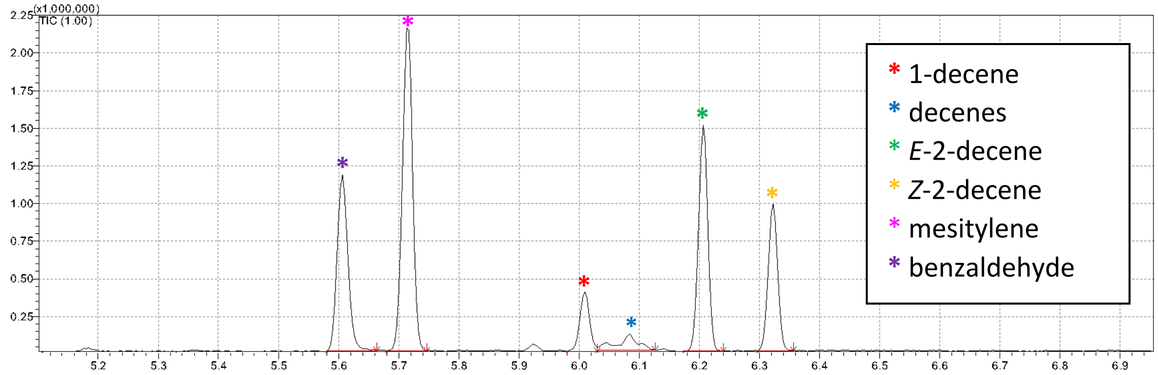

Figure S120. GC-MS Trace of Table S58: Entry 7; benzaldehyde (Expanded View). Table below denoting peak identity, retention times, and areas of the peaks. 0.25 mmol (35 µL) of mesitylene was added to the reaction just prior to GC-MS sampling.

Table S65. Relative peak integrations for Table S58: entry 7

Compound	*RT*	*Start Time*	*End Time*	*Area*	*% Area*

benzaldehyde	5.606	5.58	5.663	1475415	20
mesitylene (I.S.)	5.715	5.68	5.747	2575269	35
1-decene	6.01	5.98	6.03	456208	6
decenes	6.084	6.03	6.127	248647	3
E-2-decene	6.207	6.173	6.24	1621648	22
Z-2-decene	6.323	6.297	6.357	1078931	14

*Data for Table S58: Entry **8***

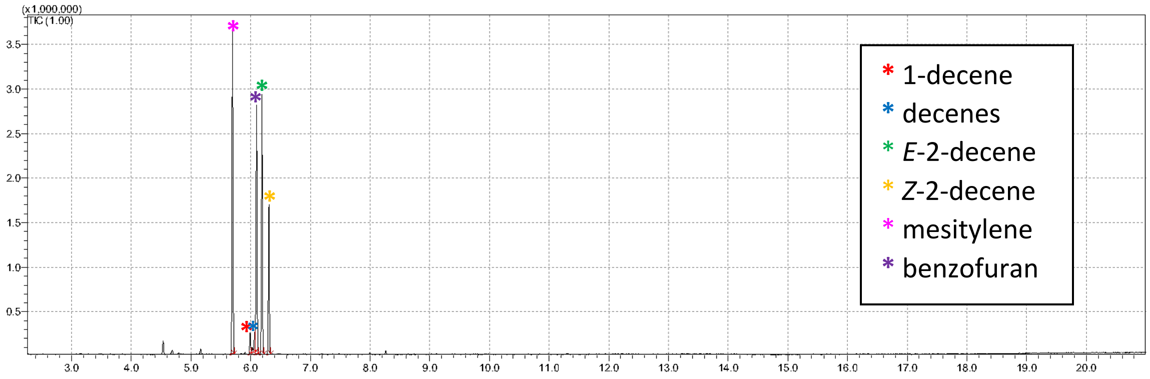

Figure S121. GC-MS Trace of Table S58: Entry 8; benzofuran.

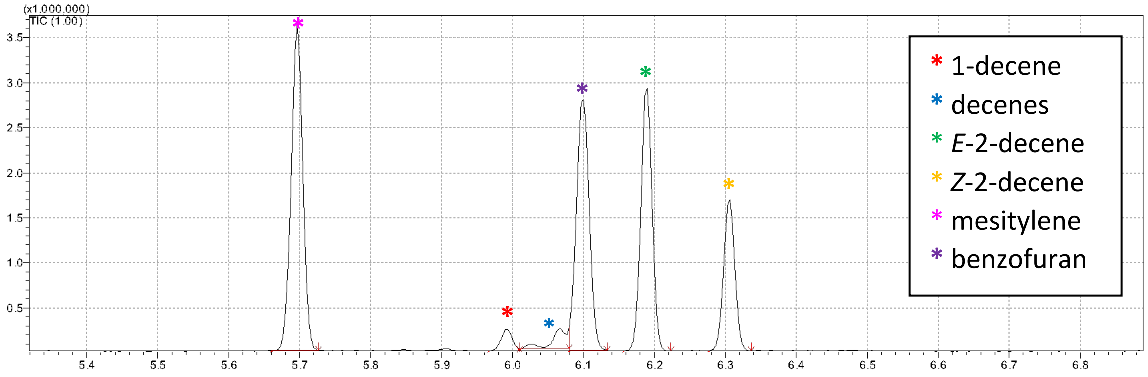

Figure S122. GC-MS Trace of Table S58: Entry 8; benzofuran (Expanded View). Table below denoting peak identity, retention times, and areas of the peaks. 0.25 mmol (35 μL) of mesitylene was added to the reaction just prior to GC-MS sampling.

Table S66. Relative peak integrations for Table S58: entry 8

Compound	RT	Start Time	End Time	Area	% Area
mesitylene (I.S.)	5.696	5.66	5.727	4136953	31
1-decene	5.992	5.967	6.01	267621	2
decenes	6.08	6.01	6.08	366128	3
benzofuran	6.099	6.08	6.133	3496582	26
E-2-decene	6.189	6.157	6.223	3209991	24
Z-2-decene	6.305	6.277	6.337	1856206	14

*Data for Table S58: Entry **9***

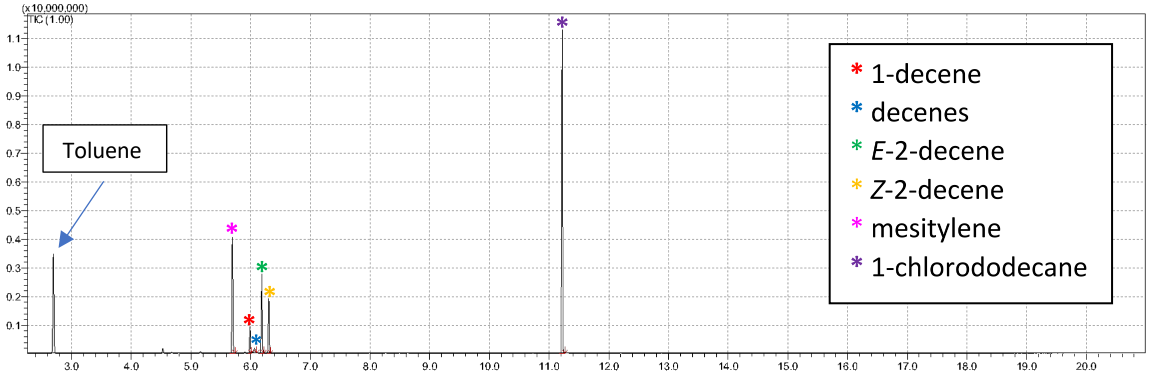

Figure S123. GC-MS Trace of Table S58: Entry 9; 1-chlorododecane.

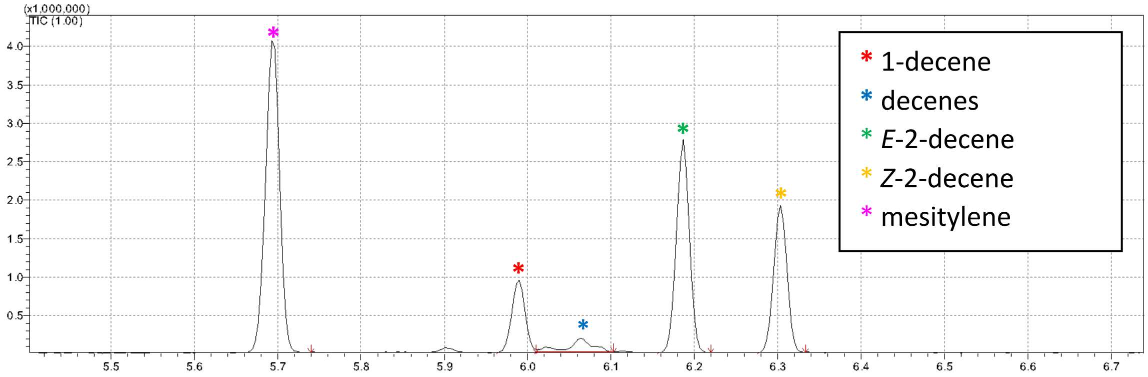

Figure S124. GC-MS Trace of Table S58: Entry 9; 1-chlorododecane (Expanded View). Table below denoting peak identity, retention times, and areas of the peaks. 0.25 mmol (35 μL) of mesitylene was added to the reaction just prior to GC-MS sampling.

Table S67. Relative peak integrations for Table S58: entry 9

Compound	*RT*	*Start Time*	*End Time*	*Area*	*% Area*
mesitylene (I.S.)	5.695	5.65	5.74	4851651	21
1-decene	5.989	5.963	6.01	1060864	5
decenes	6.064	6.01	6.103	350110	2
E-2-decene	6.186	6.157	6.22	3020972	13
Z-2-decene	6.303	6.277	6.333	2139899	9
1-chlorododecane	11.219	11.177	11.263	11968177	51

*Data for Table S58: Entry **10***

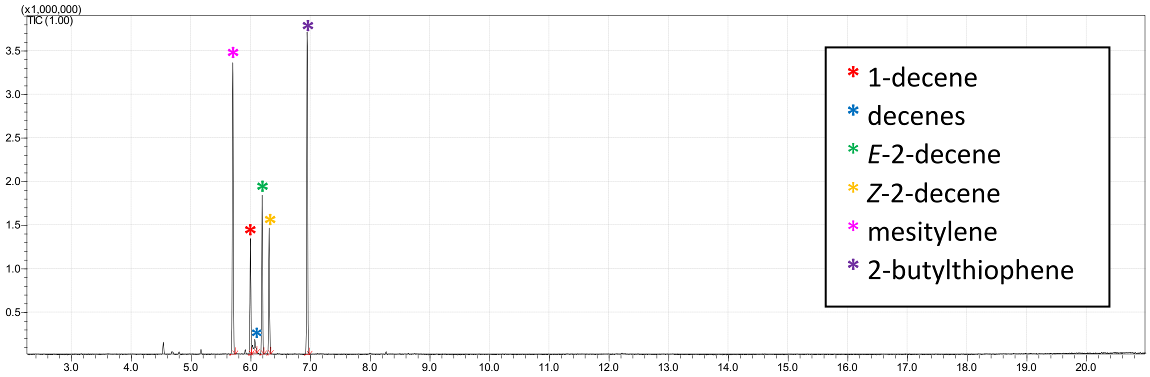

Figure S125. GC-MS Trace of Table S58: Entry 10; 2-butylthiophene.

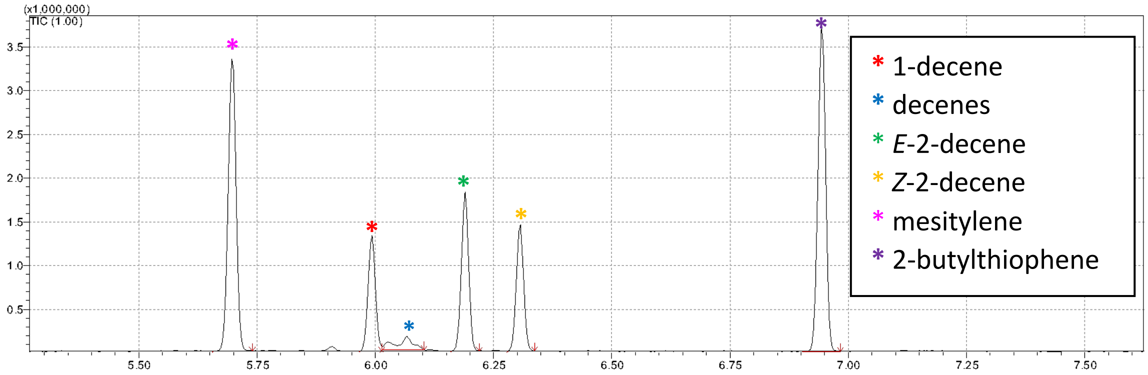

Figure S126. GC-MS Trace of Table S58: Entry 10; 2-butylthiophene (Expanded View). Table below denoting peak identity, retention times, and areas of the peaks. 0.25 mmol (35 μL) of mesitylene was added to the reaction just prior to GC-MS sampling.

Table S68. Relative peak integrations for Table S58: entry 10

Compound	*RT*	*Start Time*	*End Time*	*Area*	*% Area*
mesitylene (I.S.)	5.697	5.657	5.74	3940652	29
1-decene	5.993	5.967	6.013	1444130	11
decenes	6.067	6.013	6.103	373856	3
E-2-decene	6.19	6.16	6.22	1942876	14
Z-2-decene	6.306	6.28	6.337	1554787	12
2-butylthiophene	6.944	6.903	6.983	4233741	31

*Data for Table S58: Entry **11***

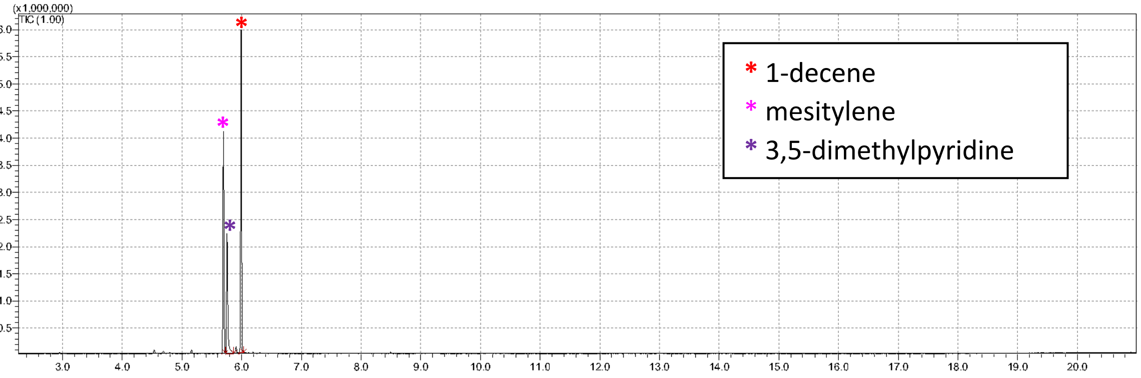

Figure S127. GC-MS Trace of Table S58: Entry 11; 3,5-dimethylpyridine.

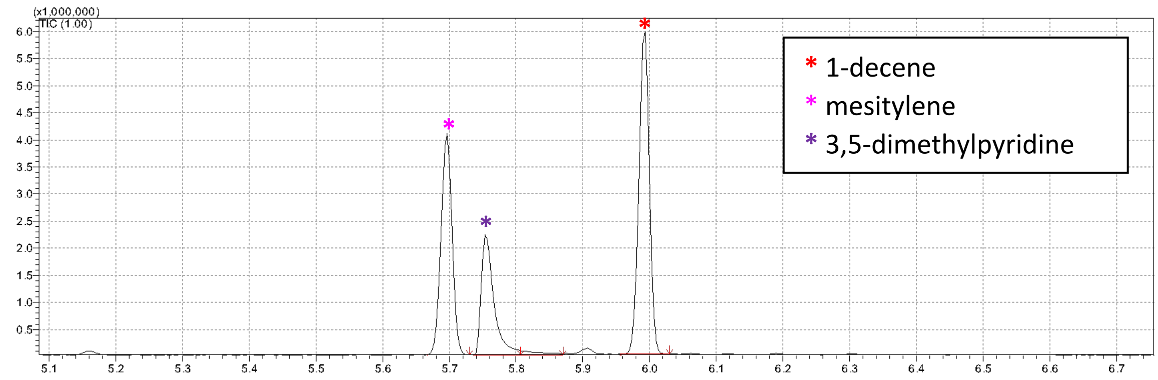

Figure S128. GC-MS Trace of Table S58: Entry 11; 3,5-dimethylpyridine (Expanded View). Table below denoting peak identity, retention times, and areas of the peaks. 0.25 mmol (35 μL) of mesitylene was added to the reaction just prior to GC-MS sampling.

Table S69. Relative peak integrations for Table S58: entry 11

Compound	*RT*	*Start Time*	*End Time*	*Area*	*% Area*
mesitylene (I.S.)	5.696	5.667	5.73	4713362	33
3,5-dimethylpyridine	5.754	5.737	5.87	2925876	22
1-decene	5.992	5.957	6.03	6515386	46

*Data for Table S58: Entry **12***

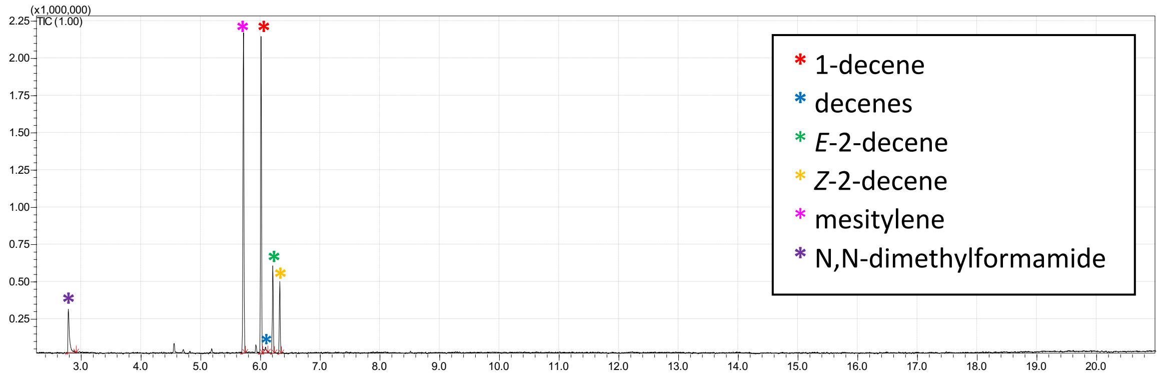

Figure S129. GC-MS Trace of Table S58: Entry 12; *N*,*N*-dimethylformamide.

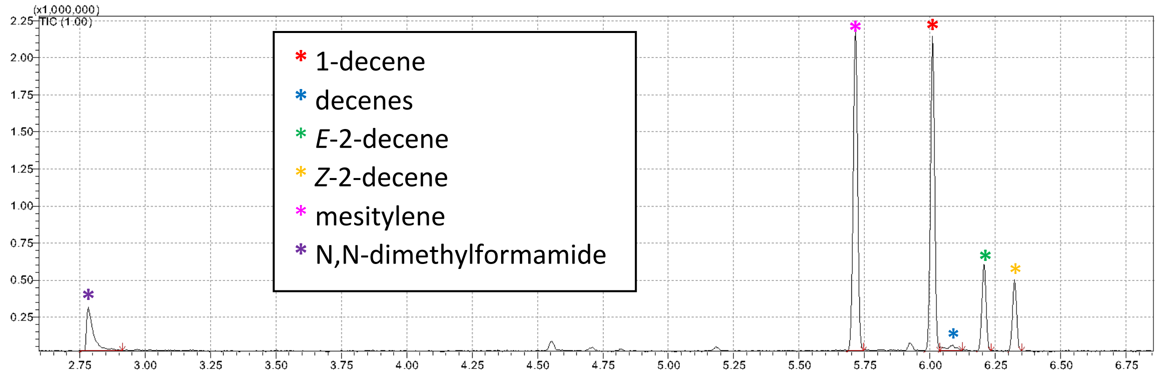

Figure S130. GC-MS Trace of Table S58: Entry 12; *N*,*N*-dimethylformamide (Expanded View). Table below denoting peak identity, retention times, and areas of the peaks. 0.25 mmol (35 μL) of mesitylene was added to the reaction just prior to GC-MS sampling.

Table S70. Relative peak integrations for Table S58: entry 12

Compound	*RT*	*Start Time*	*End Time*	*Area*	*% Area*
N,N-dimethylformamide	2.783	2.753	2.913	506769	8
mesitylene (I.S.)	5.715	5.683	5.747	2566606	38
1-decene	6.01	5.98	6.037	2327455	35
decenes	6.086	6.037	6.123	93372	1
E-2-decene	6.207	6.18	6.233	656648	10
Z-2-decene	6.323	6.3	6.35	532919	8

*Data for Table S58: Entry **13***

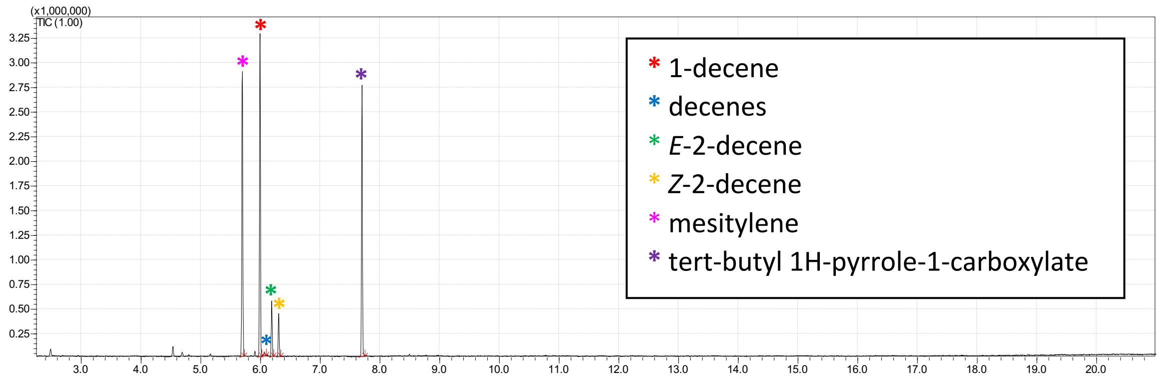

Figure S131. GC-MS Trace of Table S58: Entry 13; *tert*-butyl 1H-pyrrole-1-carboxylate.

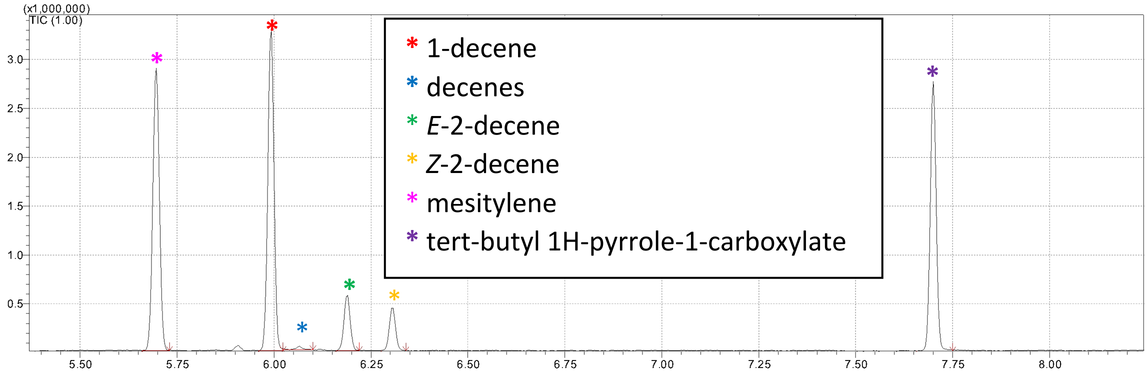

Figure S132. GC-MS Trace of Table S58: Entry 13; *tert*-butyl 1H-pyrrole-1-carboxylate. Table below denoting peak identity, retention times, and areas of the peaks. 0.25 mmol (35 μL) of mesitylene was added to the reaction just prior to GC-MS sampling.

Table S71. Relative peak integrations for Table S58: entry 13

Compound	*RT*	*Start Time*	*End Time*	*Area*	*% Area*
mesitylene (I.S.)	5.696	5.66	5.73	3430678	31
1-decene	5.992	5.96	6.023	3664434	33
decenes	6.066	6.023	6.1	51538	0
E-2-decene	6.189	6.16	6.22	640202	6
Z-2-decene	6.305	6.277	6.34	507851	5
tert-butyl 1H-pyrrole-1-carboxylate	7.7	7.657	7.75	2953506	26

*Data for Table S58: Entry **14***

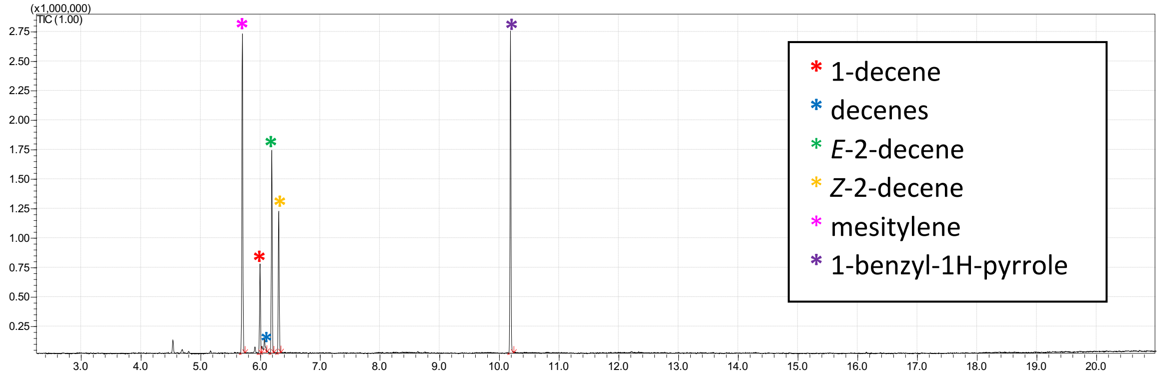

Figure S133. GC-MS Trace of Table S58: Entry 14; 1-benzyl-1H-pyrrole.

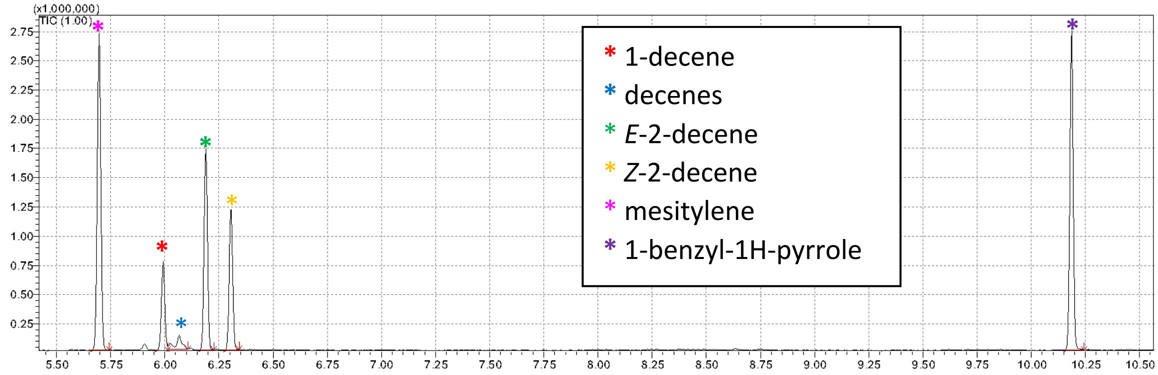

Figure S134. GC-MS Trace of Table S58: Entry 14; 1-benzyl-1H-pyrrole. Table below denoting peak identity, retention times, and areas of the peaks. 0.25 mmol (35 µL) of mesitylene was added to the reaction just prior to GC-MS sampling.

Table S72. Relative peak integrations for Table S58: entry 14

Compound	*RT*	*Start Time*	*End Time*	*Area*	*% Area*
mesitylene (I.S.)	5.697	5.66	5.743	3157236	30
1-decene	5.993	5.96	6.013	832027	8
decenes	6.066	6.013	6.107	254824	2
E-2-decene	6.189	6.16	6.227	1917210	18
Z-2-decene	6.305	6.277	6.343	1360051	13
1-benzyl-1H-pyrrole	10.186	10.153	10.243	2920756	28

*Data for Table S58: Entry **15***

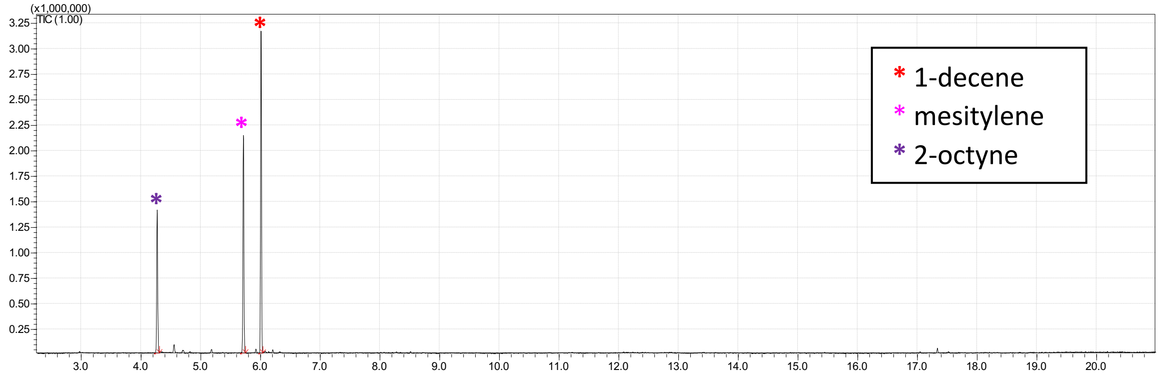

Figure S135. GC-MS Trace of Table S58: Entry 15; 2-octyne.

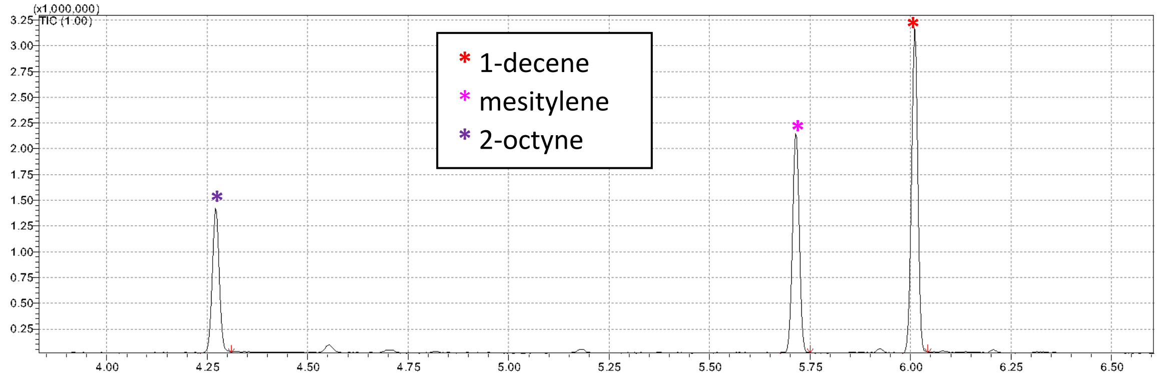

Figure S136. GC-MS Trace of Table S58: Entry 15; 2-octyne (Expanded View). Table below denoting peak identity, retention times, and areas of the peaks. 0.25 mmol (35 µL) of mesitylene was added to the reaction just prior to GC-MS sampling.

Table S73. Relative peak integrations for Table S58: entry 15

Compound	*RT*	*Start Time*	*End Time*	*Area*	*% Area*
2-octyne	4.271	4.233	4.31	1702884	22
mesitylene (I.S.)	5.715	5.677	5.75	2505261	33
1-decene	6.01	5.98	6.043	3438058	45

*Data for Table S58: Entry **16***

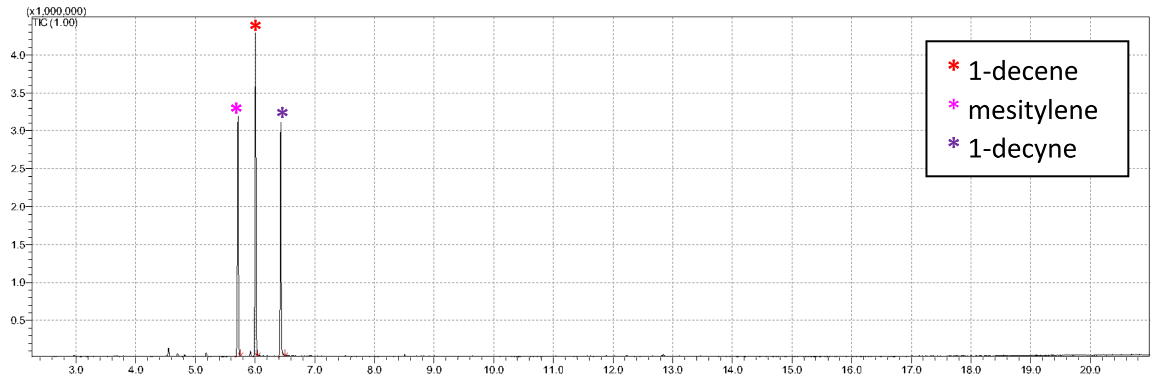

Figure S137. GC-MS Trace of Table S58: Entry 16; 1-decyne.

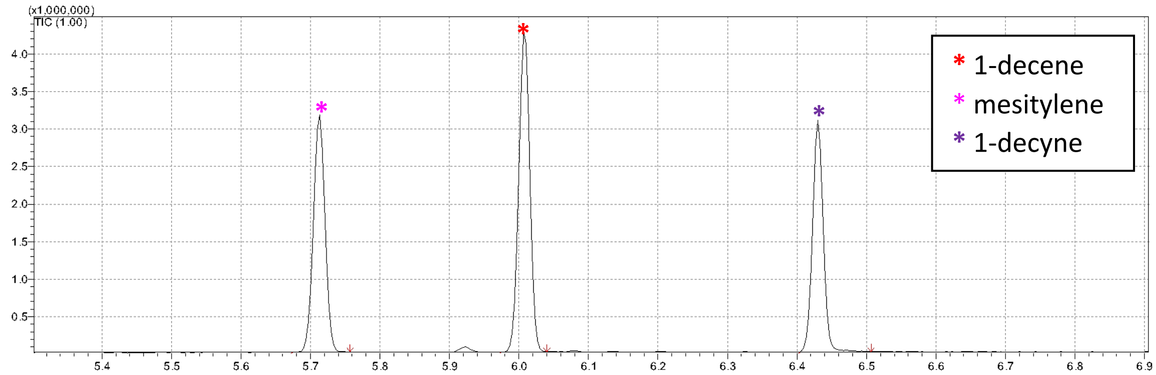

Figure S138. GC-MS Trace of Table S58: Entry 16; 1-decyne (Expanded View). Table below denoting peak identity, retention times, and areas of the peaks. 0.25 mmol (35 µL) of mesitylene was added to the reaction just prior to GC-MS sampling.

Table S74. Relative peak integrations for Table S58: entry 16

Compound	*RT*	*Start Time*	*End Time*	*Area*	*% Area*
mesitylene (I.S.)	5.713	5.673	5.757	3685457	31
1-decene	6.008	5.973	6.04	4751519	40
1-decyne	6.43	6.403	6.507	3312185	28

*Data for Table S68: Entry **17***

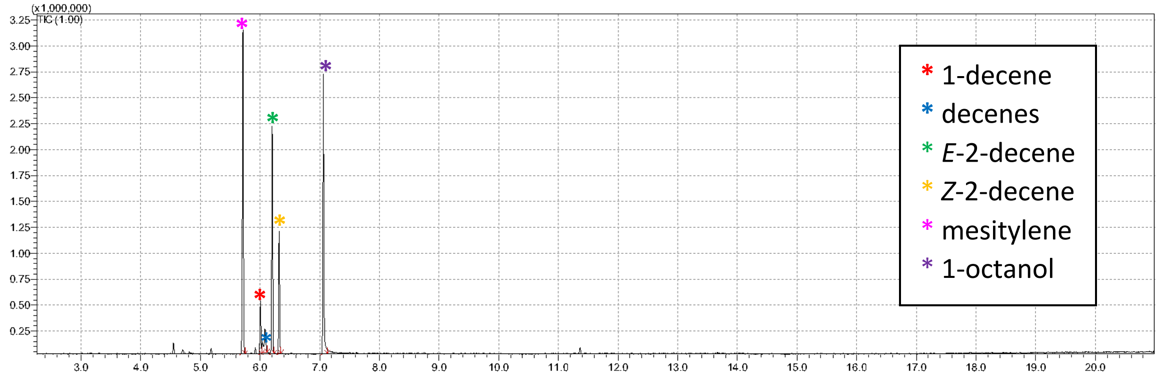

Figure S139. GC-MS Trace of Table S58: Entry 17; 1-octanol.

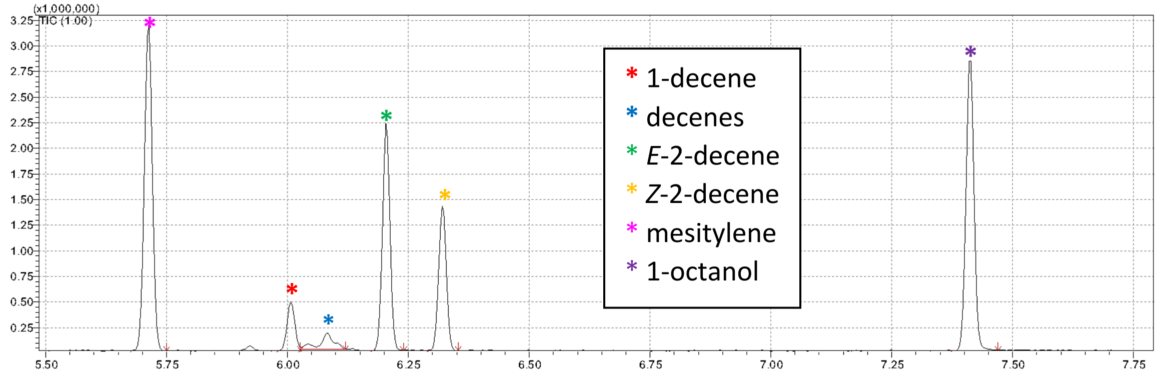

Figure S140. GC-MS Trace of Table S58: Entry 17; 1-octanol (Expanded View). Table below denoting peak identity, retention times, and areas of the peaks. 0.25 mmol (35 µL) of mesitylene was added to the reaction just prior to GC-MS sampling.

Table S75. Relative peak integrations for Table S58: entry 17

Compound	*RT*	*Start Time*	*End Time*	*Area*	*% Area*
mesitylene (I.S.)	5.712	5.67	5.75	3686031	32
1-decene	6.007	5.98	6.027	577557	5
decenes	6.081	6.027	6.12	498718	4
E-2-decene	6.204	6.167	6.237	2418598	21
Z-2-decene	6.32	6.287	6.35	1295748	11
1-octanol	7.061	7.03	7.127	3186907	27

SEM Imaging of Pd Particles

A Thermo Scientific Phenom XL SEM was used to collect the following images. Samples were prepared by depositing a slurry of freshly made particles on a SEM peg with copper tape. After solvent evaporated, the sample was washed with CH_2Cl_2 three times then dried under high vacuum overnight.

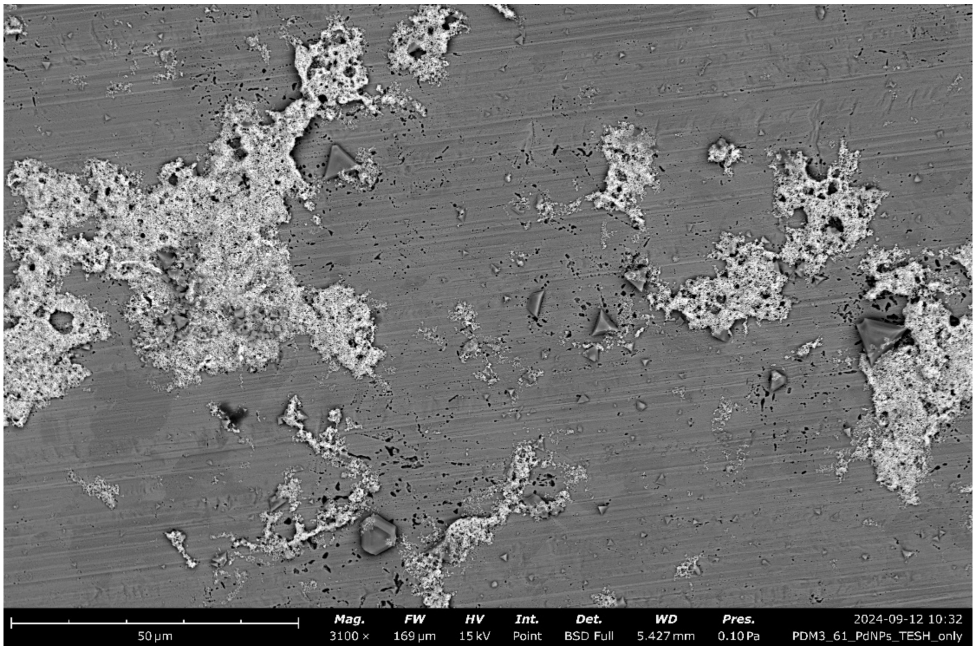

Figure S141. No $B(C_6F_5)_3$ used when reducing Pd(cod)Cl_2 with Et_3Si-H after 2 hours.

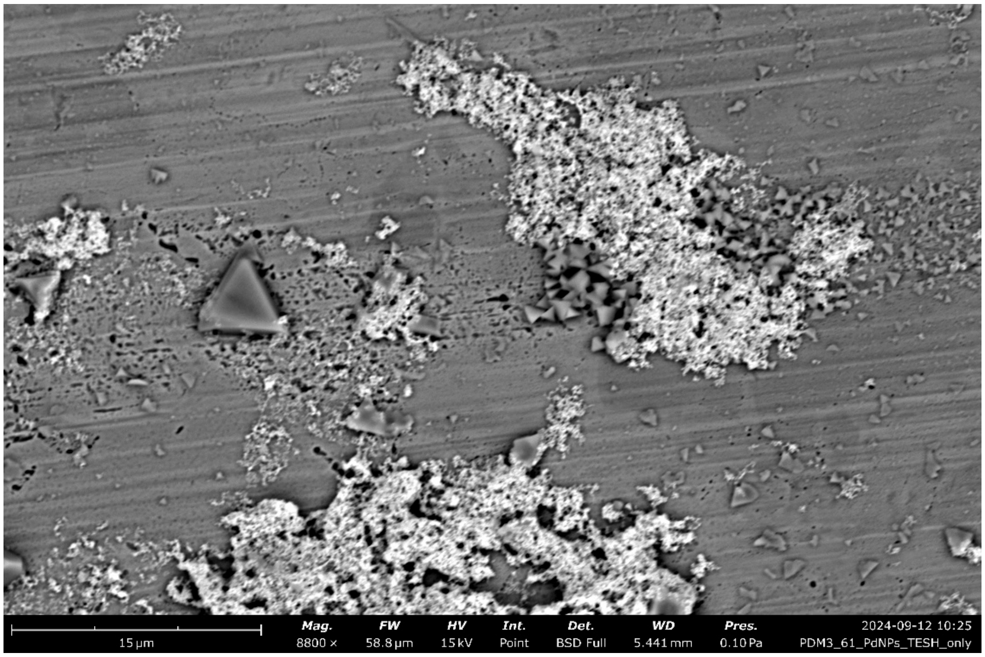

Figure S142. No $B(C_6F_5)_3$ used when reducing Pd(cod)Cl_2 with Et_3Si-H after 2 hours.

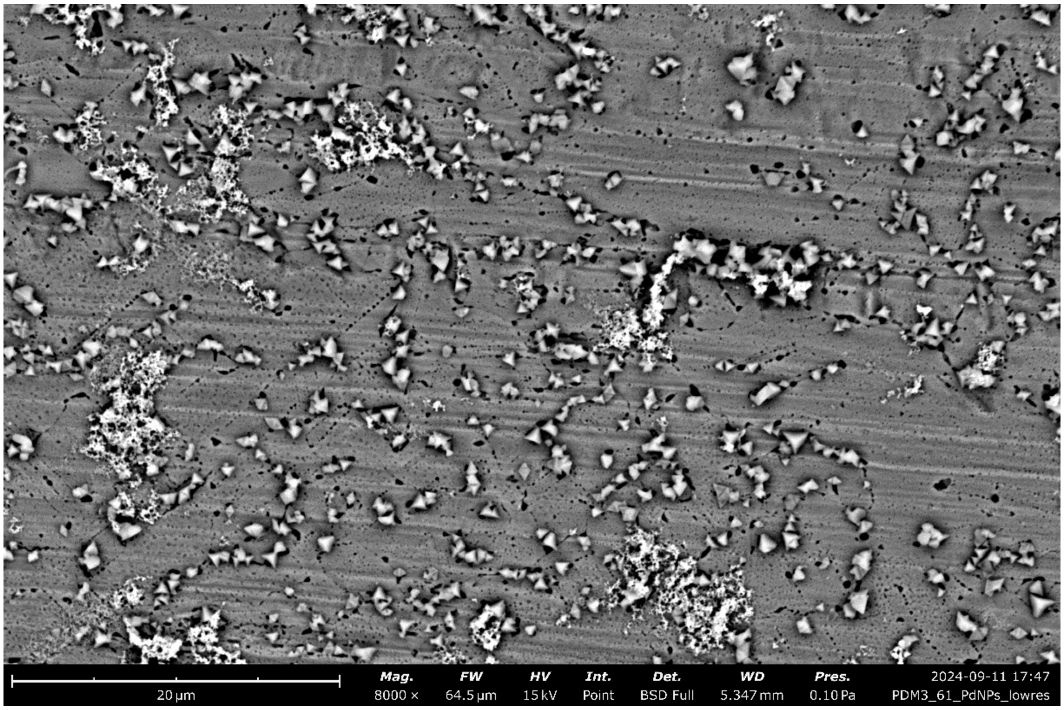

Figure S143. Pd particles made with standard conditions after 2 hours.

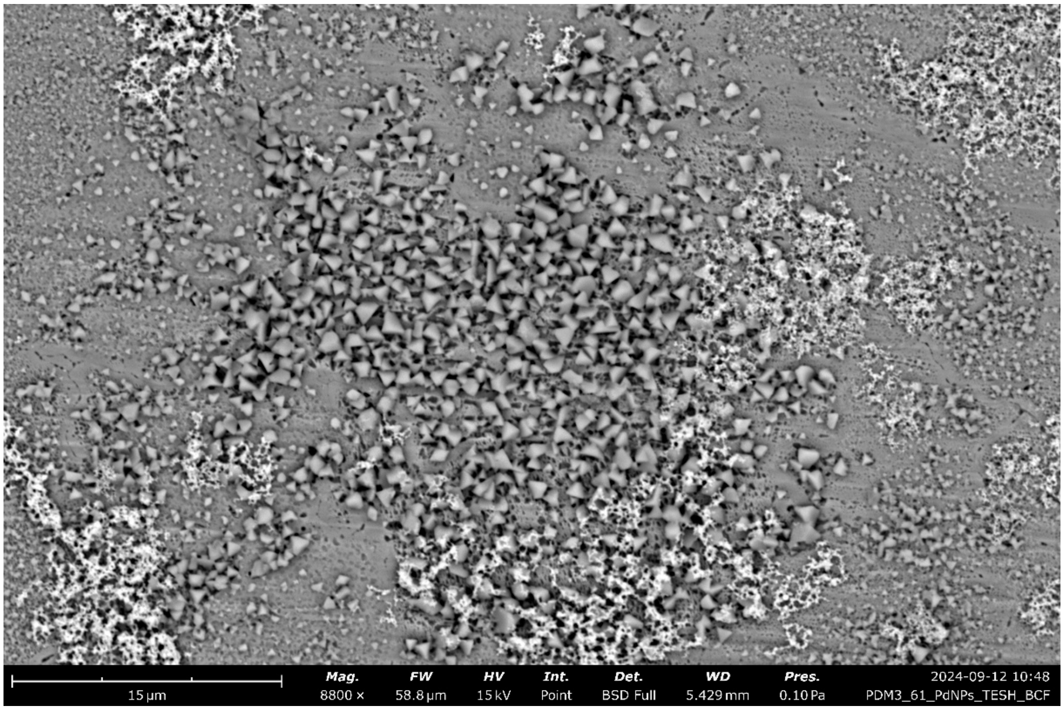

Figure S144. Pd particles made with standard conditions after 2 hours.

References

(1) Collins, K. D.; Glorius, F. A Robustness Screen for the Rapid Assessment of Chemical Reactions. *Nat. Chem.* 2013, *5* (7), 597–601. https://doi.org/10.1038/nchem.1669.

Spectra of Compounds

NMRs of Substrates Post-Isomerization

Substrate: 1-decene

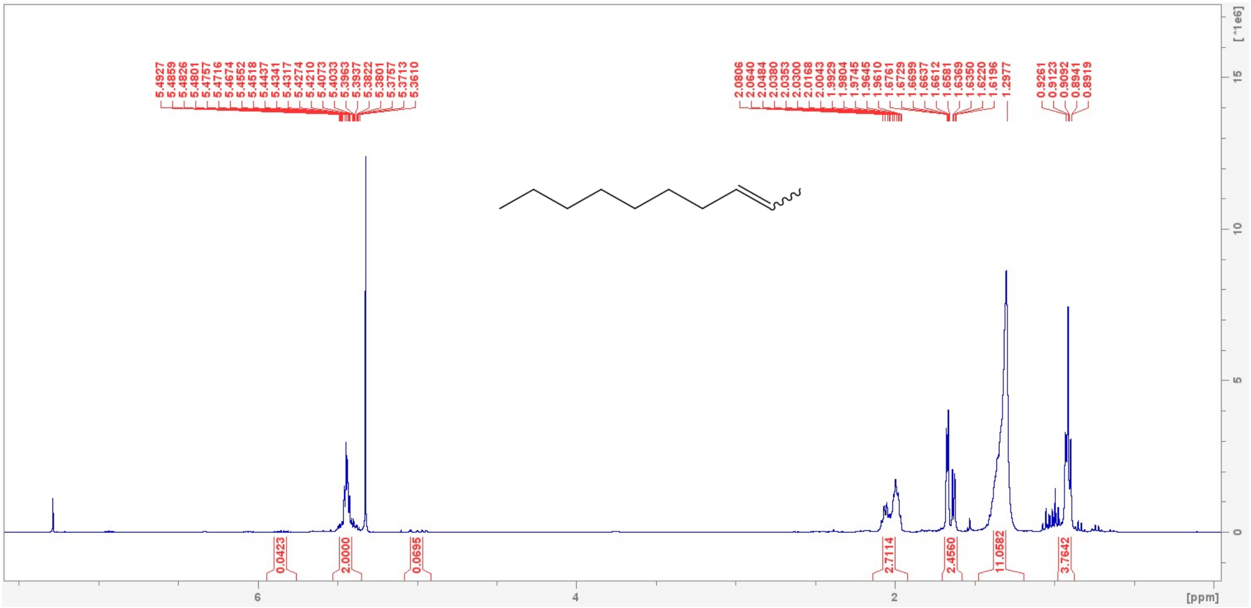

Figure S145. ^{1}H NMR of 1-decene post-isomerization with standard conditions.

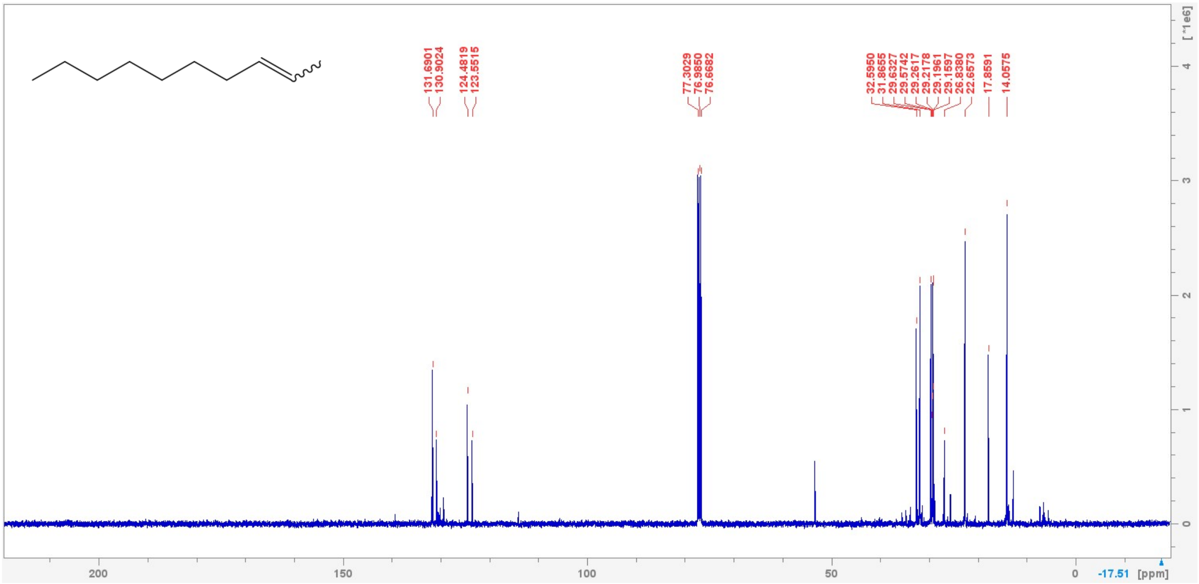

Figure S146. ^{13}C NMR of 1-decene post-isomerization with standard conditions.

Substrate: hex-5-en-1-ylbenzene

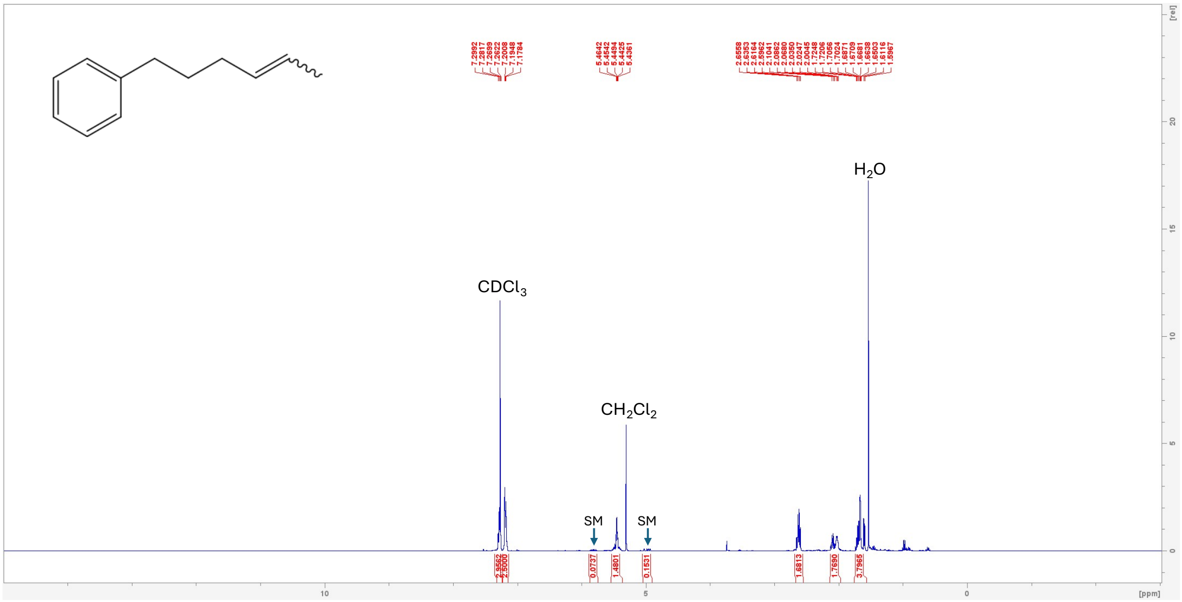

Figure S147. ^{1}H NMR of hex-5-en-1-ylbenzene post-isomerization with standard conditions.

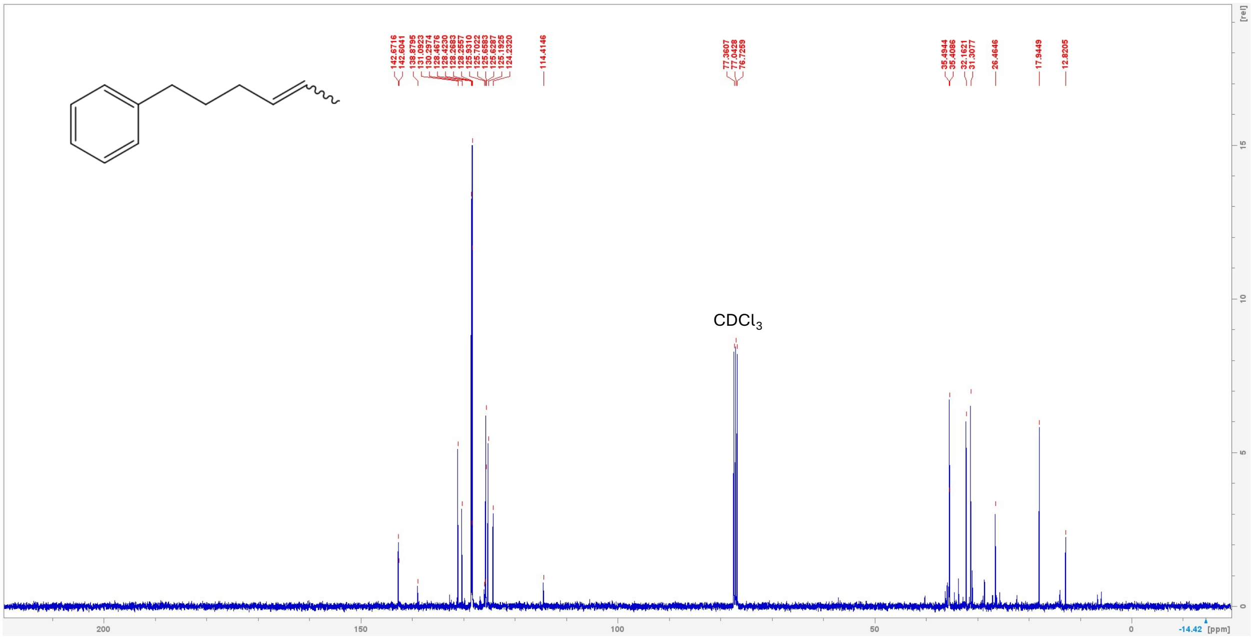

Figure S148. ^{13}C NMR of hex-5-en-1-ylbenzene post-isomerization with standard conditions.

Substrate: but-3-en-1-ylbenzene

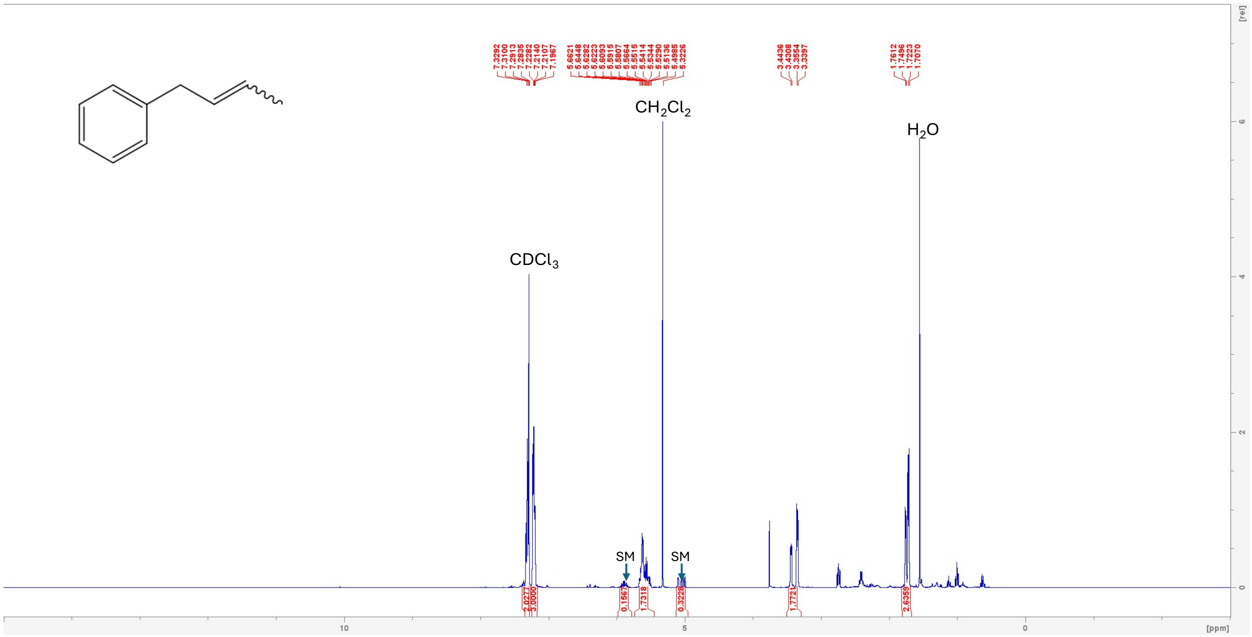

Figure S149. ^{1}H NMR of but-3-en-1-ylbenzene post-isomerization with standard conditions.

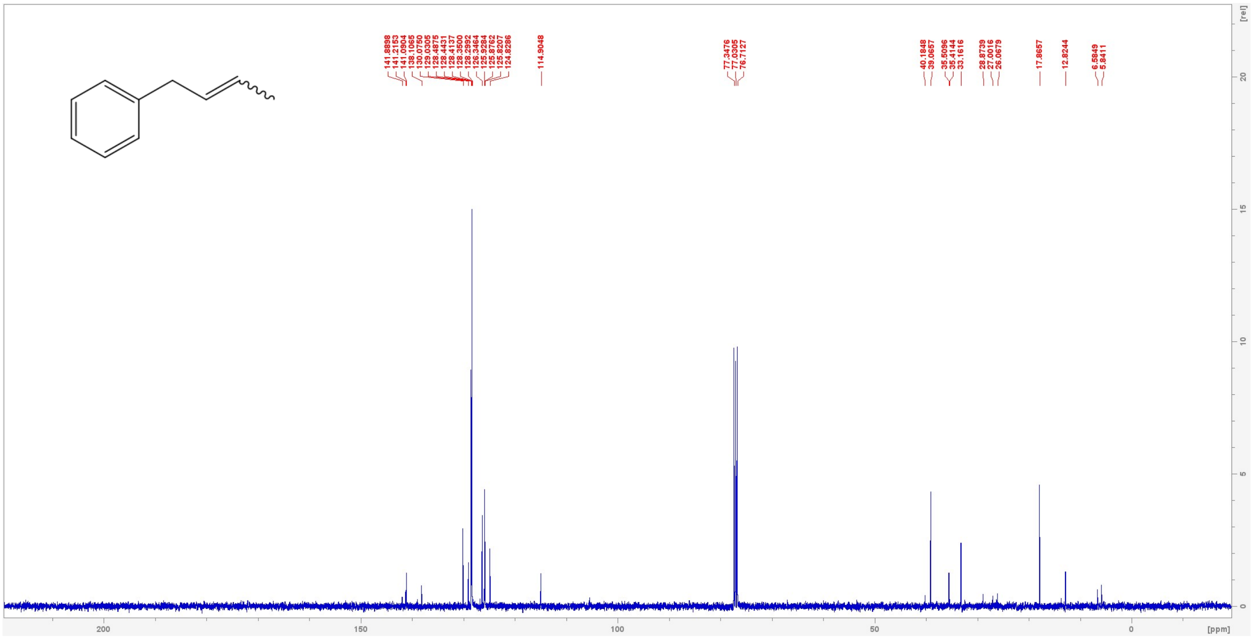

Figure S150. ^{13}C NMR of but-3-en-1-ylbenzene post-isomerization with standard conditions.

Substrate: hex-5-en-1-yl acetate

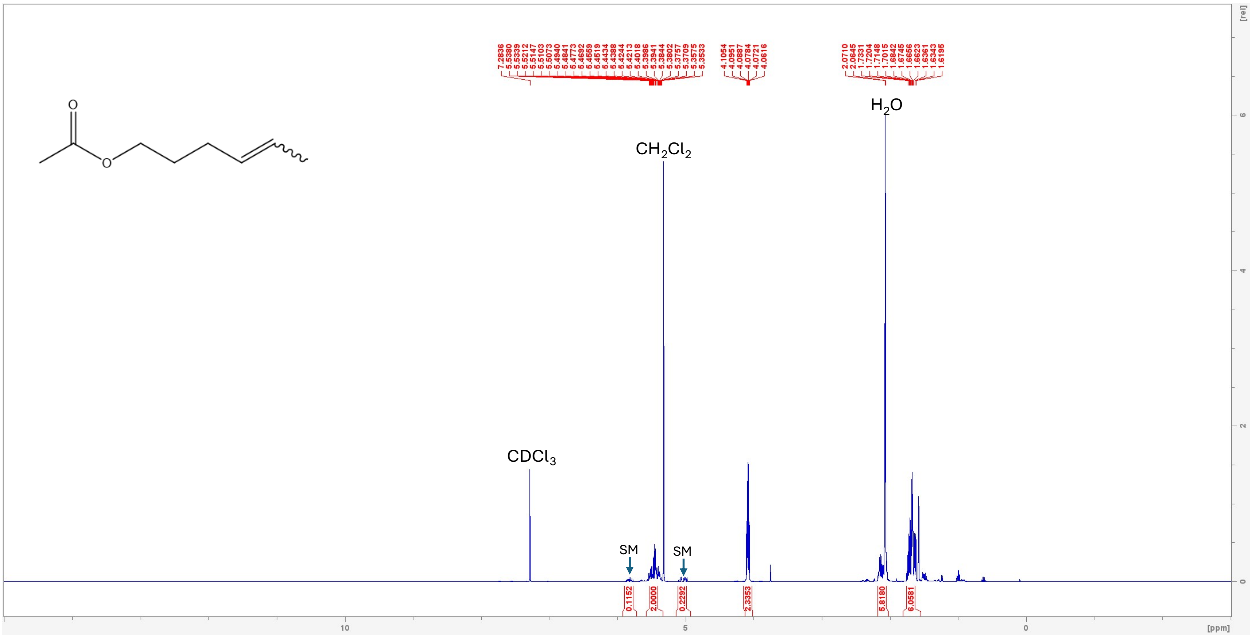

Figure S151. ^{1}H NMR of hex-5-en-1-yl acetate post-isomerization with standard conditions.

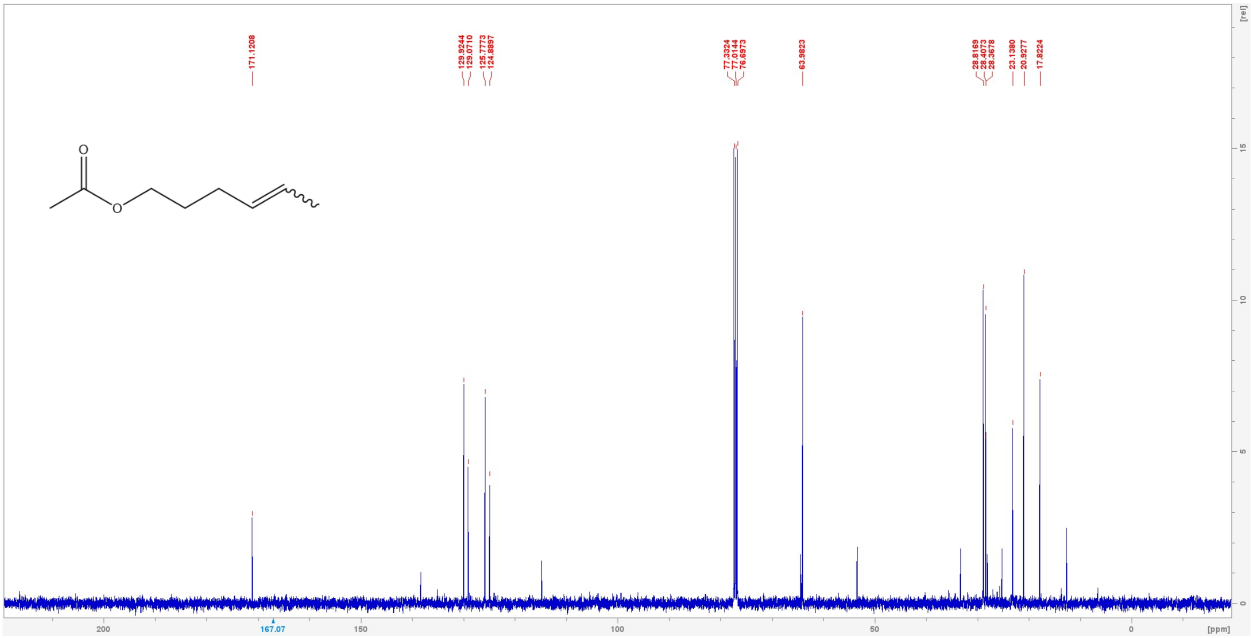

Figure S152. ^{13}C NMR of hex-5-en-1-yl acetate post-isomerization with standard conditions.

Substrate: 6-bromohex-1-ene

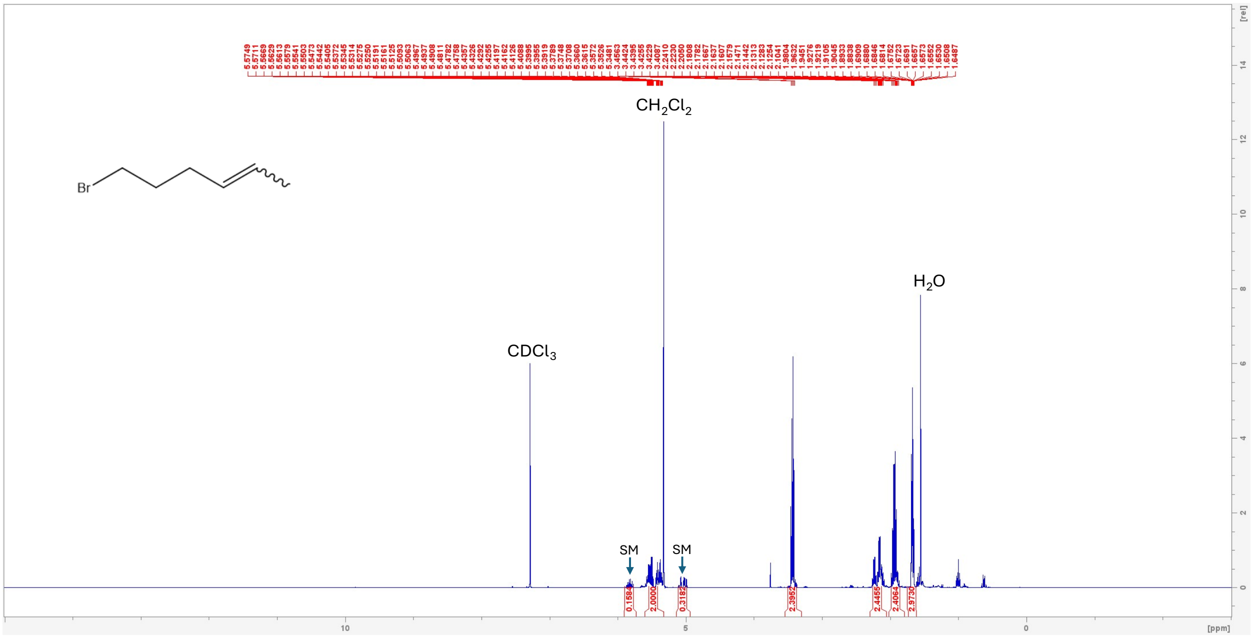

Figure S153. ^{1}H NMR of 6-bromohex-1-ene post-isomerization with standard conditions.

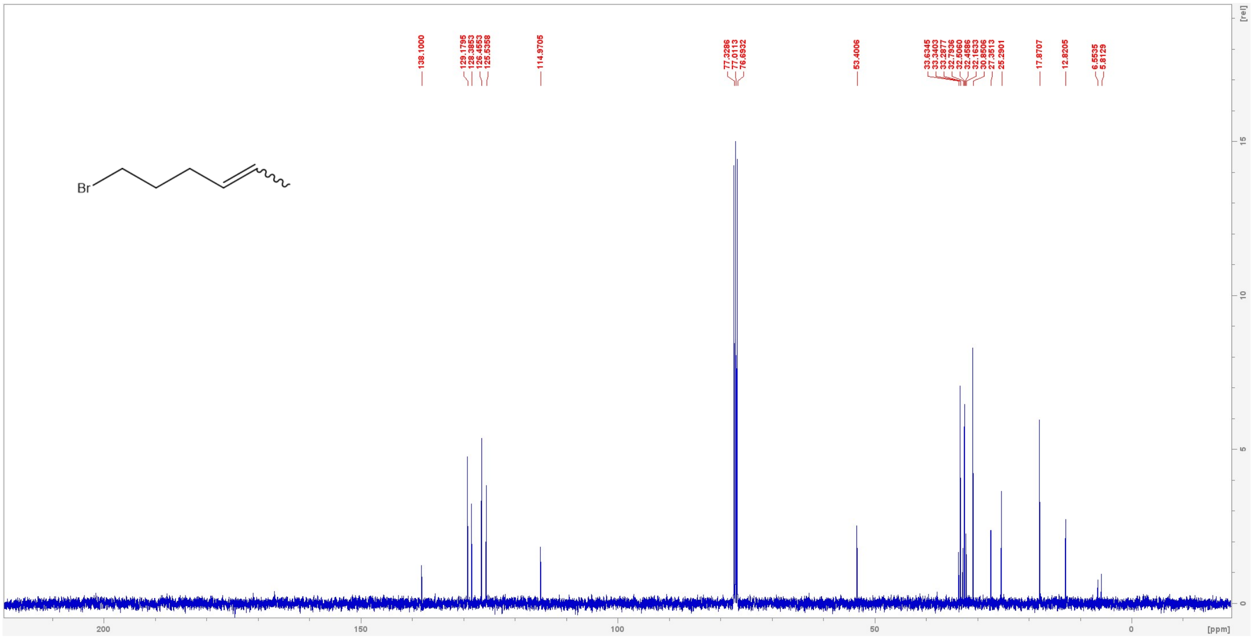

Figure S154. ^{13}C NMR of 6-bromohex-1-ene post-isomerization with standard conditions.

Substrate: allylbenzene

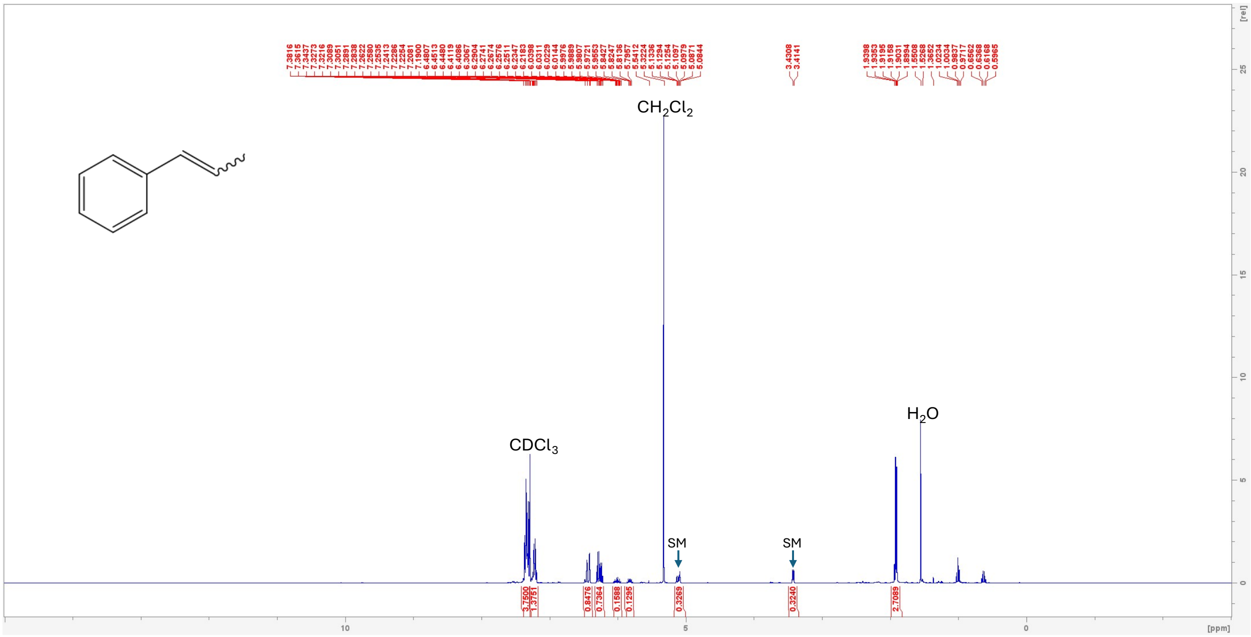

Figure S155. ^{1}H NMR of allylbenzene post-isomerization with standard conditions.

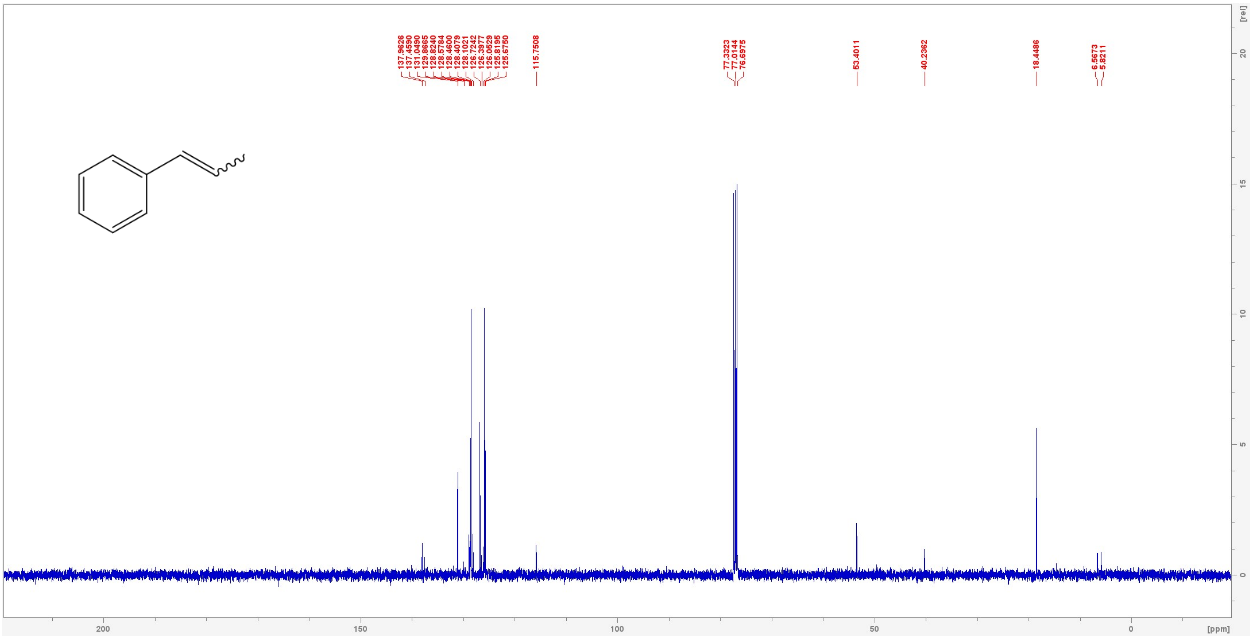

Figure S156. ^{13}C NMR of allylbenzene post-isomerization with standard conditions.

Substrate: 1-octene

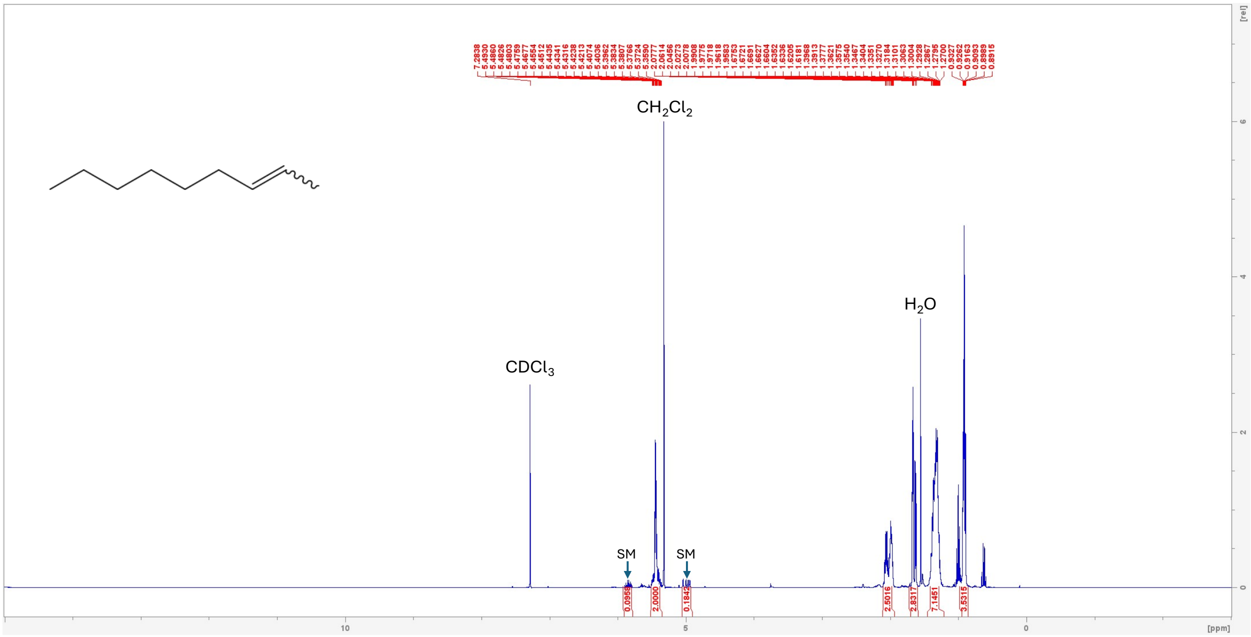

Figure S157. ^{1}H NMR of 1-octene post-isomerization with standard conditions.

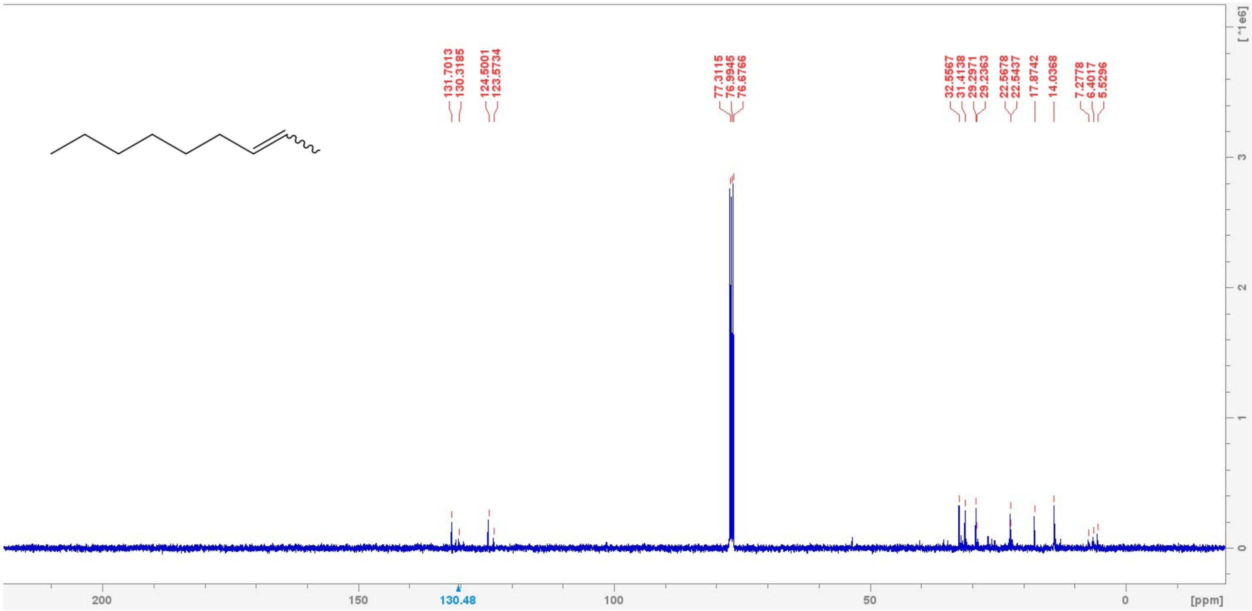

Figure S158. ^{13}C NMR of 1-octene post-isomerization with standard conditions.

Substrate: hex-5-en-1-ol

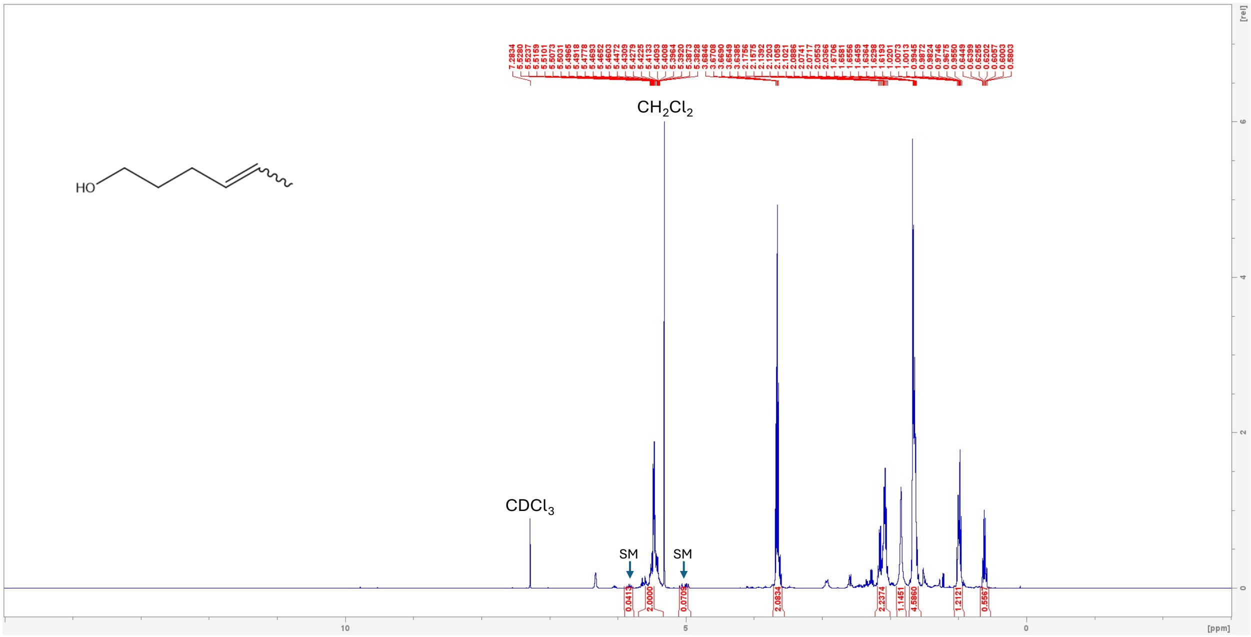

Figure S159. ^{1}H NMR of hex-5-en-1-ol post-isomerization with standard conditions.

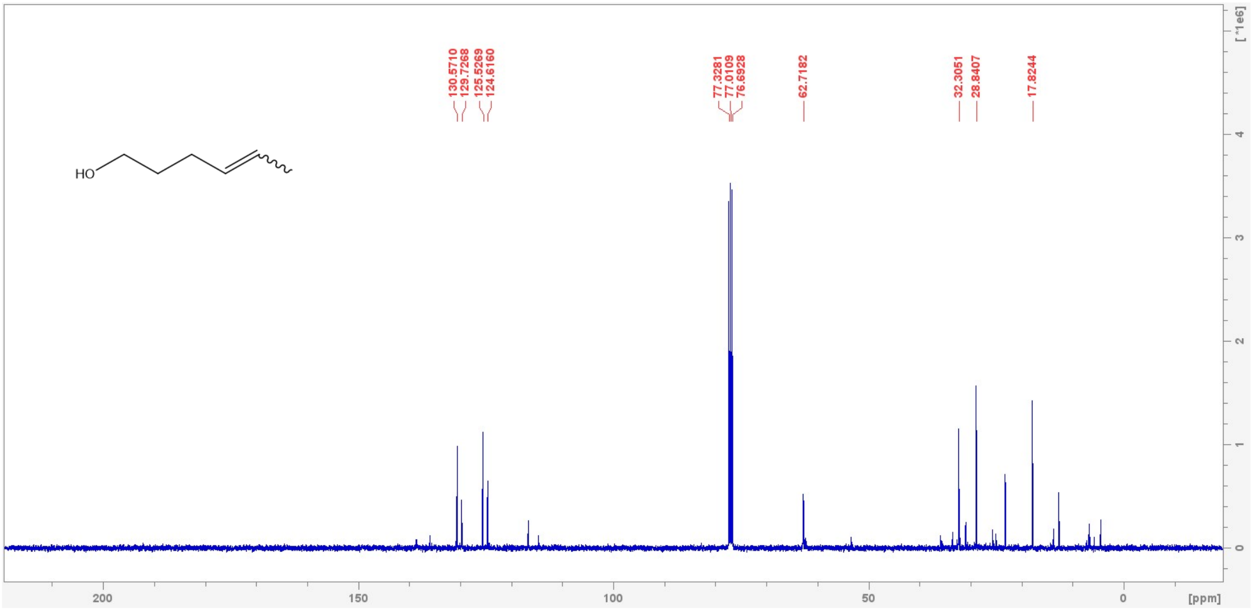

Figure S160. ^{13}C NMR of hex-5-en-1-ol post-isomerization with standard conditions.

Substrate: 2-(hex-5-en-1-yl)thiophene

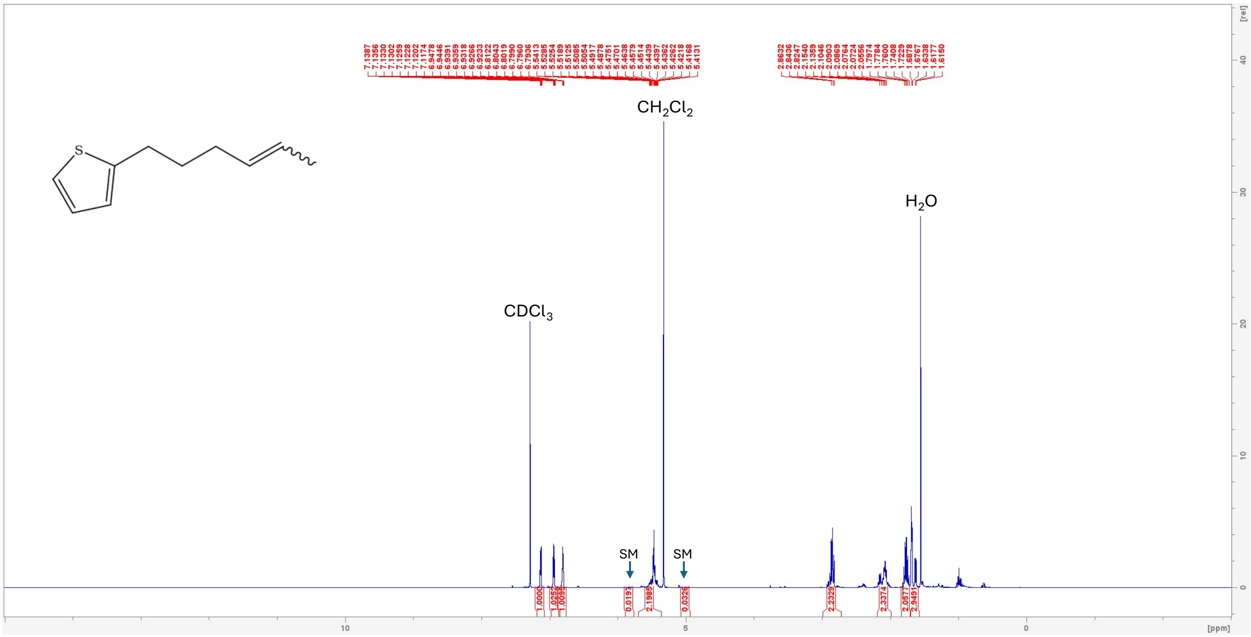

Figure S161. ^{1}H NMR of 2-(hex-5-en-1-yl)thiophene post-isomerization with standard conditions.

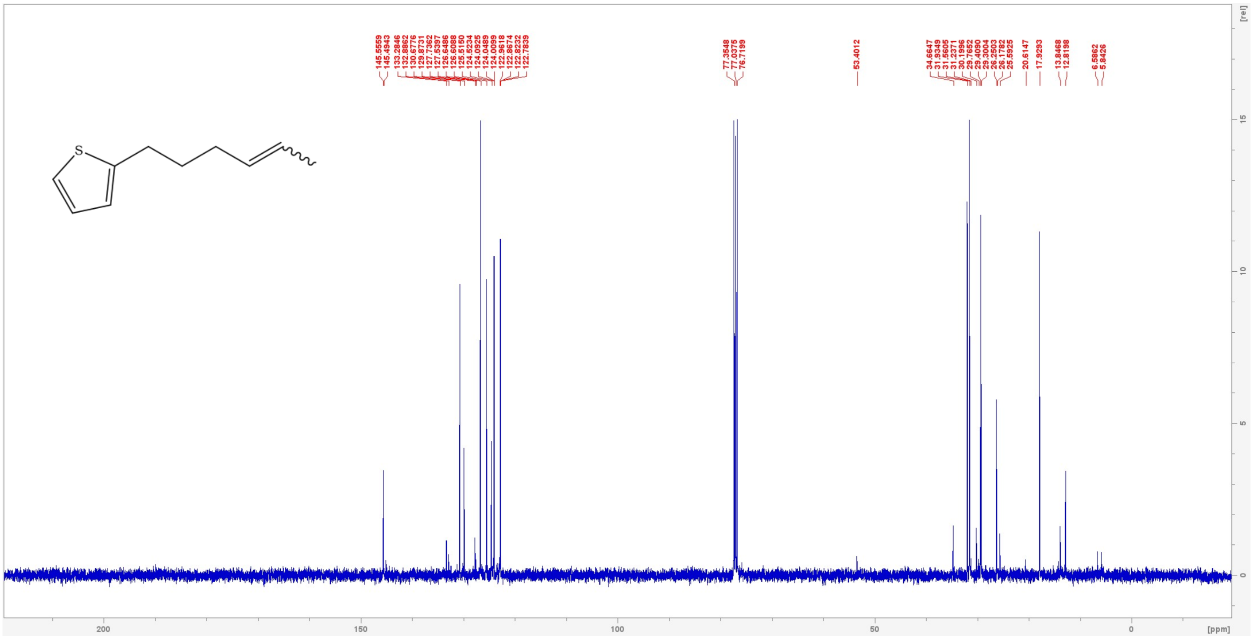

Figure S162. ^{13}C NMR of 2-(hex-5-en-1-yl)thiophenepost-isomerization with standard conditions.

Substrate: triethyl(undec-10-en-1-yloxy)silane

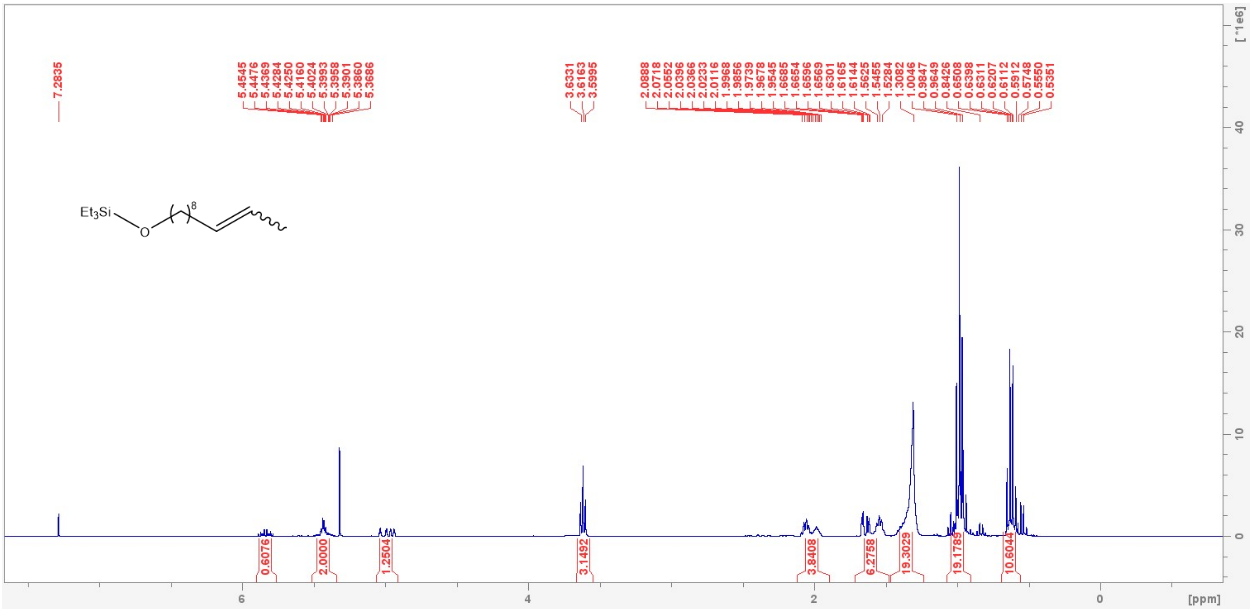

Figure S163. ^{1}H NMR of triethyl(undec-10-en-1-yloxy)silane post-isomerization with standard conditions.

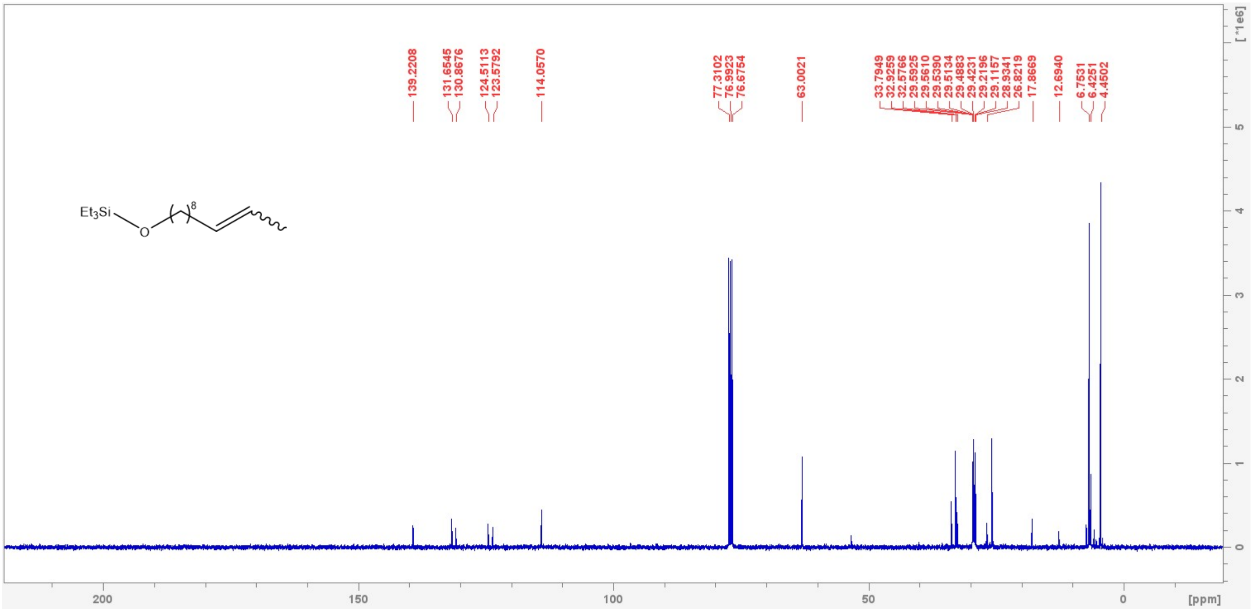

Figure S164. ^{13}C NMR of triethyl(undec-10-en-1-yloxy)silane post-isomerization with standard conditions.

Substrate: tert-butyldimethyl(undec-10-en-1-yloxy)silane

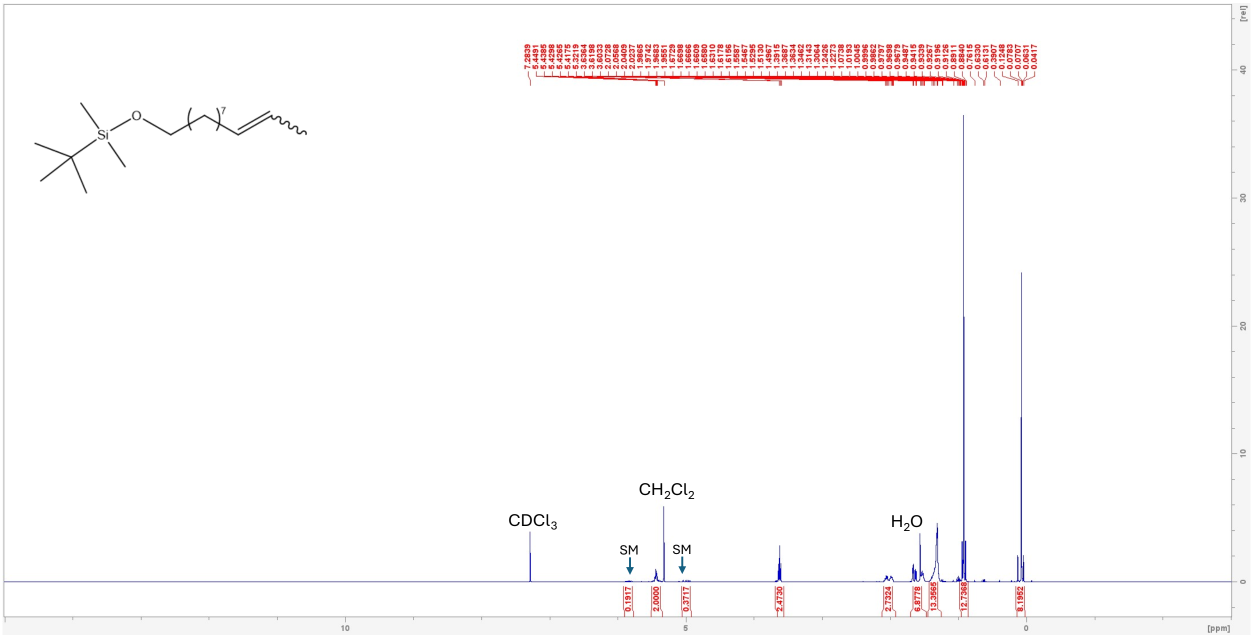

Figure S165. ^{1}H NMR of tert-butyldimethyl(undec-10-en-1-yloxy)silane post-isomerization with standard conditions.

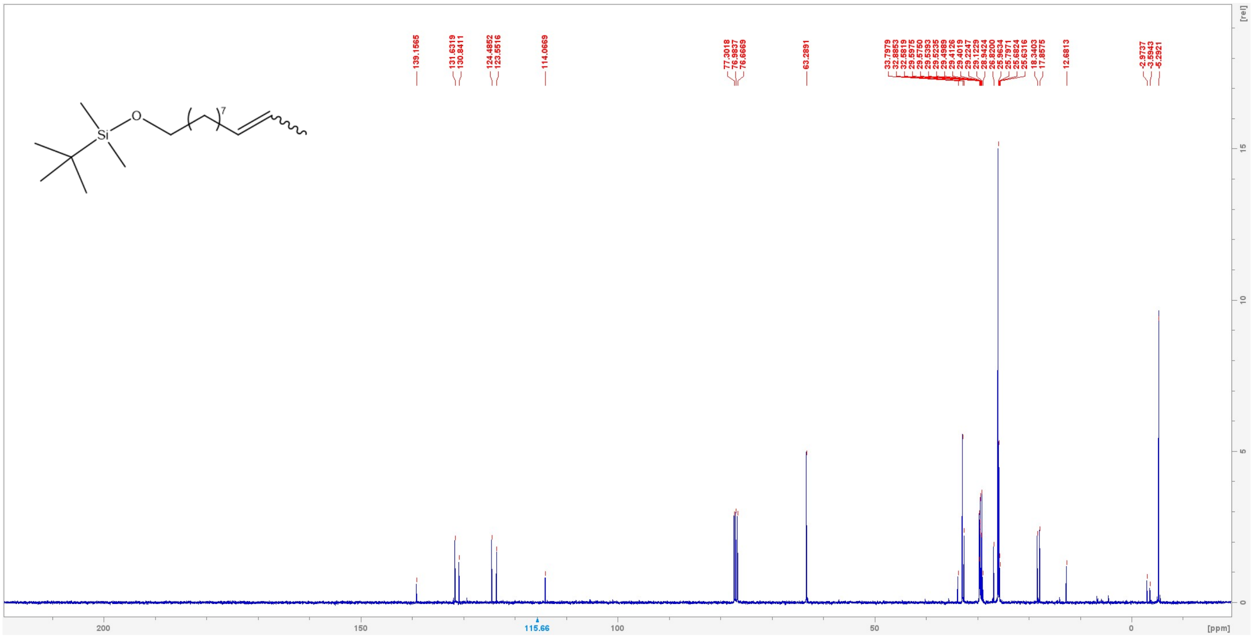

Figure S166. ^{13}C NMR of tert-butyldimethyl(undec-10-en-1-yloxy)silanepost-isomerization with standard conditions.

Substrate: 6-methoxyhex-1-ene

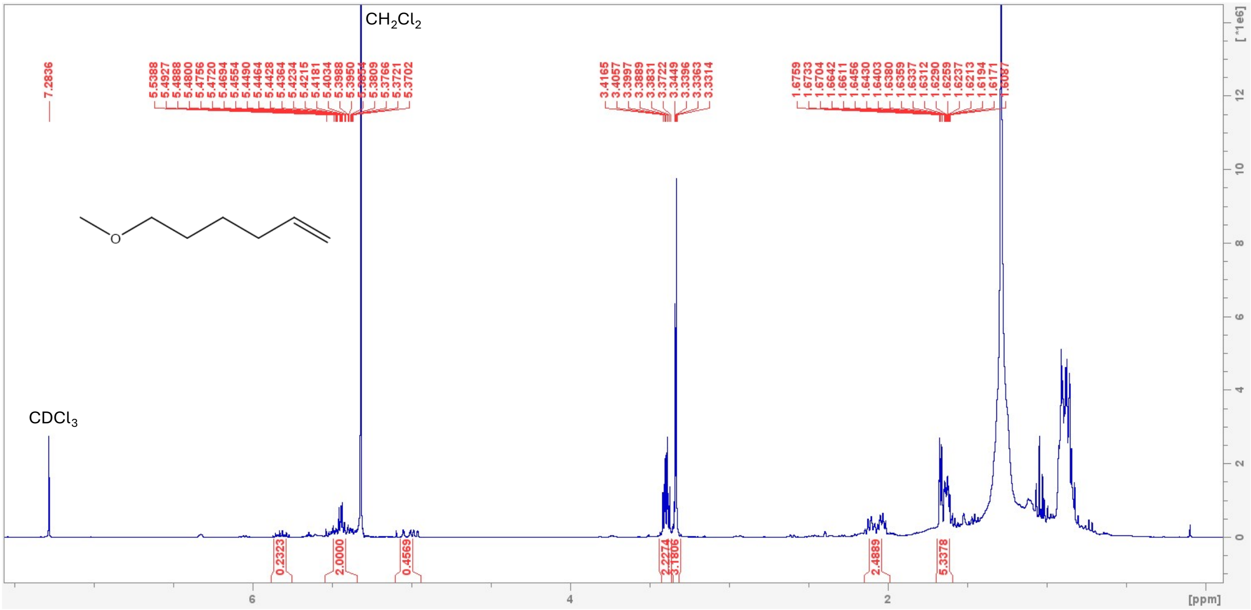

Figure S167. ^{1}H NMR of 6-methoxyhex-1-ene post-isomerization with standard conditions.

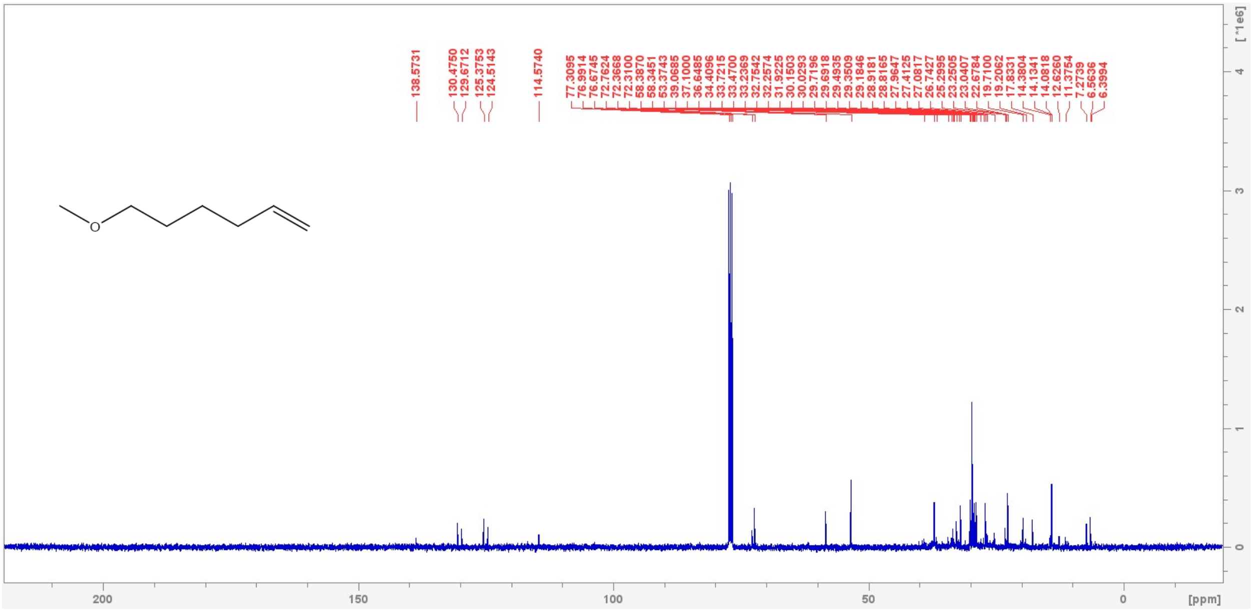

Figure S168. ^{13}C NMR of 6-methoxyhex-1-ene post-isomerization with standard conditions.

Substrate: 6-chlorohex-1-ene

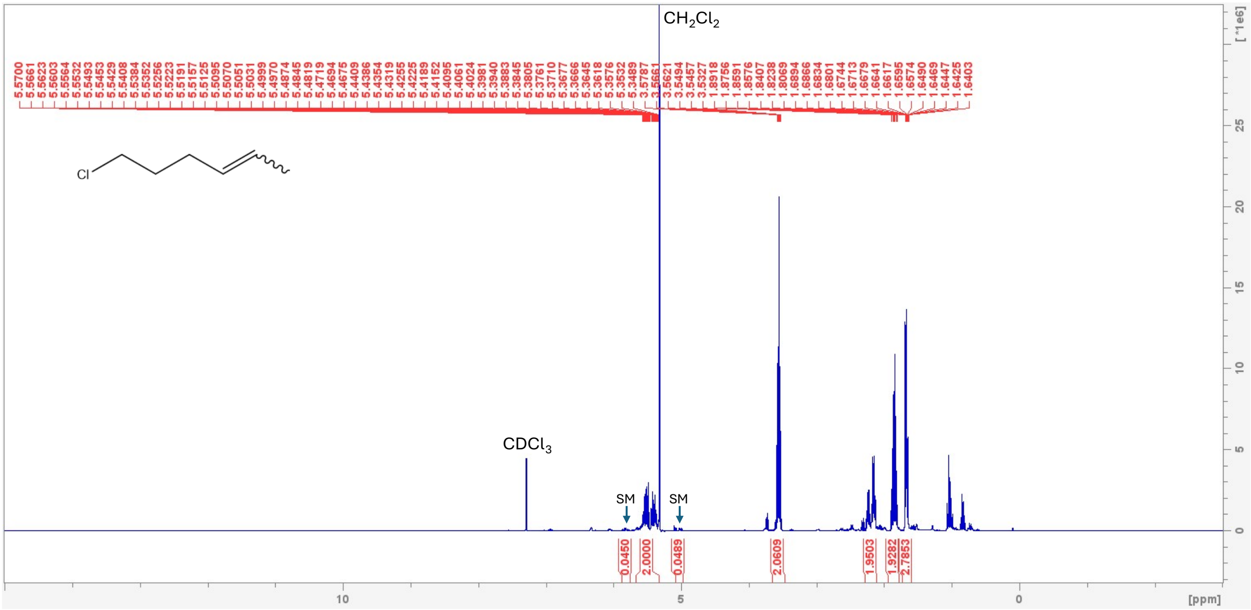

Figure S169. ^{1}H NMR of 6-chlorohex-1-ene post-isomerization with standard conditions.

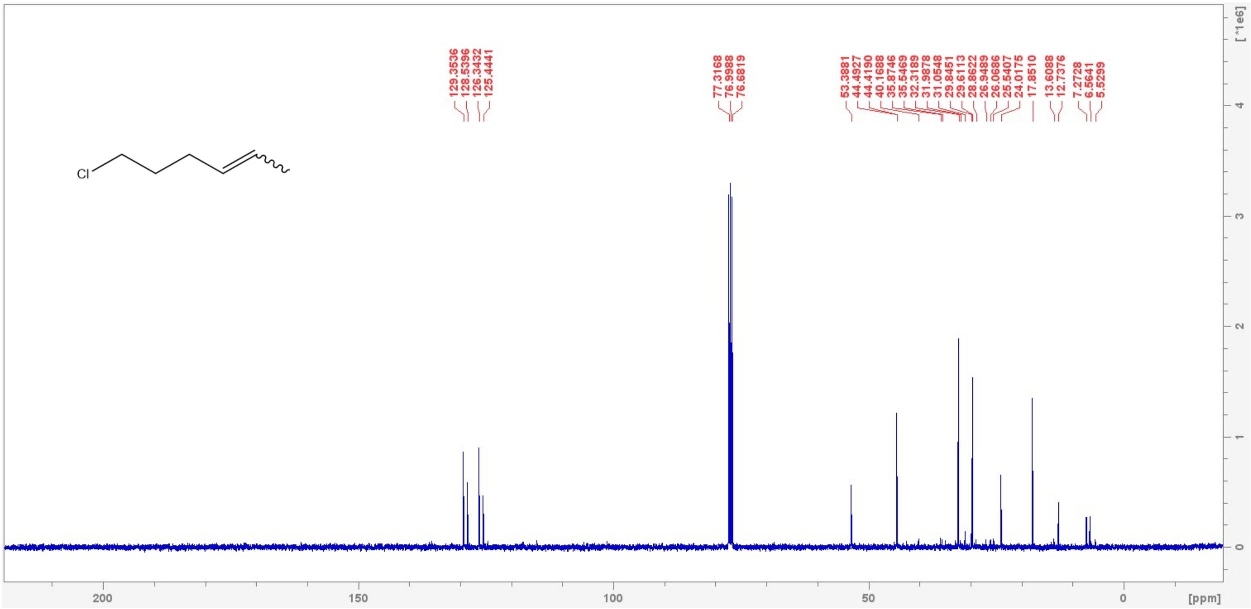

Figure S170. ^{13}C NMR of 6-chlorohex-1-ene post-isomerization with standard conditions.

Substrate: hex-5-en-1-yl 2-(4-isobutylphenyl)propanoate

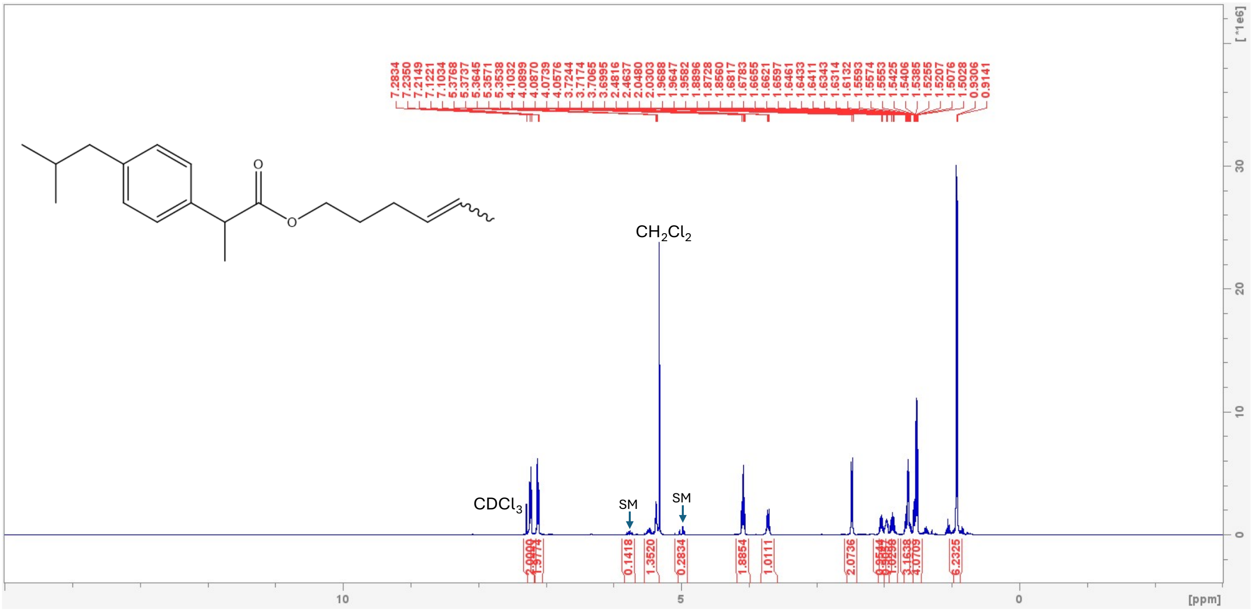

Figure S171. ^{1}H NMR of hex-5-en-1-yl 2-(4-isobutylphenyl)propanoate post-isomerization with standard conditions.

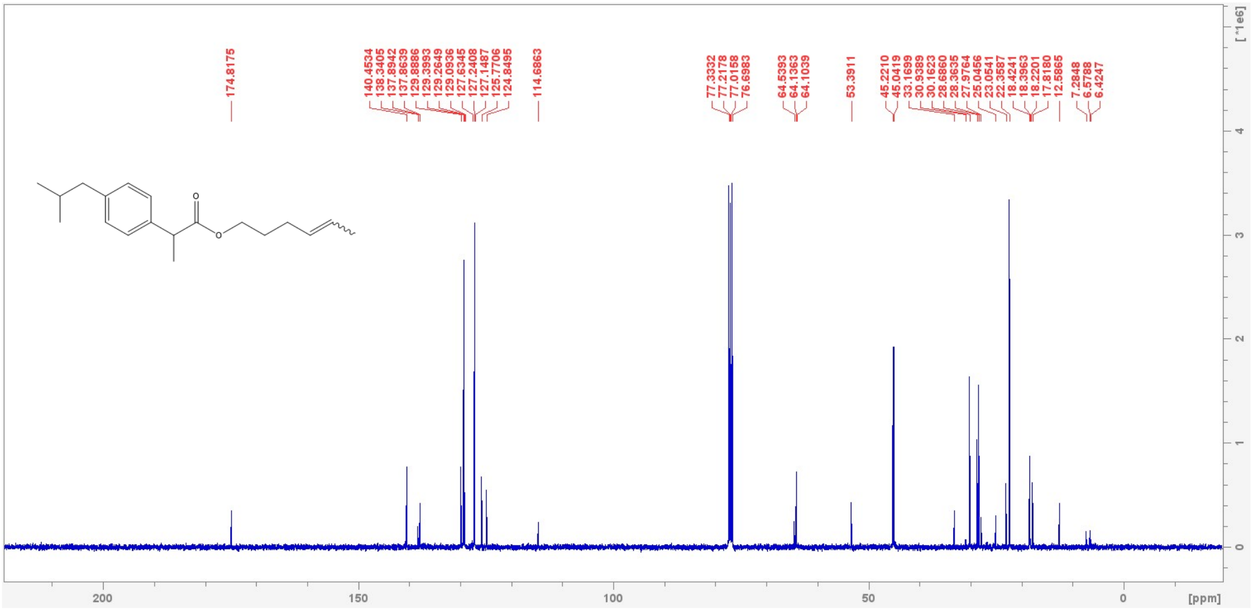

Figure S172. ^{13}C NMR of hex-5-en-1-yl 2-(4-isobutylphenyl)propanoate post-isomerization with standard conditions.

hex-5-en-1-yl 2-(4-isobutylphenyl)propanoate

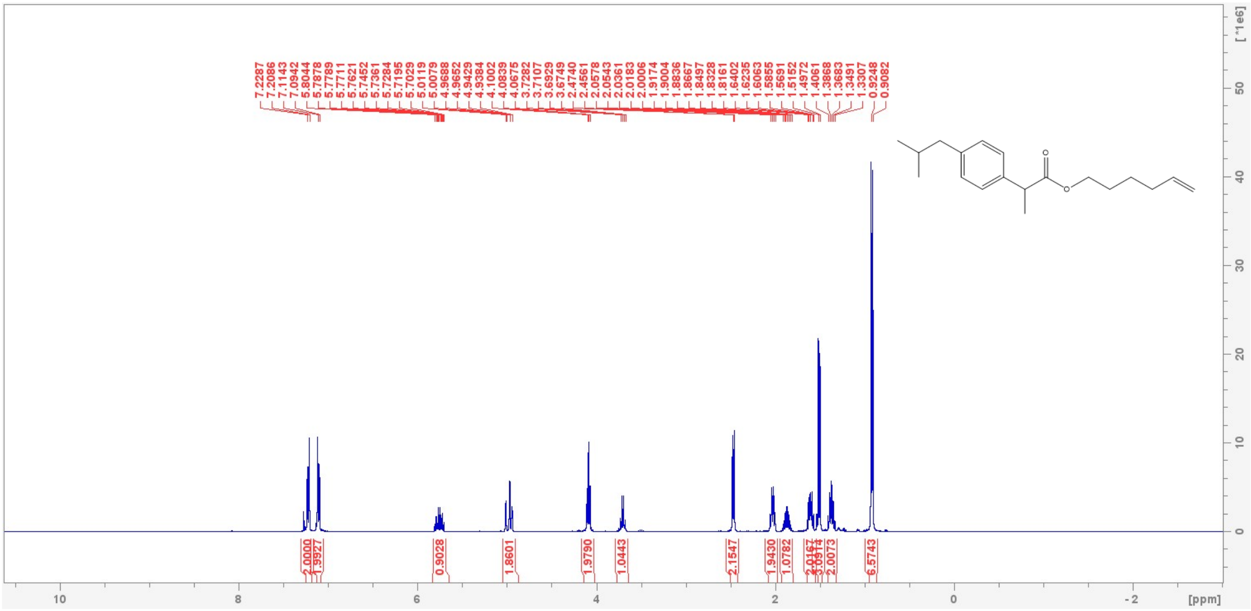

Figure S173. ^{1}H NMR of hex-5-en-1-yl 2-(4-isobutylphenyl)propanoate.

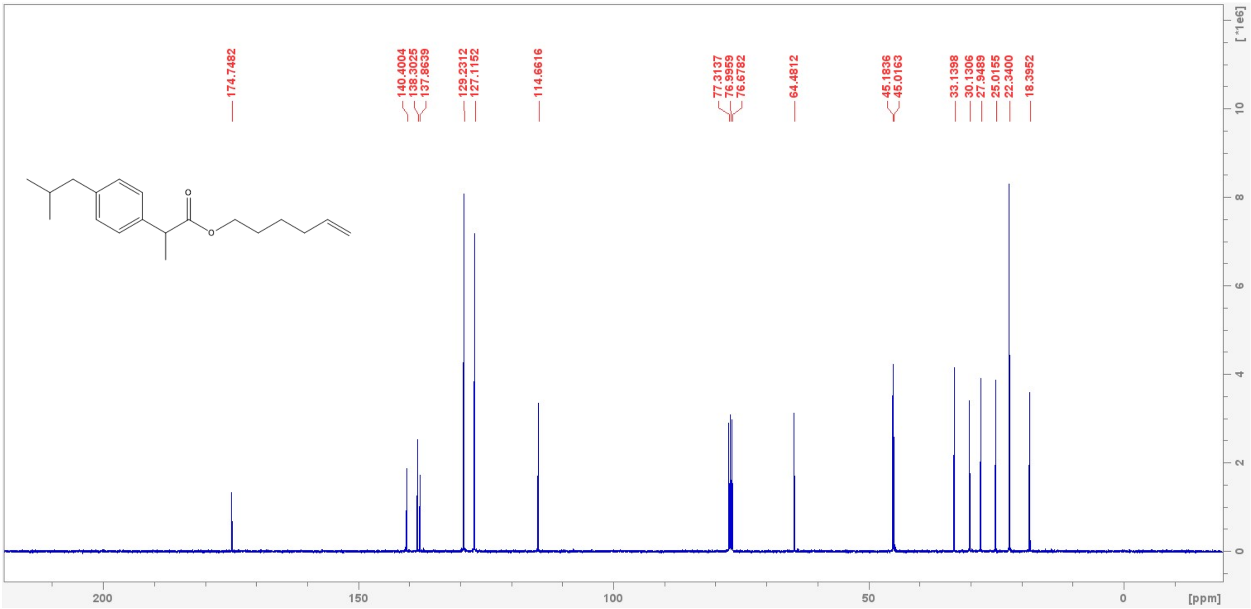

Figure S174. ^{13}C NMR of hex-5-en-1-yl 2-(4-isobutylphenyl)propanoate.

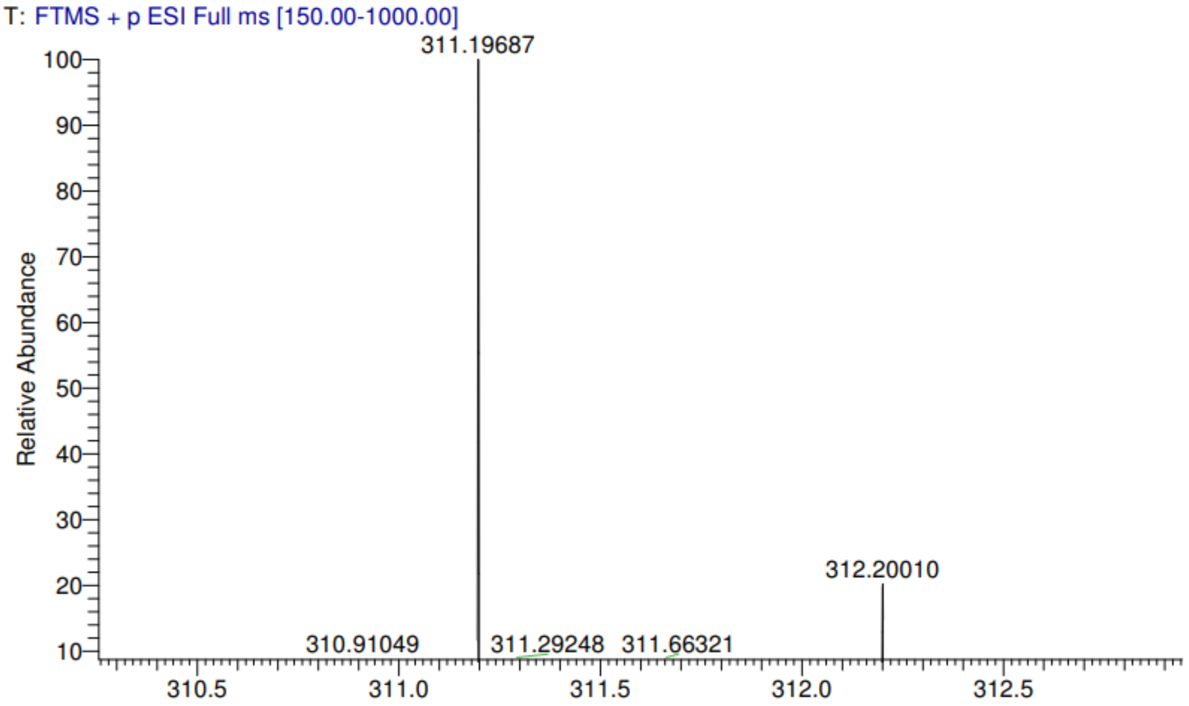

Figure S175. HRMS of hex-5-en-1-yl 2-(4-isobutylphenyl)propanoate.

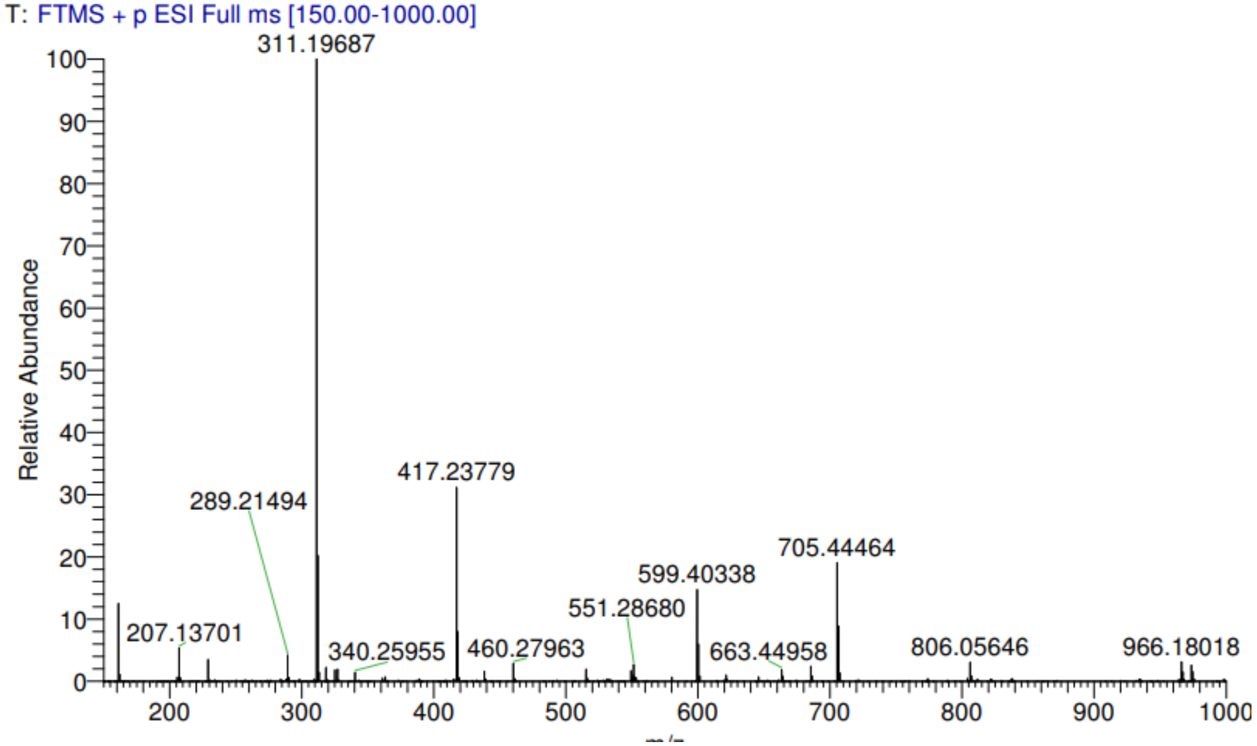

Figure 176. HRMS of hex-5-en-1-yl 2-(4-isobutylphenyl)propanoate (Full View).
